# Liquid Phase Peptide Synthesis via One‐Pot Nanostar Sieving (PEPSTAR)

**DOI:** 10.1002/anie.202014445

**Published:** 2021-02-24

**Authors:** Jet Yeo, Ludmila Peeva, Seoyeon Chung, Piers Gaffney, Daeok Kim, Carla Luciani, Sergey Tsukanov, Kevin Seibert, Michael Kopach, Fernando Albericio, Andrew Livingston

**Affiliations:** ^1^ Barrer Centre Department of Chemical Engineering Imperial College London Exhibition Road London SW7 2AZ UK; ^2^ Eli Lilly and Company RNA-Therapeutics 450 Kendal St Cambridge MA 02142 USA; ^3^ Eli Lilly and Company Synthetic Molecule Design & Development 1200 W Morris St Indianapolis IN 46221 USA; ^4^ Networking-Centre on Bioengineering, Biomaterials and Nanomedicine Department of Organic Chemistry University of Barcelona Marti i Franques 1–11 08028 Barcelona Spain; ^5^ School of Chemistry and Physics University of KwaZulu-Natal University Road, Westville Campus Durban 4001 South Africa; ^6^ School of Engineering and Materials Science Queen Mary University of London Mile End Rd, Bethnal Green London E1 4NS UK

**Keywords:** liquid phase peptide synthesis, membranes, organic solvent nanofiltration (OSN), peptides

## Abstract

Herein, a one‐pot liquid phase peptide synthesis featuring iterative addition of amino acids to a “nanostar” support, with organic solvent nanofiltration (OSN) for isolation of the growing peptide after each synthesis cycle is reported. A cycle consists of coupling, Fmoc removal, then sieving out of the reaction by‐products via nanofiltration in a reactor‐separator, or synthesizer apparatus where no phase or material transfers are required between cycles. The three‐armed and monodisperse nanostar facilitates both efficient nanofiltration and real‐time reaction monitoring of each process cycle. This enabled the synthesis of peptides more efficiently while retaining the full benefits of liquid phase synthesis. PEPSTAR was validated initially with the synthesis of enkephalin‐like model penta‐ and decapeptides, then octreotate amide and finally octreotate. The crude purities compared favorably to vendor produced samples from solid phase synthesis.

## Graphical abstract

PEPSTAR, a “rising star” in peptide synthesis, features a “nanostar” support to grow peptides with organic solvent nanofiltration for isolation. The three‐armed and monodisperse nanostar enables efficient nanofiltration and real‐time reaction monitoring. A cycle consists of coupling, Fmoc removal and nanofiltration, which all performed continuously in a synthesizer to produce high purity peptide crudes through a one‐pot approach.

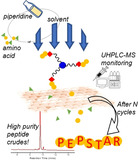

## Introduction

Recent strategies to improve the pharmacokinetics and oral availability of peptide drugs have created a renaissance in peptide therapeutics. With more than 70 peptide drugs approved for clinical use and many in the development pipeline, global peptide therapeutic demand is rising.^[1]^ This trend has created an imperative for continuing innovation in peptide manufacturing technology in anticipation of growing future demand for production of peptide therapeutics.

When the exact sequence of monomers in a polymer is crucial, as in peptide synthesis, chemical approaches utilize iterative synthesis with protecting group chemistry to ensure high sequence precision, with separation of unreacted monomers from the growing polymer at each cycle being the crucial step. Solid phase attachment solves this key separation problem for peptide synthesis, enabling excess amino acids to be washed away and separated from the support‐bound growing peptide. Solid phase peptide synthesis (SPPS) provides for facile manipulation of intermediates retained in the solid support, and facilitates process automation and rapid synthesis in a single reactor.^[2]^ Hence SPPS is widely regarded as the gold standard for peptide synthesis. However, the intraparticle reactions also bring penalties. These include diffusional limitations in the solid supports leading to incomplete couplings and deprotections as well as the use of reagent in large excesses to drive the diffusion and reaction in the solid phase.^[3]^ Liquid phase peptide synthesis (LPPS) could potentially accomplish higher crude purity, lower reagent consumption, and greater ease of scaling than SPPS. However, these advantages have not so far overtaken SPPS because the key separation of intermediates from reaction by‐products is usually achieved by precipitation or extraction. These relatively time consuming operations must often be optimized from one cycle to the next, and from one target to another, as the physical properties of growing peptide intermediates vary unpredictably, making the overall process slow and tedious.^[4]^

The first LPPS strategy to address these deficits attempted to standardize the process by anchoring all intermediates permanently in an organic phase, removing by‐products by extraction or precipitation, and with sequential couplings and deprotections requiring just one isolation step at the end of synthesis.^[5]^ To further control the solubility of growing peptides, recent developments in hydrophobic anchors for LPPS have increased the practicality of extraction and precipitation approaches.[^5a^, ^5b^, ^5c^, ^5d^, ^5e^] These works demonstrated the expected fast coupling kinetics, and utility of reaction monitoring to ensure high purity, but precipitation and extraction methods have still not been widely adopted. Multi‐step work‐ups, such as washing with several aqueous phases, and material transfer between apparatus lack process continuity (Figure S1). Generally, isolation via phase transfer, either liquid‐to‐liquid or liquid‐to‐solid, is challenging for scale‐up, thus prolonging process development lead‐time and impeding automation.^[6]^

One pot (i.e. telescoped) LPPS with no phase or material transfer between synthesis cycles would be ideal for peptide manufacturing. An LPPS approach built around membrane‐based separation, or diafiltration, might provide this. Bayer et al. were the first to describe this approach, but their process was impractical due to the lack of solvent stable membranes; solvent exchange into water was required prior to purification using a dialysis membrane.^[7]^ Membrane‐based LPPS is greatly facilitated if the separations can be conducted in the same organic solvent the reactions are performed in, and recent advances in organic solvent nanofiltration (OSN) have brought this approach within reach.^[8]^

Membrane‐enhanced peptide synthesis (MEPS), was introduced by So et al. employing a linear 5 kDa methoxy polyethylene glycol (mPEG) anchor to maximize peptide retention.^[9]^ After each coupling then deprotection step, reaction by‐products and excess reagents were permeated through a solvent stable membrane, while the growing peptide‐mPEG conjugate was retained by the membrane within a combined reactor‐separator. Although the MEPS concept was validated through the synthesis of a pentapeptide, the process was compromised by relatively low mass efficiency and low anchor loading capacity (≈0.2 mmol g^−1^). Excessively large linear mPEG was required to minimize yield loss through the membrane attributed to the ability of PEG to adopt an extended, instead of a globular random coil, conformation that was membrane permeable.^[10]^ Recently, MEPS was revisited using a large permanently globular anchor (≈6–8 kDa) to improve the separation.^[6b]^ Despite having multiple conjugation sites at the ends of four or five polymer arms, the loading capacity of the anchor was low (≈0.6 mmol g^−1^). Furthermore, the polydispersity of the anchor resulted in a broad molecular weight distribution and diffuse range of properties, making quantitative reaction monitoring technically challenging, negating one potential major advantage of LPPS. To the best of our knowledge, no unimolecular anchor has been reported for membrane‐based LPPS.^[11]^ We believe that these limitations have deterred the wider adoption and development of MEPS.

**Figure 1:**
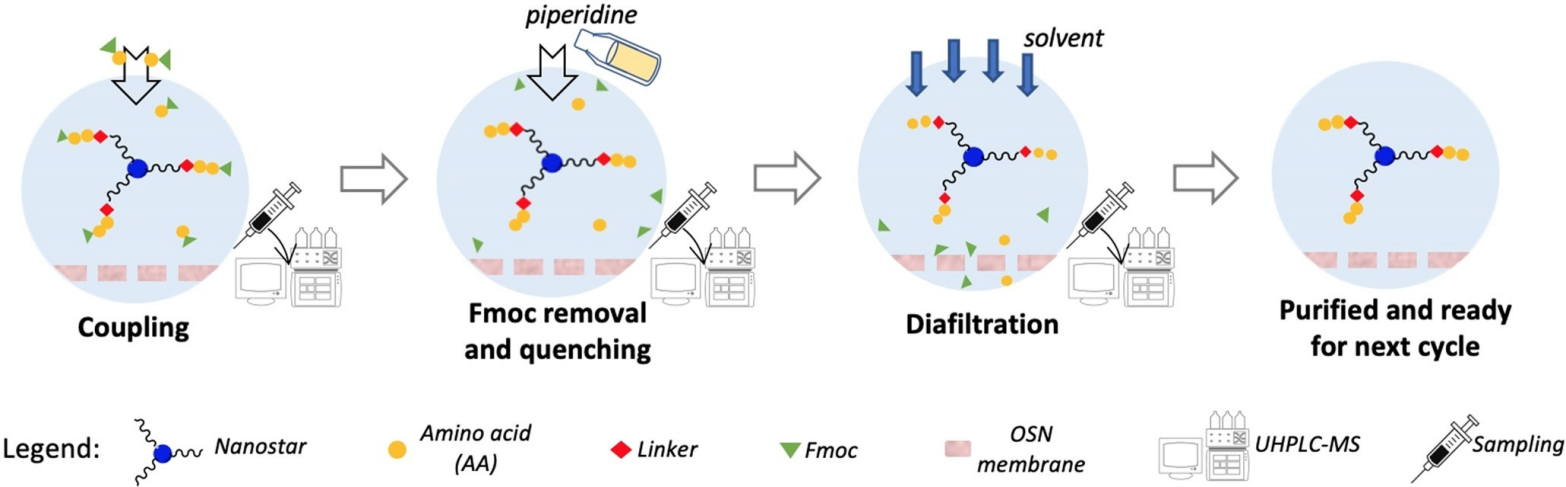
Key features of PEPSTAR: Activated Fmoc‐amino acid building blocks couple to chain termini of H‐peptide‐nanostar; piperidine quenches excess amino acid and removes Fmoc; diafiltration washes all reaction by‐products through a membrane; purified H‐peptide‐nanostar is retained in the synthesizer, ready to repeat the cycle. Every process is analyzed in real time by UHPLC‐MS.

In this report, inspired by recent successes in membrane‐based liquid phase synthesis of sequence‐defined polymers,^[12]^ we introduce liquid phase **pep**tide synthesis via one‐pot nano**star**‐sieving (**PEPSTAR**, Figure 1). Several innovations are incorporated to address the shortcomings identified in earlier membrane‐based LPPS protocols: 1) A compact, monodisperse “nanostar” hub was designed; 2) real‐time reaction monitoring by UHPLC‐MS was undertaken; and 3) an efficient one‐pot process operation was developed. This new LPPS concept is promising for development into a fully automated peptide manufacturing technology.

## Results and Discussion

**Nanostar design and synthesis**. A compact, easily synthesized hub was needed that would nevertheless increase the molecular size of peptide intermediates for efficient nanofiltration. A three‐armed, star‐shaped molecule, or “nanostar”, was selected to provide the desired difference in molecular weight between the growing peptide and the reaction by‐products, while at the same time minimizing the number of chain extension intermediates needing to be resolved during UHPLC‐MS reaction monitoring. The three growing peptides are linked by an aromatic hub, which also acts as an additional UV chromophore, by flexible, monodisperse octaethylene glycol (octagol, HO‐Eg_8_‐OH) spacers, to limit steric interaction between adjacent peptides^[13]^ (Figure 1). Compared to earlier MEPS strategies, this design increases the loading capacity of the anchor with three peptide attachment sites; increases the mass efficiency due to the low molecular weight of the anchor; and enhances the separation due to the at least 3‐fold mass difference between nanostar and excess amino acids (AA). Furthermore, the unimolecular, fully defined composition and UV chromophore are commensurate with reaction monitoring by liquid chromatography and mass spectrometry.

**Scheme 1:**
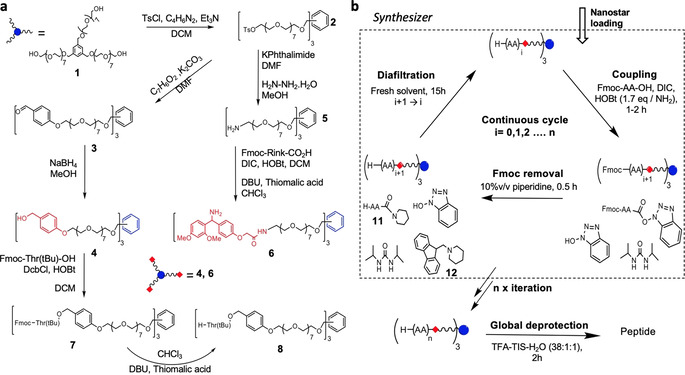
Synthesis of nanostars and chain extension cycle: a) Tris‐hydroxy terminated octagol‐nanostar **1** is converted to Wang‐type, **4**, and Rink, **6**, nanostar; the Wang anchor is loaded by slow esterification with the first AA before entering the synthesizer. b) Using the Fmoc strategy, peptide‐nanostars are grown in a three‐step cycle of coupling, Fmoc removal, and diafiltration, then removed from the synthesizer for global deprotection.

Nanostar anchor synthesis commenced from the known tris‐hydroxy terminated octagol‐nanostar **1**[^12a^, ^12b^] (Scheme 1 a). For compatibility with common peptide synthesis strategies the termini must be modified. Either hydroxymethyl benzyl groups, to mimic Wang resin, or the Rink amide linker were appended to the chain termini of the nanostar. The hydroxyls were first activated as their p‐toluene sulfonyl esters **2**, then displaced by p‐hydroxy benzaldehyde. The intermediate tris(benzaldehyde) nanostar **3** was then reduced cleanly with NaBH_4_ to provide the HO‐Wang‐nanostar **4**. Alternatively, the initial nanostar tosylate **2** was converted to the corresponding triamine via Gabriel synthesis (phthalimide, then hydrazinolysis, Scheme S2). The resulting tris(amino‐octagol)nanostar **5** was readied by amidation with Fmoc‐Rink‐CO_2_H linker in the presence of N,N′‐diisopropylcarbodiimide (DIC) and hydroxybenzotriazole (HOBt), then deprotection with 1,8‐diazabicycloundec‐7‐ene (DBU) and thiomalic acid (Scheme 1 a); the latter quenches dibenzofulvene (DBF) to form an adduct that is readily removed by aqueous washing.^[5b]^ The resulting H‐Rink‐nanostar **6** (MW=2120 Da) has a loading capacity of 1.42 mmol g^−1^ which is higher than the corresponding solid phase resin (H‐Rink amide ChemMatrix® ≈0.5 mmol g^−1^), or the 5 kDa PEG employed in MEPS (0.2 mmol g^−1^). HO‐Wang‐nanostar **4** (MW=1544 Da) has a loading capacity of 1.94 mmol g^−1^ which also compares favourably with commercial supports (HO‐Wang‐PS≈1.0 mmol g^−1^).^[14]^

H‐Rink‐nanostar **6** can be used directly in PEPSTAR because the arms terminate with amino groups compatible with standard peptide coupling chemistry. However, loading the first amino acid onto a Wang‐type anchor requires a slow esterification, so this reaction was performed at high concentration in a glass flask. We tested the esterification step with large and sterically hindered Fmoc‐Thr(tBu)‐OH.^[15]^ Fmoc‐Thr(tBu)‐OH was condensed overnight with **4** using DcbCl and HOBt in dichloromethane to give Fmoc‐Thr(tBu)‐O‐Wang‐nanostar **7**. Intermediate **7** was deprotected with DBU and thiomalic acid to give loaded anchor H‐Thr(tBu)‐O‐Wang‐nanostar **8**, ready for PEPSTAR.

**Figure 2:**
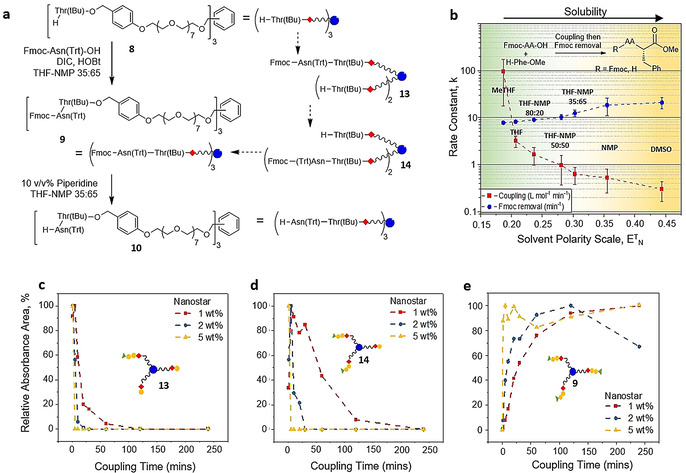
Variation in reaction rates for a dimer‐nanostar: a) The intermediates of peptide‐nanostar chain extension. b) The variation in the average rates of coupling to form Fmoc‐AA‐Phe‐OMe and of Fmoc removal with solvent polarity; tested with AA=Val, Leu, Glu(Trt), Asp(tBu), Trp(Boc), Arg(Pbf), Ala. As a general trend, solubility of H‐dipeptides increases with solvent polarity. c–e) Variation in concentration with time of 1‐arm **13** and 2‐arm **14** intermediates, and Fmoc‐dipeptide‐nanostar **9**, respectively, from different substrate concentrations; red 1 wt %, blue 2 wt % and yellow 5 wt % starting concentration of H‐Thr‐nanostar **8**. NB the drop in relative absorbance for 2 wt % after 125 mins in (e) was most likely caused by sample preparation errors, high dilution was necessary in order to quench the reaction prior to the UHPLC‐MS analysis.

**Synthetic strategy**. The widely used Fmoc‐peptide chemistry was adopted for PEPSTAR.^[16]^ In classical LPPS Fmoc‐peptide synthesis, four steps are typically required: Coupling, isolation, deprotection and isolation again. Aiming for a one‐pot synthesis strategy with sequential couplings and Fmoc removal, we elected to omit the post‐coupling purification step by quenching excess amino acid (AA) active ester. This has the advantages of reducing both solvent consumption and total cycle time. A sacrificial species is required to react with the active ester to prevent uncontrolled peptide chain extension after Fmoc removal. Initially, in the reaction of H‐Thr(tBu)‐O‐Wang‐nanostar **8** with Fmoc‐Asn(Trt)‐OH, aniline was tested for this purpose because of its low basicity and we believed it would give a UV‐active by‐product. Unfortunately, the reaction of aniline with active esters was too slow to afford effective quenching. However, despite dispensing with the active ester quench after deprotection of Fmoc‐Asn(Trt)‐Thr(tBu)‐O‐Wang‐nanostar **9** with piperidine, no double coupling contaminants could be detected in the product H‐Asn(Trt)‐Thr(tBu)‐O‐Wang‐nanostar **10** (Figure 2). We observed that remaining active ester was converted instantaneously by excess piperidine into H‐Asn(Trt)‐Pip **11** (Scheme 1 b).^[17]^

The reaction of excess AA with piperidine eliminated the need for both an additional sacrificial species and a separate diafiltration post‐coupling. Thus, a synthesis cycle requiring only one diafiltration step was devised, as illustrated in Scheme 1. After charging the synthesizer with nanostar, the chain extension cycle was initiated: The first Fmoc‐amino acid and condensing agent were injected into the synthesizer; after UHPLC‐MS confirmed the coupling was complete, Fmoc removal was initiated with piperidine; finally, after UHPLC‐MS confirmed complete deprotection, diafiltration commenced to remove the H‐AA‐Pip **11** derived from excess AA, the DBF‐Pip adduct **12**, and other reaction by‐products, while the H‐AA‐nanostar was rejected by the OSN membrane and remained in the synthesizer. This process was repeated for every new amino acid in the sequence up to H‐(AA)_*n*_‐nanostar, and resembles SPPS in its simplicity.

**Nanostar coupling and Fmoc removal kinetics**. Chain extending a peptide‐nanostar proceeds to completion via two intermediate species. Taking the formation of Fmoc‐dipeptide‐nanostar **9** as an example (Figure 2 a), condensing [H‐Thr(tBu)‐O ‐Wang]_3_‐nanostar **8** with Fmoc‐Asn(Trt)‐OH, the initial species formed has only one arm coupled, [Fmoc‐Asn(Trt)‐Thr(tBu)‐O‐Wang]‐[H‐Thr(tBu)‐O‐Wang]_2_‐nanostar (1‐arm, **13**). The 1‐arm intermediate is then consumed to give a second species with two arms coupled (2‐arm, **14**), but with one amino‐terminated arm still free. Finally, the 2‐arm intermediate reacts on the remaining amino group to give the desired Fmoc‐dipeptide‐nanostar **9**. Each of these species is chemically distinct and chromatographically resolvable. Before examining complex peptide‐nanostars, the variation in reaction rates for forming a range of dipeptides, and their subsequent Fmoc removal, was examined in various solvents (see SI section 4). Suspecting that a single solvent could not optimally fulfil all the required roles, binary mixtures of THF and NMP in different proportions were also considered.^[14]^ It was found that there is a strong correlation between solvent polarity and reaction rate; low polarity leading to fast coupling and slow deprotection, and vice versa in high polarity solvents (Figure 2 b). It was also noted that the urea by‐product (DIU) of AA activation with DIC and HOBt, and dipeptides after Fmoc removal, had poor solubility in low polarity solvents.^[18]^ Therefore, a THF‐NMP 35:65 v/v mixture was selected to provide a balance between reasonably fast reaction kinetics, for both coupling and Fmoc removal, and solubility, and was used for a study of the kinetics of peptide‐nanostar coupling and Fmoc removal.

Kinetic runs at three different concentrations of [H‐Thr(tBu)‐O‐Wang]_3_‐nanostar **8** (1, 2 and 5 wt %) were performed in a carousel reactor with THF‐NMP 35:65 v/v solvent (See SI section 5). Coupling reactions containing 1 wt % **8** and 5 equiv Fmoc‐Asn(Trt)‐OH (i.e. 1.7 equiv per amino terminated arm) went to completion smoothly: the 1‐arm intermediate formed almost instantaneously (Figure 2 c), but the time needed for the 2‐arm species to drop below both UHPLC and MS detection was over 4 hours (Figure 2 d). The coupling was also carried out at higher concentrations of nanostar **8** where the time for complete reaction to 3‐arm Fmoc‐dipeptide‐nanostar **9**, that is, for 2‐arm intermediate **14** to disappear, was reduced markedly (Figure 2 e): at 2 and 5 wt % the coupling was finished within one hour and 2 minutes, respectively. Upon addition of 10 v/v% piperidine, Fmoc removal was much faster than coupling, being complete <10 minutes (Table S3). These highlight the importance of nanostar concentration to reaction kinetics, defining the lower boundaries for nanostar concentration.

**Figure 3:**
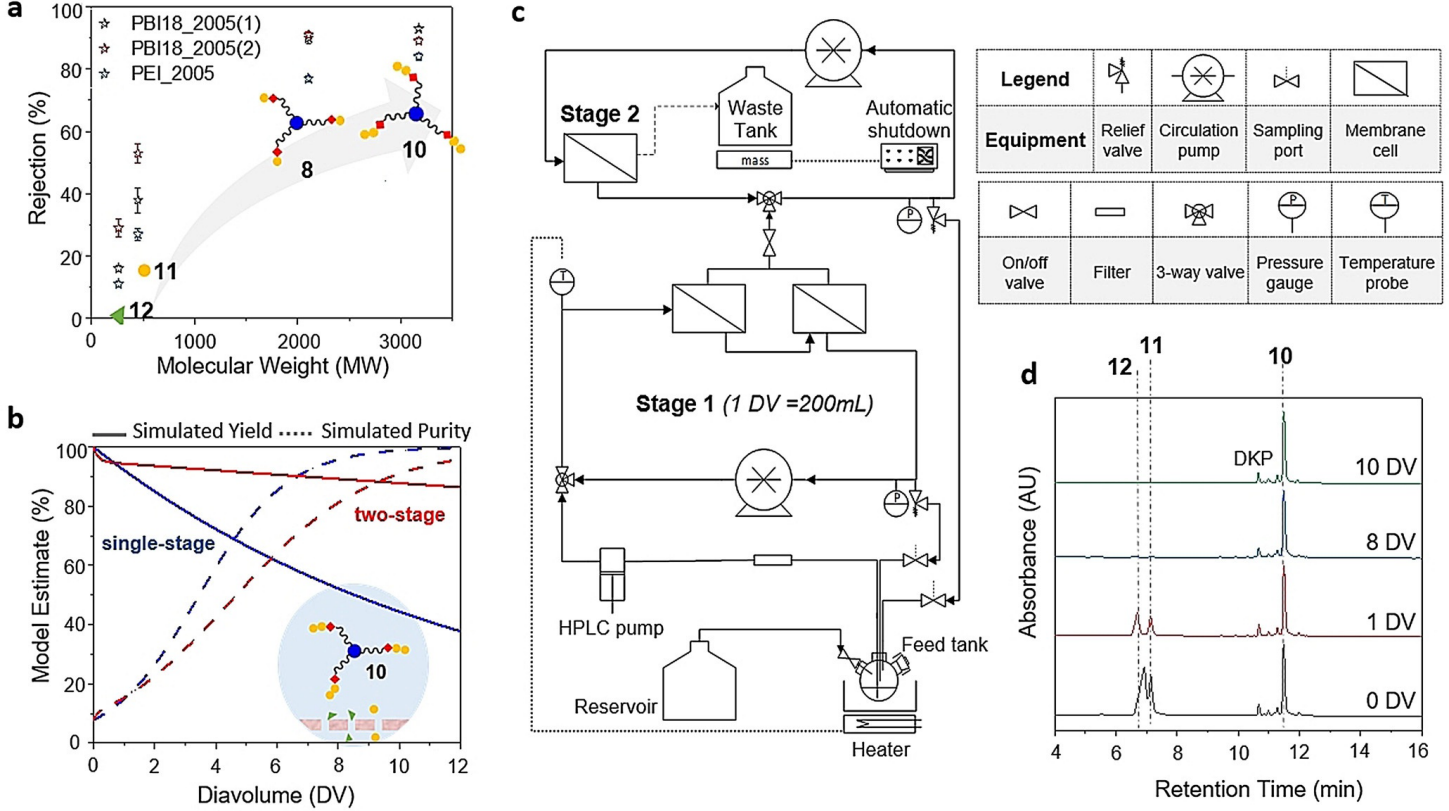
Synthesizer design and separation performance: a) Rejection of peptide‐nanostars **8** and **10**, and reaction by‐products **11** and **12**, by candidate membranes. b) Modelling the retention and purity of dipeptide‐nanostar **10** in single stage or two‐stage membrane separators containing PBI_2005(1); the early dip in the two‐stage yield curve is a result of redistribution of a small proportion of **10** from stage 1 to stage 2. Diavolume is the ratio of cumulative volume of wash solvent introduced to the synthesizer at any given time of diafiltration per volume of stage 1 (DV=Wash solvent volume / stage 1 volume), 1 DV=200 mL. c) Schematic of synthesizer layout, with picture of the synthesizer setup shown in Figure S8. d) Purification of H‐dipeptide‐nanostar **10** in the synthesizer, from the largest by‐products H‐Asn(Trt)‐Pip **11** and DBF‐Pip **12**; to the 2‐arm by‐product from diketopeparazine (DKP) can be detected, although this does not affect final peptide purity.

**Membrane selection and synthesizer design**. The OSN membrane is at the heart of PEPSTAR technology. Three chemically robust polymeric membranes developed in our laboratory, capable of permeating larger solutes, were screened for OSN: A polyethyleneimine (PEI) asymmetric membrane, cross‐linked with terephthalic chloride (TPC), and coated with Jeffamine® M‐2005 (a polyether mono‐amine), denoted PEI_2005; two different batches of polybenzimidazole (PBI) asymmetric membrane, cross‐linked with α,α′‐p‐dibromoxylene (DBX), and modified with a polymer brush Jeffamine® M‐2005, denoted PBI_2005(1) and PBI_2005(2).^[19]^ These surface‐modified membranes offer anti‐fouling properties to reduce the undesirable deposition of solutes on the surface.^[20]^ The key difference between PBI_2005(1) and PBI_2005(2) was that the latter was stored in acetonitrile for longer period prior to cross‐linking, allowing the freshly cast PBI film to tighten to varying extents. Membrane screening was conducted in THF which is a good solvent for peptide intermediates, and the rejections were calculated based on Equation (S3) (Figure 3 a, see also SI section 6). As expected from their MWs, H‐Asn(Trt)‐Pip **11** always had a higher rejection than DBF‐Pip **12**, therefore the critical separation to achieve was selective permeation of residual building block while retaining the nanostar. The efficiency for separating molecule A from B is expressed by the separation factor, β_A/B_ [Eq. (S4)] the higher it is the better the separation. PBI_2005(1) gave the highest separation factor β_**10**/**11**_ for purifying H‐dipeptide‐nanostar **10** from by‐product **11** (9.2) compared to both PBI_2005(2) (4.2) and PEI_2005 (4.7); the separation factor for smaller H‐AA‐nanostar **8** and by‐product **11**, β_**8**/**11**_, was again highest for PBI_2005(1) (6.1). Although PBI_2005(2) gave a similar peptide‐nanostar rejection to PBI_2005(1), the separation factor is much lower due to its simultaneously higher rejection of by‐product. Hence, PBI_2005(1) was selected for further studies.

Figure 3 a shows that the rejection rises with MW, but only approaches 100 % rejection slowly. Thus, even though PBI_2005(1) gave the highest separation factor, a significant loss of yield is inevitable during diafiltration, particularly during the early cycles of synthesis when the MW of the peptide‐nanostar is relatively low. For this reason, membrane “cascade” systems were conceived that used membranes linked in series to overcome the performance limits of a single membrane by recycling partial flows back to previous stages. Building on this concept, a practical two‐stage separator, requiring a single HPLC pump to pressurize the equipment, and using pressure relief valves to control the pressure in each stage, was implemented for the first time by Kim et al.^[21]^ Modelling, based on the measured rejection value for PBI_2005(1), predicted that if a purity of 90 % was required, then at least 40 % of dipeptide‐nanostar **10** would be lost to permeation using a single stage diafiltration system (Figure 3 b). However, if a two‐stage separation system was adopted using the same membranes, yield loss improved to about 10 % while achieving the same purity. Our synthesizer is based upon this two‐stage design, which is a compromise between achievable yield, complexity, and the time required for diafiltration (which rises with the number of stages).

The key difference between the synthesizer described here and earlier membrane separation apparatus^[12]^ is that the chemistry to assemble the heteropolymer, in this case a peptide, occurs inside the same equipment as is used to undertake purification of the crude product from reagent and by‐products. The principal elements of the synthesizer are (Figure 3 c): The feed‐tank, into which fresh solvent is passively added during OSN from a reservoir at the same rate as the waste stream from OSN drains to a collection tank (providing constant volume diafiltration); the filling of the waste tank is monitored electronically to enable automatic shut‐down. An HPLC pump takes liquid from the feed tank to pressurize the stage 1 circulation loop containing two membrane cells. The pressure is regulated by a pressure relief valve that returns liquid from the stage 1 loop back to the feed tank. The permeate from stage 1 enters the stage 2 circulation loop where the pressure is again controlled by a pressure relief valve. The stage 2 membrane concentrates the peptide‐nanostar permeating from stage 1 and a partial recycle flow returns to stage 1, increasing yield. Pumps in both stages circulate at 90 L h^−1^, to minimize concentration polarization at the membrane surface.

The synthesizer was validated with a synthesis run to form dipeptide‐nanostar **10**. The membrane cells of the synthesizer were fitted with disks of PBI_2005(1), and stage 1 was charged with H‐Thr(tBu)‐O‐Wang‐nanostar **8** in THF. On addition of DIC, HOBt and Fmoc‐Asn(Trt)‐OH to the reactor, the coupling proceeded smoothly to completion, as did the subsequent Fmoc removal upon addition of 10 %v/v piperidine. Once the chemistry was complete, the valve to stage 2 was opened commencing diafiltration (see SI section 7). After 10 DV of solvent had permeated H‐Asn(Trt)‐Thr(tBu)‐O‐Wang‐nanostar **10** was completely purified with by‐products below the detection limit (Figure 3 d). To test the general applicability of the method, we extended the synthesizer validation to different H‐AA‐Pip (see Figure S9). Results showed that 10 DV were sufficient to remove most H‐AA‐Pip. During optimization and several repeats of this protocol the membrane exhibited constant performance, and later visual inspection confirmed no signs of chemical or physical degradation.

**Figure 4:**
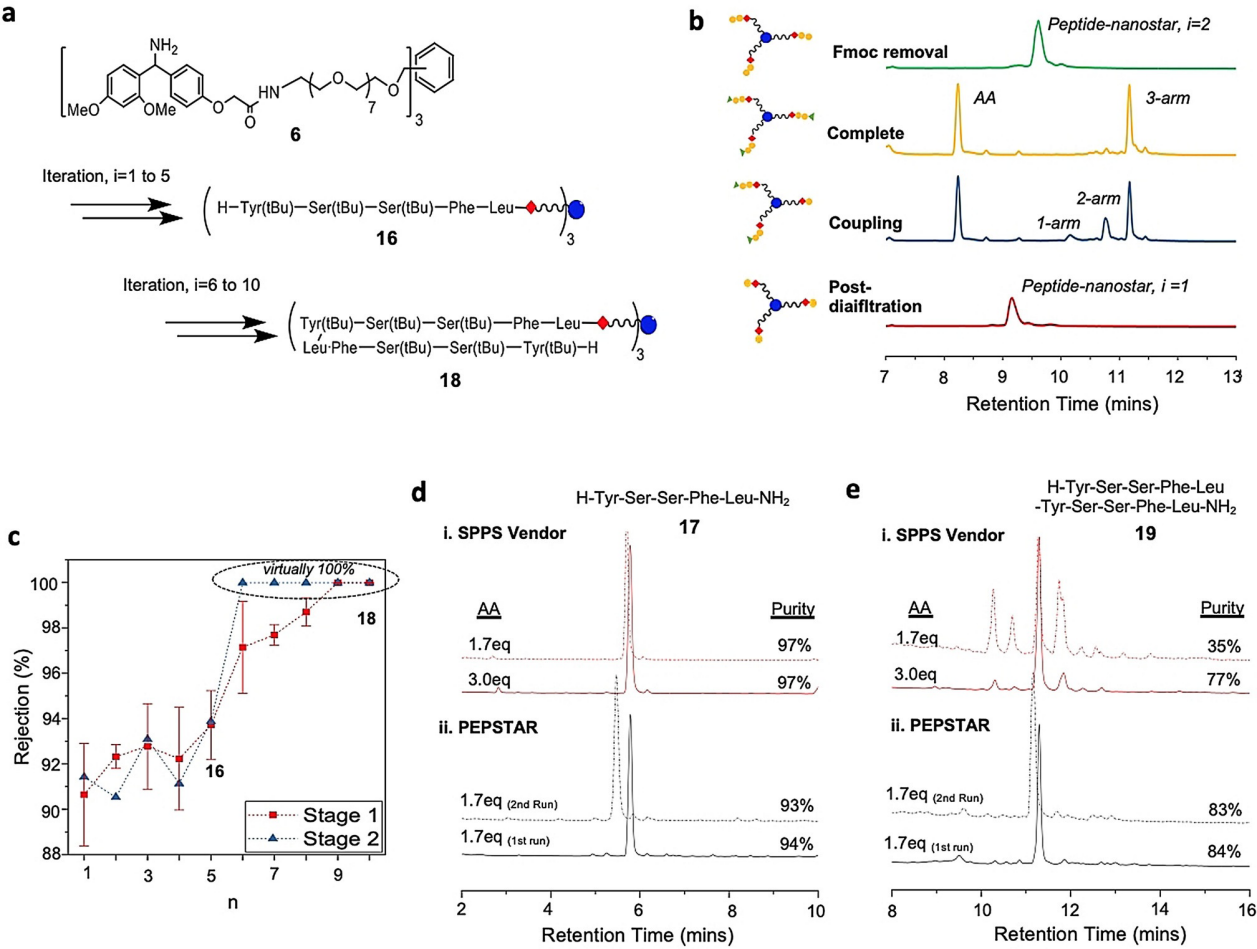
Comparison of the synthesis of model peptides by PEPSTAR and SPPS: a) Synthesis of enkephalin‐like model peptides. b) UHPLC chromatograms for the chain extension of H‐dipeptide‐nanostar, exhibiting the 1‐arm and 2‐arm chain extended intermediates, and Fmoc removal. c) Rise of H‐peptide‐nanostar rejection with peptide length; NB the error bars for stage 2 rejection (not shown) are large, due to low concentrations and corresponding variation in peak integrals above noise. d & e) Comparison of the crude purities of pentapeptide **17** and decapeptide **19** prepared by vendor SPPS or by PEPSTAR.

**Proof of concept**. With the kinetics optimized, it was necessary to define an initial target for preparing longer peptides that would assist in validating the operation of the new synthesizer. Although the HO‐Wang‐nanostar **4** is essential for preparing peptides with C‐terminal carboxylic acids, as with its counterparts among SPPS supports, at i=2 it suffers from low to moderate peptide cleavage from the support during extension, due to base catalyzed cyclization to form the diketopiperazine (DKP).^[22]^ Although for most sequences the yield loss is low (≈10 % as determined on chromatograms for example, Figure 3 d), it introduces additional signals into the subsequent UHPLC chromatograms, corresponding to (peptide‐O‐Wang)_2_‐(HO‐Wang)‐nanostar, that will confound the analysis of a new system. For this reason, we changed to the H‐Rink‐nanostar anchor, used for preparing C‐terminal amides, because it does not produce analogous DKP by‐products. To validate the process of repeated chain extension cycles during the development phase of PEPSTAR, an enkephalin‐like model peptide sequence was designed (Figure 4 a). The glycine residues of the native Leu‐enkephalin (H‐Tyr‐Gly‐Gly‐Phe‐Leu‐NH_2_) sequence were replaced by serine to make (Ser2,Ser3)‐Leu‐enkephalin pentapeptide (H‐Tyr‐Ser‐Ser‐Phe‐Leu‐NH_2_), because H‐Gly‐Pip displayed lower solubility in THF‐NMP 35:65 than other H‐AA‐Pip; this obstacle may be avoided by nanofiltering Fmoc‐Gly‐OH prior to deprotection via an additional diafiltration. Stage 1 of the synthesizer (total ≈200 mL) was charged with unloaded H‐Rink‐nanostar **6** (≈1.7 wt %) dissolved in THF‐NMP 35:65. The same protocols were then used to run the synthesizer, except that the temperature was maintained at 35 °C for all reactions via a heater feedback control loop. Fmoc‐Leu‐OH (5 equiv, that is, 1.7 equiv per amino terminated arm) in the minimum amount of solvent, preactivated for 1 minute with DIC and HOBt, was injected into stage 1 of the synthesizer via the feed tank. Once UHPLC‐MS confirmed completion of the coupling, piperidine (20 mL, 10 v/v%) was added to the feed tank to initiate Fmoc removal and quenching of the active ester; the piperidine was added slowly to further ensure that all the AA was trapped before Fmoc removal commenced. As before, diafiltration was initiated after reaction completion, but now in THF‐NMP. Once the H‐Leu‐Rink nanostar (i=1) had been purified, the synthesis cycle was repeated four more times to obtain H‐pentapeptide‐nanostar **16** (i=5, Figure 4 a).

All reactions and OSN were monitored in real‐time with UHPLC‐MS. As before, the 1‐arm and 2‐arm intermediates, as well as the 3‐arm desired peptide‐nanostar, are well resolved on the chromatogram (see Figure 4 b for i=1 to 2), permitting easy and sensitive reaction monitoring, and the same is true for subsequent Fmoc removal; the peak identities were corroborated by mass spectrometric characterization (Figure S10). Most couplings went to completion within 1 hour, but to minimize traces of deletion sequences the reactions were allowed to run for an additional hour. Much faster Fmoc deprotection was largely complete within 10 mins, but was allowed to run for 30 mins, again to minimize chain length errors. Monitoring of purification showed that (e.g. Figure 3 d) after 8 *diavolumes* (1 DV≈200 mL) had permeated all large MW reaction by‐products were removed, including H‐AA‐Pip and DBF‐Pip. However, purifications were extended to 10 DV to reduce residual piperidine to below 0.1 v/v% (as determined gas chromatography); this is essential to prevent decomposition of fresh Fmoc‐AA‐OH added at the start of the next cycle. As the peptide‐nanostar grew in length, its rejection also increased and reached virtually 100 % from i=8 onwards (Figure 4 c). Diafiltration in our small synthesizer typically required 15 hours, due to the relatively low membrane area/volume ratio of flat sheet cells. Thus, automatic shutdown ensured that one cycle could be undertaken reliably every 24 hours by running most of the OSN purification overnight. Although OSN was slower in THF‐NMP than THF alone, due to the higher viscosity of NMP, the separation was very efficient, possibly enhanced by the bulky Rink amide moiety.

Global deprotection of H‐pentapeptide‐nanostar **16** was undertaken in TFA‐TIS‐H_2_O 38:1:1 (Scheme 1 b), after which the crude pentapeptide H‐Tyr‐Ser‐Ser‐Phe‐Leu‐NH_2_ **17** exhibited 94 % purity (Figure 4 d). This level of purity, using only 1.7 equiv Fmoc‐AA‐OH per arm every cycle, is comparable with SPPS vendor using 3.0 equiv AA to obtain 97 % crude purity. Encouraged by this success, the H‐pentapeptide‐nanostar **16** was further elongated to a decapeptide by repeating the model (Ser2,Ser3)‐Leu‐enkephalin sequence (Figure 4 a). The same PEPSTAR protocols were applied, with UHPLC‐MS monitoring of reactions and OSN. At i=9 the nonapeptide‐nanostar UHPLC peak shape became broader and mis‐shaped, with lower MS sensitivity (although reaction progress could still be determined by changes in retention time), probably due to poor solubility of the hydrophobic peptide‐nanostar in the part‐aqueous mobile phase (Figure S10). This time global deprotection of decapeptide‐nanostar **18**, afforded crude decapeptide H‐(Tyr‐Ser‐Ser‐Phe‐Leu‐)_2_‐NH_2_ **19** with 84 % purity from 1.7 equiv, considerably improving on the crude purity of 77 % from SPPS vendor, again 3.0 equiv AA per cycle (Figure 4 e). The decapeptide yield was 75 %. Although ≈5 % of the loss can be accounted for due to frequent sampling from the synthesizer, the majority was lost in the first five cycles, particularly the first, when the peptide‐nanostar rejection was relatively low.

To investigate the effects of AA equivalents on SPPS, the excess of AA per cycle was reduced from 3.0 equiv to 1.7 equiv, similar to that used in PEPSTAR protocols. Although the pentapeptide **17** purity was unaffected by the reduced excess of AA, the crude purity of SPPS decapeptide **19** plummeted from 77 % to 35 %. The impurities were identified to be deletion sequences, presumably caused by on‐resin peptide aggregation or conformational effects during the elongation from i=5 to 10 which led to incomplete coupling or Fmoc removal (Figure S12). However, the authors emphasize that the solid phase synthesis with reduced excess of AA was not optimized to achieve the highest purities, but to mirror PEPSTAR quantities of reagents (see SI section 11).

To demonstrate the reliability of PEPSTAR, the model penta‐ and decapeptide syntheses were reproduced giving **17** and **19** of essentially the same quality and yield (both +/‐ 1 %), (Figure 4 d,e(ii). Two additional runs were performed, but with the initial H‐Rink‐ nanostar **6** at 1 and 3 wt %, to evaluate the effect of concentration on the performance of the synthesizer. Under identical conditions to those above, the 1 wt % Rink anchor solution resulted in lower purities of penta‐ and decapeptides **17** and **19** at values of 87 % and 60 %, respectively; this is expected from slower kinetics as a result of higher dilution. At 3 wt % initial nanostar concentration, the kinetics were fast and clean up to the pentapeptide, but at the Fmoc‐octapeptide‐nanostar, where the supported peptide concentration was ≈9 wt %, plus a further 3 wt % reagents, gelation of the reaction solution occurred. As expected, any LPPS process has a solubility ceiling that varies from one target peptide to the next, but should be amenable to further optimization of solvents, temperature and protecting groups. For the reported model peptide, the optimum starting concentration for H‐Rink‐nanostar **6** is around 2 wt %, corresponding to 7 wt % of final H‐decapeptide‐nanostar **18**.

**Figure 5:**
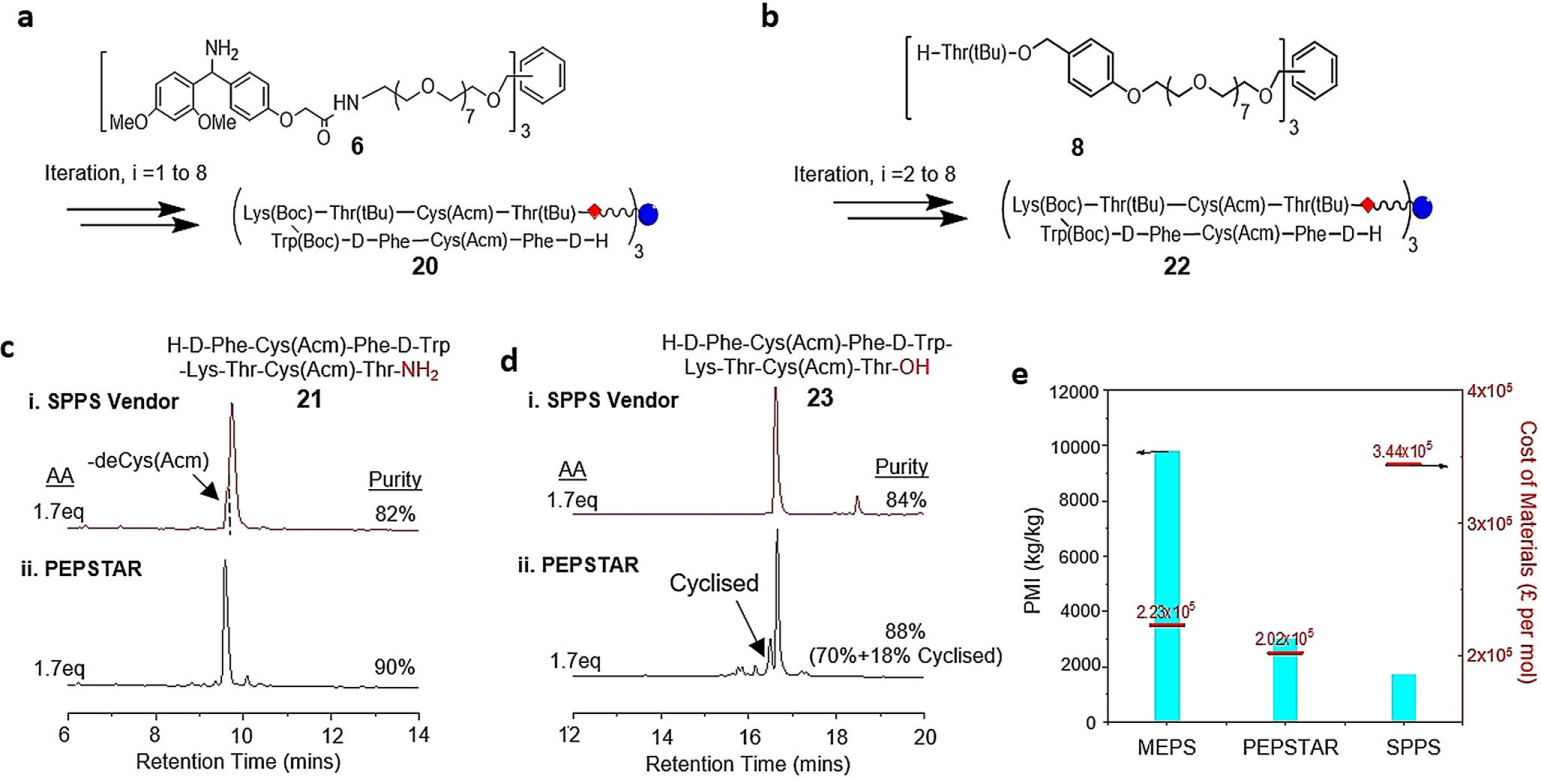
Comparison of the synthesis of linear octreotate by PEPSTAR: a & b) Synthesis of linear octreotate amide and acid. c & d) Comparison of the crude purities of SPPS vendor peptides with crude octreotate amide **21** and octeotate acid **23**, respectively, prepared by PEPSTAR. e) Comparison of Process Mass Intensity (PMI) and costs of materials for different methods of synthesis for octreotate amide **21**.

**Octreotate**. After validation of the PEPSTAR synthesis system, we selected linear octreotate amide as a more typical peptide target. Starting with an H‐Rink‐nanostar **6** concentration of around 2 wt %, the octapeptide sequence was synthesized using the same protocols as above in THF‐NMP 35:65. Notably, the Fmoc‐Cys(Acm)‐OH building block was selected, aiming for Cys to withstand the acidic global deprotection with the Acm intact. All reactions and diafiltrations went smoothly with no solubility issues. The fully protected peptide‐nanostar **20** was obtained in 80 % yield, and after global deprotection the crude purity of linear octreotate amide **21** was 90 % (Figure 5 c). During the synthesis towards peptide‐nanostar **20**, the degree of epimerization was also investigated at the epimerization‐prone Cys residue. The global deprotection of H‐Thr(tBu)‐Cys(Acm)‐Thr(tBu)‐Rink‐nanostar (i=3) showed <0.1 % of epimerization from L‐ to D‐Cys (Figure S14).

To further explore the capability of PEPSTAR, the synthesis was recapitulated with H‐Thr(tBu)‐O‐Wang‐nanostar **8** to produce linear octreotate with C‐terminal carboxylic acid. To minimize the losses due to base‐catalyzed DKP formation, extension cycle and subsequent Fmoc removal from i=1 to 2 were conducted in just the feed tank, setting the nanostar and piperidine concentrations at ca. 10 wt % and 10 v/v%, respectively. Upon completion, the reaction mixture was immediately diluted into stage 1, lowering the nanostar and piperidine concentrations to 2 wt % and 2 v/v%. Diafiltration was then initiated, reducing the piperidine concentration in stage 1, thus minimizing the time that piperidine would have a high enough concentration to catalyze loss of dipeptide to DKP. Even so, 10 % (H‐dipeptide‐O‐Wang)_3_‐nanostar was still converted to (H‐dipeptide‐O‐Wang)_2_‐(HO‐Wang)‐nanostar via DKP. From i=2 onwards, the standard protocol was applied. All reactions and diafiltrations went smoothly to obtain fully protected peptide‐nanostar **22** in 71 % yield. After global deprotection the purity of linear octreotate was 70 %, 88 % if you include 18 % cyclized octreotate from loss of Acm, which is also comparable to SPPS (Figure 5 d, see also Figure S16). The fast reaction kinetic facilitated in liquid phase was suspected to cause Acm removal and promote cyclization. Hence, further optimizations are required for the global deprotection protocol.

With successful completion of the proof of concept synthesis of octreotate amide and acid, it is pertinent to ask how the environmental and economic credentials of a putative commercial PEPSTAR system compare at the first pass with MEPS and its principal competitor SPPS (Figure 5 e). Process Mass Intensity (PMI) is used to express solvent efficiency, which measures the total material used by mass per unit mass of peptide (kg kg^−1^).^[23]^ The PMI evaluation shows that, for the three methods under assessment, the solvent consumption contributed to nearly all the total waste generated with only a small fraction contributed by hubs (or resins) and other reagents (see Table S6). The PMI is drastically lower for PEPSTAR than for MEPS, and only slightly higher than for SPPS. To compare production costs, we summed up the costs of materials for PEPSTAR and MEPS to produce 1 mole of linear octreotate amide based on current supplier prices (*Sigma–Aldrich*). Using the same material prices, we then estimated the cost for the vendor of SPPS to produce similar quantity of product following the standard protocol (see SI section 11). The cost of materials for SPPS are the highest due to the large excess of AA (3 equiv) required to achieve the specified purity (see Table S7). Furthermore, we anticipate that the PMI and cost of PEPSTAR will fall below the benchmark set by SPPS once downstream purification steps are considered. The higher crude purity offered by liquid phase synthesis in turn requires less demanding chromatographic purification, which is often a major contributor to waste generation.^[24]^

## Conclusion

In this report, we have demonstrated liquid phase peptide synthesis via one‐pot nanostar sieving (PEPSTAR), a continuous process to synthesize high purity peptides, without phase or material transfers, suitable for automation. This advance depended on the development of highly stable, polymeric organic solvent nanofiltration (OSN) membranes that enabled reaction by‐products to be efficiently “sieved” out from the crude reaction mixture to fully purify the growing peptide by diafiltration. Equally, the design of a three‐armed star‐shaped monodisperse synthesis support, or nanostar anchor, for growing peptides facilitated efficient membrane separation and real time monitoring, of both the synthesis and purification phases of PEPSTAR. This one‐pot synthesis strategy lowered the PMI three‐fold from its LPPS predecessor, membrane‐enhanced peptide synthesis (MEPS), and is close to that of SPPS. The cost of materials is estimated to be half of SPPS's. This augurs well for manufacturing scale‐up of PEPSTAR which, because of its entirely liquid phase nature, is far less scale‐limited than SPPS, is expected to reduce the lengthy lead‐times for process development, and the PMI and production cost are anticipated to fall further at larger scales. This technology is highly flexible in terms of solvent and nanostar support choice. In future, full process automation and further innovations on nanostar support and solvent system will realize efficient synthesis of large peptides or even proteins on PEPSTAR.

## Conflict of interest

The authors declare no conflict of interest.

## Supporting information

As a service to our authors and readers, this journal provides supporting information supplied by the authors. Such materials are peer reviewed and may be re‐organized for online delivery, but are not copy‐edited or typeset. Technical support issues arising from supporting information (other than missing files) should be addressed to the authors.

Supplementary
