# Supplementary material for: Liquid Phase Peptide Synthesis via One‐Pot Nanostar Sieving (PEPSTAR)
Source: Angew Chem Int Ed Engl. 2021 Feb 24;60(14):7786–95. doi: 10.1002/anie.202014445 (PMC8049079; doi:10.1002/anie.202014445)

Angewandte Chemie
Eine Zeitschrift der Gesellschaft Deutscher Chemiker

Supporting Information

Liquid Phase Peptide Synthesis via One-Pot Nanostar Sieving (PEPSTAR)

*Jet Yeo, Ludmila Peeva, Seoyeon Chung, Piers Gaffney, Daeok Kim, Carla Luciani, Sergey Tsukanov, Kevin Seibert, Michael Kopach, Fernando Albericio, and Andrew Livingston**

anie_202014445_sm_miscellaneous_information.pdf

Table of Contents

Table of Contents 2
1. Materials 3
2. Characterizations and measurements 4
3. Nanostar design and synthesis 5
4. Variation of reaction kinetics with solvent 9
5. Nanostar coupling and Fmoc removal kinetics study. 12
6. Membrane fabrication and performance 14
7. Synthesizer operation 16
8. Modelling and simulation (single-stage vs two-stage) 18
9. Enkephalin-like model peptide synthesis 19
10. Octreotate amide and acid synthesis 22
11. Solid phase synthesis protocol 28
12. Process Mass Intensity (PMI) and cost of materials 29
13. References 30
Author Contributions 30

1. Materials

All chemicals used were purchased from the following commercial suppliers.

All reagents were used without further purification: Triethylamine (≥ 99.5%), N-methylimidazole (NMI, Reagent Plus®, ≥ 99%), 1,3-dihydro-1,3-dioxoisoindole potassium salt (potassium phthalimide, ≥98%), ammonium acetate (≥98%), 64-65% hydrazine monohydrate (≥98%), N,N'-diisopropylcarbodiimide (DIC, ≥98%), hydroxybenzotriazole (HOBt, ≥97%), 1,8-diazabicyclo[5.4.0]undec-7-ene (DBU, ≥99%), mercaptosuccinic acid (thiomalic acid, ≥97%), potassium acetate (≥98%), piperidine (ReagentPlus®, ≥99%), trifluroacetic acid (TFA, ReagentPlus®, ≥99%), triisopropylsilane (TIS, ≥98%), p-toluenesulfonylchloride (ReagentPlus®, ≥99%), 2,6-dichlorobenzoyl chloride (DcbCl, 99%), pyridine (99.8%) from *Sigma-Aldrich*. 4-hydroxybenzyaldehyde (>98%) from *TCI*. Sodium hydrogen carbonate (≥99%), sodium hydroxide (≥99%, VWR) and hydrochloric acid (37%) from *VWR Chemicals*. 4-[(2,4-dimethoxyphenyl)(Fmoc-amino)methyl]-phenoxyacetic acid (Fmoc-Rink linker) (≥98%) from *Novabiochem*®.

Solvents were used without further purification, except where specified: HPLC-grade water (≥99.9%), deionized water from the laboratory, HPLC-grade acetonitrile (≥99.9%), dicholoromethane (DCM), acetonitrile (MeCN), N,N-dimethylformamide (DMF), chloroform, ethyl acetate, isopropanol (IPA), methanol, 2-methyltetrahydrofuran (MeTHF), tetrahydrofuran (THF), N-methyl-2-pyrrolidone (NMP), dimethyl sulfoxide (DMSO) were from *VWR Chemicals*. MeCN and DCM were dried over baked 4Å molecular sieves prior to use in anhydrous reactions.

Amino acid buidling blocks were used as received: L-Phenylalanine methyl ester hydrochloride (H-Phe-OMe) (98%), Fmoc-Gln(Trt)-OH (≥98%), Fmoc-Leu-OH (98%), Fmoc-Val-OH (98%), Fmoc-Phe-OH (98%), Fmoc-Ala-OH (95%), Fmoc-Gly-OH (98%), Fmoc-Thr(tBu)-OH (97%), Fmoc-Ser(tBu)-OH (97%), Fmoc-Tyr(tBu)-OH (98%), Fmoc-His(Trt)-OH (≥98%), Fmoc-Met-OH (≥98%), Fmoc-Asp(tBu)-OH (≥98%), Fmoc-Asn(Trt)-OH (≥97%), Fmoc-Trp(Boc)-OH (≥97%), Fmoc-Lys(Boc)-OH (≥98%), Fmoc-Arg(Pbf)-OH (98%), Fmoc-Cys(Acm)-OH (≥95%), Fmoc-D-Trp(Boc)-OH (≥95%), Fmoc-D-Phe-OH (≥98%) were from *Sigma-Aldrich*.

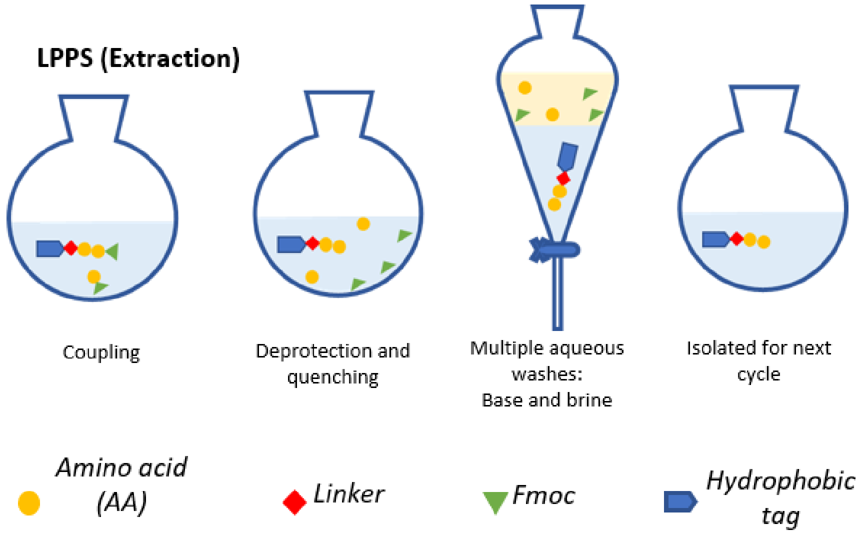

Figure S1: Liquid phase peptide synthesis via extraction.

2. Characterizations and measurements

Ultra-High Performance Liquid Chromatography with Mass Spectrometry (UHPLC-MS). Analysis was performed on an Agilent 1260 Infinity ll UHPLC system equipped with a UV-visible diode array variable wavelength detector (DAD), an evaporative light scattering detector (ELSD), and coupled to Agilent 6130 single quadrupole mass spectrometer (MS). An Acquity UPLC Protein BEH C4 column, 300 Å, 1.7 μm (2.1 mm x 100 mm) was used for separation. Mobile phase for UHPLC-M was a binary system: Solvent A, deionized water buffered with 5 μM ammonium acetate; Solvent B, acetonitrile.

The following gradients were developed for analysis:

Gradient 1 eluted with 10% solvent B for 1 min, followed by a linear gradient of 10% to 100% solvent B over 10 min, maintained at 100% solvent B for 3 min, and finally back to 10% solvent B with a linear gradient over 2 min.
Gradient 2 eluted with 10% solvent B for 1 min, followed by a linear gradient of 10% to 90% solvent B over 7 min, and back to 10% solvent B with a linear gradient over 1 min.
Gradient 3 eluted with 10% solvent B for 1 min, followed by a linear gradient of 10% to 100% solvent B over 40 min, maintained at 100% solvent B for 5 min, and finally back to 10% solvent B with a linear gradient over 2 min.
Gradient 4 eluted with 10% solvent B for 10 min, followed by a linear gradient of 10% to 100% solvent B over 30 min, maintained at 100% solvent B for 5 min, and finally back to 10% solvent B with a linear gradient over 2 min.

The pump flow rate was set to 0.3 ml/min, heating the column at 60 °C. The eluent was monitored at 210 nm, 220 nm and 280 nm.The ELSD was set to a gas flow rate of 1.6 SLM, with nebulizer temperature of 55 °C and evaporator temperature of 45 °C. MS was set to a gas flow rate of 8.0 l/min, nebulizer pressure at 35 psig, gas temperature of 350 °C and capillary voltage of 4000 V. Both positive and negative ion modes were obtained from electrospray ionization. *ChemStation* was used to process the data. *Gradient 1* was used for reaction monitoring of enkephalin-like model peptides and during linear octreotide amide syntheses. *Gradient 2* was used for monitoring coupling and deprotection of dipeptides during solvent screening. *Gradient 3 & 4* were used for the purity analyses of crude peptides.

Nuclear Magnetic Resonance (NMR). ^{1}H NMR and ^{13}C NMR spectra were recorded on a Bruker AVANCE III-400 NMR spectrometer, at 400 (^{1}H) and 101 (^{13}C) MHz frequencies with deuterated chloroform ($CDCl_3$) as solvents.

Gas Chromatography (GC). Analyses were performed on an Agilent gas chromatography apparatus with a flame ionization detector (GC-FIC 6850) and fitted with a HP-5 column (19095J-323E, Agilent). Column temperature was ramped at 15°C min^{-1} from 40 to 180°C.

High performance liquid chromatography (HPLC). The analysis was performed on 1100 Agilent HPLC system equipped with a UV-visible diode array varibal wavelength detector (DAD). A reversed-phase C18 column (250 mm × 4.6 mm, ACE Hichrom) for separation. The mobile phase for HPLC was a binary system comprised of deionized water buffered with 5mM ammonium acetate (Solvent A) and MeCN (Solvent B). Following gradient was developed for analysis. The samples were eluted with 0% solvent B over 5 minutes, and then a linear gradient to 100% solvent B over 15 min, and finally equilibrating the column back to 0% solvent B over 5 min. The HPLC pump flow rate was set at 1 mL/min, and the column temperature was kept at 60°C.The eluent was monitored at 220 nm.

3. Nanostar design and synthesis

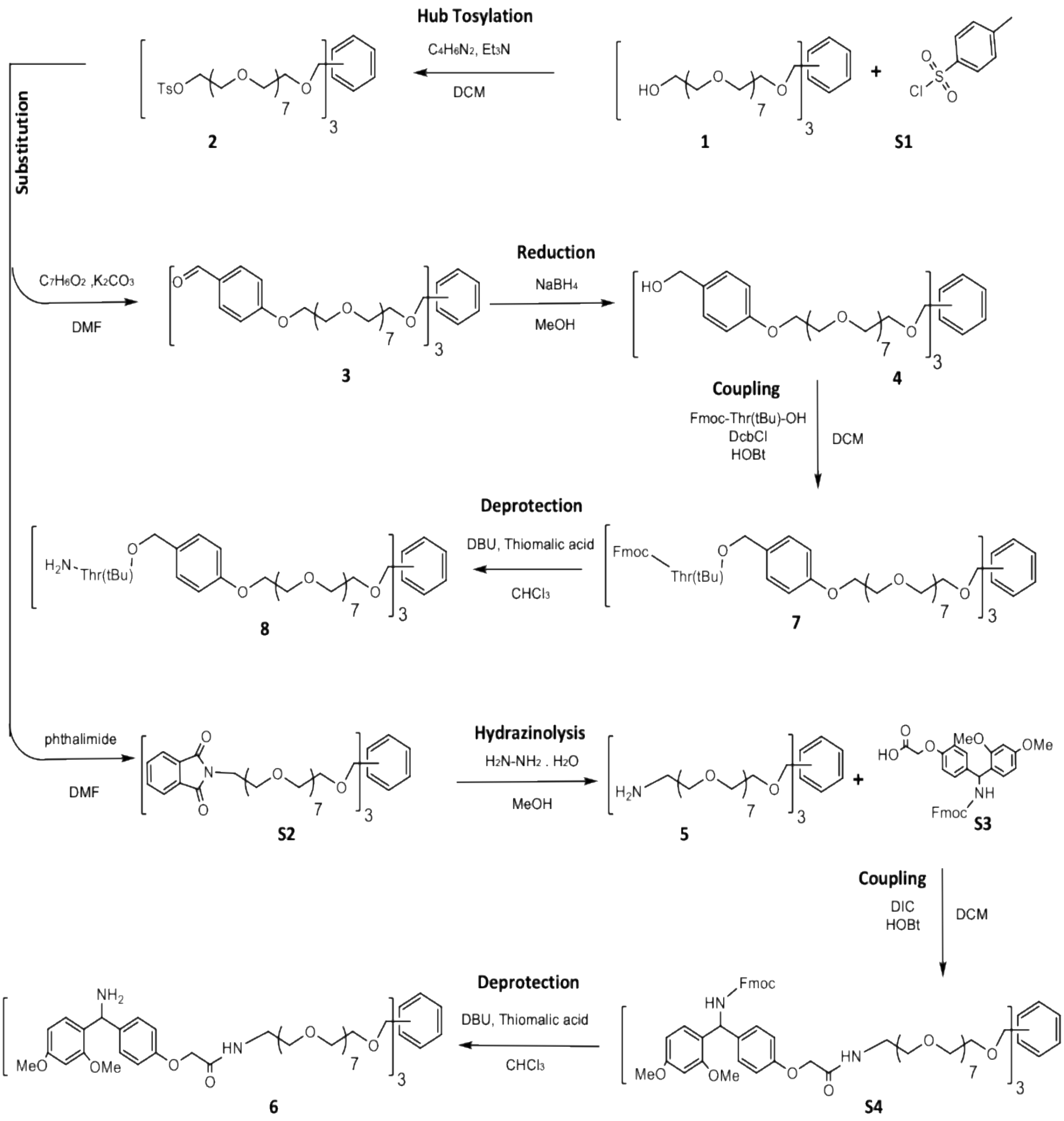

Scheme S2: The synthesis route for nanostar supports.

All reactions were conducted in anhydrous solvent under argon, unless otherwise specified.

1,3,5-Tris[p-toluenesulfonyl-octa(ethyleneglycol)oxymethyl]benzene, 2. 1,3,5-Tris[octa(ethyleneglycol)oxymethyl]benzene **1** (20.20 g, 16.5 mmol) was co-evaporated from dry MeCN (3×100 mL) before re-dissolving in DCM (200 mL). Triethylamine (20.8 mL, 148.4mmol, 9 eq), NMI (1.3, 16.5 mmol, 1eq) and then TsCl (15.7 g, 82.1 mmol, 5 eq) were added to the solution. The reaction was stirred at room temp. for 1 hour; completion was confirmed by UHPLC-MS (*Gradient 1*). To the solution was added THF (200 mL), and the DCM was then gently stripped off in a rotary evaporator with the water bath temperature maintained at 30°C until the vapour pressure dropped to < 0.1 bar. The residue was filtered through a glass sinter, washing the remaining solids with further THF (3×40 mL). To the filtrate was added $NaHCO_3$ (13.9 g, 165 mmol, 10eq) then water (100 mL) and the mixture was stirred at room temp. for 2 hours; under these conditions residual TsCl hydrolyzes completely. THF was then removed under reduced pressure and the residual aqueous phase was extracted with $CHCl_3$. The organic phase was collected and washed with potassium acetate buffer (pH 4.5), followed by a back-extraction with further $CHCl_3$. The organic phase was dried over Na_2SO_4 and the solvent was removed under reduced pressure to afford the title compound **2** (26 g, 94%), which was used without further purification.

^1H NMR (400 MHz, $CDCl_3$) – Appendix 1a

δ=7.80 (d, J=8.3 Hz, 6H, Ts, C**H**), 7.35 (d, J=8.1 Hz, 6H, Ts, C**H**), 7.24 (s, 3H, Hub C**H**), 4.55 (s, 6H, Hub-C**H₂**O), 4.16 (t, 6H, C**H₂**OTs), 3.75-3.56 (m, 90, PEG C**H₂**O), 2.45 (s, 9H, Ts C**H₃**) ppm.

^{13}C NMR (101 MHz, $CDCl_3$) – Appendix 1b
δ= 144.77 (3 C, Ts **C**SO_2), 138.60 (3C, Ts **C**CH_3), 133.05 (3C, Hub **C**), 129.81 (6C, Ts **C**H), 127.97 (6C, Ts **C**H), 126.29 (3C, Hub **C**H), 73.08 (3C, Hub **C**H_2O), 70.55-70.73 (m, 39C, PEG **C**H_2O), 69.55 (3C, Hub-CH_2O**C**H_2), 69.24 (3C, O**C**H_2CH_2OTs), 68.67 (3C, OCH_2**C**H_2OTs), 21.6 (3C, Ts **C**H_3) ppm.

MS (ESI+): calcd for $C_{78}H_{126}O_{33}S_3$+2.NH_4^{2+}, [*M*+2.NH_4]$^{2+}$ = 861.4; found *m/z* 861.4 (100%). [*M*+NH_4]$^+$ = 1704.5 (5%). [*M*+3.NH_4]$^{3+}$ = 580.5 (60%).

1,3,5-Tris[p-hydroxymethylphenoxy-octa(ethyleneglycol)oxymethyl]benzene / [H-Wang-O-Nanostar], 4. Nanostar tris-tosylate **2** (5.35 g, 3.17 mmol) was dissolved in DMF (50 mL), to which 4-hydroxybenzaldehyde (1.55 g, 12.69 mmol, 4 eq) then potassium carbonate (1.75 g, 12.69 mmol, 4 eq) were added, and the stirred mixture was heated in an oil bath at 110 °C for 1 hour. The reaction was followed closely by UHPLC-MS (*Gradient 1*) and stopped immediately upon completion to avoid side-reactions of the desired product with excess reagent. After cooling to room temp., DCM (50 mL) was added to the mixture which was filtered through a glass sinter, washing the solids with further DCM. The filtrate was evaporated to afford crude tris(benzaldehyde)-nanostar **3** (5.98 g).

Crude tris(benzaldehyde)-nanostar **3** (5.98 g) was dissolved in MeOH (120 mL). Solid sodium borohydride (0.88 g, 23.34 mmol, 6 eq) was added slowly to the vigorously stirred solution at room temp. and the reaction was complete after 1 hour, as confirmed by UHPLC-MS (*Gradient 1*). Deionized water (200 mL) was added to the mixture and the MeOH was stripped off at reduced pressure. An equal volume of saturated brine was added to the residual aqueous phase, which was extracted with $CHCl_3$. The organic phase was dried over Na_2SO_4 and evaporated to dryness to afford the title compound **4** (4.59 g, 94%), which was used without further purification.

^{1}H NMR (400 MHz, $CDCl_3$) – Appendix 2a
δ=7.29 (d, J=8.5 Hz, 6H, Wang C**H**), 7.24 (s, 3H, Hub C**H**), 6.91 (d, J=8.6 Hz, 6H, Wang C**H**), 4.61 (s, 6H, Hub-C**H₂**O), 4.55 (s, 6H, Wang-C**H₂**OH), 4.13 (dd, J=5.7, 4.0 Hz, 6H, CH_2C**H₂**O-Wang), 3.86 (dd, J=5.8, 4.0 Hz, 6H, C**H₂**CH_2O-Wang), 3.75-3.57 (m, 84H, PEG C**H₂**O), 2.15 (s, 3H, O**H**) ppm.

^{13}C NMR (101 MHz, $CDCl_3$) – Appendix 2b
δ=158.24 (3C, Wang CH_2O**C**), 138.55 (3C, Hub **C**), 133.49 (3C, Wang **C**CH_2OH), 128.56 (6C, Wang **C**H), 126.38 (3C, Hub **C**H), 114.63 (6C, Wang **C**H), 73.07 (3C, Hub-**C**H_2O), 70.82 (3C, Hub-CH_2O**C**H_2), 70.54 (s, 39C, PEG **C**H_2O), 69.74 (3C, **C**H_2CH_2O-Wang), 69.51(3C, **C**H_2O-Wang), 64.86 (3C, Wang-**C**H_2OH) ppm.

MS (ESI+): calcd for $C_{78}H_{126}O_{30}$+2.NH_4, [*M*+2.NH_4]$^{2+}$ = 789.5; found *m/z* 789.5 (100%). [*M*+NH_4]$^+$ = *1560.7* (5%). [*M*+3.NH_4]$^{3+}$ = 532.5 (30%).

1,3,5-Tris[amino-octa(ethyleneglycol)oxymethyl]benzene, 5. Nanostar tris-tosylate **2** (25.80 g, 15.30 mmol) was dissolved in DMF (200 mL) to which potassium phthalimide (16.98 g, 91.78mmol, 6 eq) was added. The mixture was stirred overnight at 70 °C; completion was confirmed by UHPLC-MS (*gradient 1*). The following day, the DMF was evaporated (rotary evaporator, 70 °C, then high vacuum) and the residue was re-dissolved in EtOAc (200 mL). The solids were filtered off in a glass sinter, washing with further EtOAc, and the filtrate evaporated to dryness. The residue was fractionated through a short column of flash silica, eluting with a gradient of MeOH-$CHCl_3$ (0:100 to 10:90 v/v), the product eluting in 4-5% MeOH. The appropriate fractions were evaporated to afford the intermediate tris(phthalamido) nanostar **S1** (23.00 g, 93%).

^{1}H NMR (400 MHz, $CDCl_3$) – Appendix 3a
δ=7.82 (dd, J=5.4, 3.1 Hz, 6H, phthalimide C**H**), 7.69 (dd, J=5.5, 3.1 Hz, 6H, phthalimide C**H**), 7.20 (s, 3H, Hub C**H**), 4.52 (s, 6H, Hub-C**H₂**O), 3.87 (t, J=5.8 Hz, 6H, CH_2C**H₂**-phthalimide), 3.71 (t, J=5.8 Hz, 6H, C**H₂**CH_2-phthalimide), 3.65-3.55 (m, 84H, PEG C**H₂**O) ppm.

^{13}C NMR (101 MHz, $CDCl_3$) – Appendix 3b
δ=168.38 (6C, phthalimide N**C**O), 138.54 (3C, Hub **C**), 133.86 (6C, phthalimide **C**H), 132.12 (6C, phthalimide **C**), 126.26 (3C, Hub **C**H), 123.09 (6C, phthalimide **C**H), 73.16 (3C, Hub-**C**H_2O), 70.75-70.45 (m, 36C, PEG **C**H_2O), 70.11 (3C, **C**$H_2OCH_2CH_2$-phthalimide), 69.58 (3C, Hub-CH_2O**C**H_2), 67.98 (3C, O**C**H_2CH_2-phthalimide), 37.31 (3C, **C**H_2-phthalimide) ppm.

MS (ESI+): calcd for $C_{81}H_{117}N_3O_{30}$+2.NH_4, [*M*+2.NH_4]$^{2+}$ = 823.9; found *m/z* 824.0 (100%). [*M*+NH_4]$^+$ = 1629.6 (5%). [*M*+3.NH_4]$^{3+}$ = 555.5 (45%).

Pthalamide nanostar **S2** (20.58 g, 12.77 mmol) was re-dissolved in MeOH (100 mL) and hydrazine hydrate (3.58 mL, 115 mmol, 9 eq) was added. The mixture was stirred overnight at room temp.; completion was confirmed by UHPLC-MS (*gradient 1*). The solvent was evaporated and the residue was dissolved in H_2O (100 mL). The H_2O was evaporated and this step was repeated twice. Finally

the residue was re-dissolved in MeCN (100 mL), and filtered through a glass sinter, washing the solids with further MeCN. After evaporation of the solvent, tris(amino-octagol) nanostar **5** was obtained (10.8 g, 70%), and used without further purification

^{1}H NMR (400 MHz, $CDCl_3$) – Appendix 4a
δ=7.17 (s, 3H, Hub C**H**), 4.49 (s, 6H, Hub-C**H₂**O), 3.66-3.55 (m, 84H, PEG C**H₂**O), 3.46 (t, J=5.2 Hz, 6H, OC**H₂**CH_2NH_2), 2.81 (t, J=9.0 Hz, 6H, OCH_2C**H₂**NH_2), 1.89 (br.s, 6H, N**H₂**) ppm.

^{13}C NMR (101 MHz, $CDCl_3$) – Appendix 4b
δ=138.63 (3C, Hub **C**), 126.35 (3C, Hub **C**H), 73.36 (3C, O**C**$H_2CH_2NH_2$), 73.13 (3C, Hub-**C**H_2O), 70.65-70.58 (m, 36C, PEG **C**H_2O), 70.32 (3C, **C**$H_2OCH_2CH_2NH_2$), 69.56 (3C, Hub-CH_2O**C**H_2), 41.78 (3C, **C**H_2NH_2) ppm.

MS (ESI+): calcd for $C_{57}H_{111}N_3O_{24}$+3.H^+, [*M*+3.H]$^+$ = 408.3 , found *m/z* 408.4 (100%). [*M*+H]$^+$ = 1222.5 (1%). [*M*+2.H]$^+$ = 611.9 (25%).

1,3,5-Tris[(p-{(2,4-dimethoxyphenyl)aminomethyl}phenoxyacetamido)octa(ethyleneglycol)oxymethyl]benzene / [H-Rink-NH-Nanostar], 6. Nanostar **5** (4.05 g, 3.31 mmol) was dissolved in $CHCl_3$ (120 mL), to which solution were added Fmoc-Rink-CO_2H linker **S3** (7.14 g, 13.25 mmol, 4 eq), HOBt (2.24 g, 13.25 mmol, 4 eq) and DIC (2.07 mL, 13.25 mmol, 4 eq). After stirring at room temperature for 2 hours the reaction was complete; confirmed by UHPLC-MS (*gradient 1*). After evaporating the solvent, the residue was fractionated through a column of silanized silica, eluting with a gradient of MeCN-H_2O (20:90 to 90:10 v/v). Fractions 75:25 and 80:20 were combined and the MeCN evaporated at reduced pressure. An equal volume of saturated brine was added to the residual aqueous phase, which was extracted with $CHCl_3$. The extraction with $CHCl_3$ was repeated twice. The organic fraction was dried over Na_2SO_4 and evaporated to afford Fmoc-Rink-nanostar **S4** (8.08 g, 88%).

^{1}H NMR (400 MHz, $CDCl_3$) – Appendix 5a
δ=7.74 (d, J =7.8 Hz, 6H, Fmoc C**H**), 7.57 (d, J=7.5 Hz, 6H, Fmoc C**H**), 7.37 (dd, J =8.5, 6.4 Hz, 6H, Fmoc C**H**), 7.27 (dd, J=8.5, 6.4 Hz, 6H, Rink C**H**), 7.21 (s, 3H, Hub C**H**), 7.15-7.04 (m, 12H, Rink C**H** + Rink CON**H** + Fmoc C**H**), 6.81 (d, J=8.4 Hz, 6H, Rink C**H**), 6.47-6.43 (m, 6H, Rink C**H**), 6.02 (d, 3H, C**H**NH-Fmoc), 5.83 (d, J=8.7 Hz, 3H, CHN**H**-Fmoc) 4.51 (s, 6H, Hub-C**H₂**O), 4.44 (s, 6H, Rink OC**H₂**CONH), 4.43-4.39 (m, 6H, Fmoc OC**H₂**CH), 4.20 (t, J=6.9 Hz, 3H, Fmoc OCH_2C**H**), 3.79 (s, 9H, Rink OC**H₃**), 3.72 (s, 9H, Rink OC**H₃**), 3.64-3.50 (m, 96H, PEG C**H₂**O) ppm.

^{13}C NMR (101 MHz, $CDCl_3$) – Appendix 5b
δ=168.38 (3C, Rink NH**C**O), 160.61 (3C, Rink **C**OMe), 158.04 (3C, Rink **C**OMe), 156.25 (3C, Fmoc NH**C**O), 155.93 (3C, Rink **C**OMe), 143.97 (6C, Fmoc **C**), 141.39 (6C, Fmoc **C**), 138.63 (3C, Hub **C**), 135.78 (3C, Rink **C**), 129.58 (3C, Fmoc **C**H), 128.08 (6C, Rink **C**H), 127.74 (9C, Rink **C**H + Fmoc **C**H), 127.11 (6C, Rink **C**H + Fmoc **C**H), 126.42 (3C, Hub **C**H), 125.06 (6C, Fmoc **C**H), 120.02 (6C, Fmoc **C**H), 114.53 (6C, Rink **C**H), 104.23 (3C, Rink **C**), 99.43 (3C, Rink **C**H), 73.15 (3C, Hub-**C**H_2), 70.7-70.3 (m, 42C, PEG **C**H_2O), 69.83 (3C, O**C**H_2CH_2NH-Rink), 69.59 (3C, Rink O**C**H_2CONH), 67.51 (3C, Fmoc O**C**H_2CH), 55.60 (3C, Rink O**C**H_3), 55.50 (3C, Rink O**C**H_3), 54.75 (3C, Rink **C**HNH-Fmoc), 47.43 (3C, Fmoc OCH_2**C**H), 38.87 (3C, PEG OCH_2**C**H_2NH) ppm.

MS (ESI+): calcd for $C_{153}H_{192}N_6O_{42}$+3.NH_4^+, [*M*+3.NH_4]$^{3+}$ = 946.8; found m/z 946.8 (100%). [*M*+2.NH_4]$^{2+}$ = 1411.1 (30%). [*M*+4.NH_4]$^{4+}$ = 775.8 (1%).

The intermediate **S4** (8.08 g, 2.87 mmol) was re-dissolved in $CHCl_3$ (100 mL), to which DBU (11.16 mL, 74.63 mmol, 26 eq) and thiomalic acid (4.31 g, 28.70 mmol,10 eq) were added. After 1 hour the reaction was complete; confirmed by UHPLC-MS (*Gradient 1*). The reaction mixture was washed with potassium acetate buffer (pH 4.5), and the aqueous phase was back-extracted with $CHCl_3$. The combined organic phase was extracted with half-saturated potassium carbonate solution, and the aqueous phase was back-extracted with further $CHCl_3$. The organic phase was dried over Na_2SO_4 and evaporated to afford H-Rink-NH-nanostar **6** (5.8 g, 97%).

^{1}H NMR (400 MHz, $CDCl_3$) – Appendix 6a
δ=7.27 (d, J=8.6 Hz, 6H, Rink C**H**), 7.19 (s, 3H, Hub C**H**), 7.09-7.07 (m, 6H, Rink C**H** + CON**H**), 6.80 (d, J=8.0 Hz, 6H, Rink C**H**), 6.40-6.37 (m, 6H, Rink C**H**), 5.34 (s, 3H, Rink C**H**NH_2), 4.49 (s, 6H, Hub-C**H₂**O), 4.42 (s, 6H, Rink OC**H₂**CONH), 3.73 (s, 9H, Rink OC**H₃**), 3.72 (s, 9H, Rink OC**H₃**), 3.62-3.49 (m, 96H, PEG C**H₂**O), 2.13 (br.s, 6H, Rink N**H₂**) ppm.

^{13}C NMR (101 MHz, $CDCl_3$) – Appendix 6b
δ=168.48 (3C, Rink NH**C**O), 159.85 (3C, Rink **C**OMe), 157.70 (3C, Rink **C**OMe), 156.02 (3C, Rink **C**OMe), 138.93 (3C, Rink **C**), 138.62 (3C, Hub **C**), 128.33 (6C, Rink **C**H), 127.94 (3C, Rink **C**H), 126.82 (3C, Rink **C**H), 126.42 (3C, Hub **C**H), 114.46 (6C, Rink **C**H), 104.07 (3C, Rink **C**), 98.76 (3C, Rink **C**H), 73.14 (3C, Hub-**C**H_2), 70.70-70.30 (m, 42C, PEG **C**H_2O), 69.81 (3C, O**C**H_2CH_2NH), 69.58 (3C, Rink O**C**H_2CONH), 55.42 (6C, Rink O**C**H_3), 52.87 (3C, Rink **C**HNH_2), 38.83 (3C, PEG OCH_2**C**H_2NH) ppm.

MS (ESI+): calcd for $C_{108}H_{162}N_6O_{36}$+3.H^+, [*M*+3.H]$^{3+}$ = 707.7; found *m/z* 701.9 (100%). [*M*+2.H]$^{2+}$ = 1060.5 (4%). [*M*+4.H]$^{3+}$ = 522.5 (29%).

1,3,5-Tris[p-(3-O-{tert-butyl}threonyloxymethyl)phenoxy-octa(ethyleneglycol)oxymethyl]benzene / [H-Thr(tBu)-Wang-O-Nanostar], 8. Nanostar **4** (2.95 g, 1.92 mmol) was dissolved in DCM (30 mL), to which solution were added Fmoc-Thr(tBu)-OH (4.58

g, 11.52 mmol, 6 eq), pyridine (1.4 mL, 17.28 mmol, 9 eq), and finally DcbCl (1.65 mL, 11.52 mmol, 6eq). After stirring overnight at room temp. the reaction went to completion; confirmed by UHPLC-MS (*gradient 1*). After evaporating the solvent, the residue was fractionated through a column of silanized silica, eluting with a gradient of MeCN-H_2O (20:90 to 90:10 v/v). Fractions 80:20 and 85:15 were combined and the MeCN was evaporated ar reduced pressure. An equal volume of saturated brine was added to the residual aqueous phase, which was extracted with $CHCl_3$. The organic phase was dried over Na_2SO_4 and the solvent stripped off to afford crude Fmoc-Thr(tBu)-nanostar **7** (5.12 g, 95%).

Crude Fmoc-Thr(tBu)-nanostar **7** (2.43 g, 0.91 mmol) was re-dissolved in $CHCl_3$ (30 mL), to which DBU (3.52 mL, 23.56 mmol, 26 eq) and thiomalic acid (1.36g, 9.06 mmol, 10 eq) were added. The reaction went to completion within 1 hour, as confirmed by UHPLC-MS (*Gradient 1*). The reaction mixture was washed with potassium acetate buffer (pH4.5), and the aqueous phase back-extracted with $CHCl_3$. The organic phase was collected and extracted with half-saturated potassium carbonate solution, and the aqueous phase was back-extracted with $CHCl_3$. The organic phase was dried over Na_2SO_4 and the solvent stripped off to afford H-Thr(tBu)-O-Wang-nanostar **8** (1.91 g, 100%).

^{1}H NMR (400 MHz, $CDCl_3$) – Appendix 7a
δ=7.25 (d, J=8.7 Hz, 6H, Wang C**H**), 7.19 (s, 3H, Hub C**H**), 6.86 (d, J=8.7 Hz, 6H, Wang C**H**), 5.09 (d, J=11.9 Hz, 3H, Wang-C**H₂**O), 4.94 (d, J=11.9 Hz, 3H, Wang-C$\mathbf{H_2}$O), 4.51 (s, 6H, Hub-C$\mathbf{H_2}$O), 4.09 (dd, J=5.7, 4.0 Hz, 6H, CH_2C$\mathbf{H_2}$O-Wang), 3.96 (qd, J=6.2, 3.3 Hz, 3H, Thr C**H**O-tBu), 3.82 (dd, J=5.6, 4.1 Hz, 6H, C$\mathbf{H_2}$$CH_2$O-Wang), 3.70-3.57 (m, 87H, PEG C$\mathbf{H_2}$O + Thr C**H**NH_2), 3.24 (b.d, J=3.3 Hz, 6H, N$\mathbf{H_2}$), 1.18 (d, J=6.3 Hz, 9H, Thr C$\mathbf{H_3}$), 1.07 (s, 27H, tBu C$\mathbf{H_3}$) ppm.

^{13}C NMR (101 MHz, $CDCl_3$) – Appendix 7b
δ=175.08 (3C, **C**OO), 159.05 (3C, Wang CH_2O**C**), 138.67 (3C, Hub **C**), 130.76 (6C, Wang **C**H), 128.25 (3C, Wang **C**CH_2O), 126.41 (3C, Hub **C**H), 114.47 (6C, Wang **C**H), 73.72 (3C, **C**HO-tBu), 73.18 (3C, Hub-**C**H_2), 70.79 [3C, tBu O**C**$(CH_3)_3$], 70.78-70.30 (m, 42C, **C**H_2O), 69.74 (3C, O**C**H_2CH_2O-Ar), 69.56 (3C, **C**H_2O-Wang), 66.68 (3C, Wang-**C**H_2-O-Thr), 60.73 (3C, **C**HNH_2), 28.57 (9C, tBu **C**H_3), 20.90 (3C, Thr **C**H_3) ppm.

MS (ESI+): calcd for $C_{103}H_{173}N_3O_{35}$+3.H^+, $[M+3.H]^{3+}$ = 672.1; found *m/z* 672.6 (100%). $[M+H]^+$ = 2014.8 (0.27%). $[M+2.H]^{2+}$ = 1008.4 (25%).

4. Variation of reaction kinetics with solvent

The solvent must simultaneously facilitate fast reactions and solubilize all reagents, including the peptide-nanostar. Solvent polarity impacts both reaction kinetics and solubility, and is therefore a critical parameter. We screened candidate solvents reported to be compatible with peptide synthesis, covering a wide range of polarity: 2-Methyltetrahydrofuran (MeTHF), THF, N-methyl-2-pyrrolidone (NMP) and dimethyl sulfoxide (DMSO) exhibit low, medium, high, and very high polarity, respectively, based on the Reichardt scale (E_T^N). Considering that a single solvent may not optimally fulfil all the requirements, binary mixtures of THF-NMP in different proportions (80:20, 50:50 and 35:65) were also evaluated to cover the region of medium to high polarity, estimating the polarity by linear interpolation from the solvent ratio. It was intended that each component would compensate for the limitations of the other.

Table S1: Polarities of candidate solvents.

Candidate Solvents	Polarity ($E^T{}_N$)
Me-THF	0.187
THF	0.207
THF-NMP 80:20	0.237
THF-NMP 50:50	0.281
THF-NMP 35:65	0.303
NMP	0.355
DMSO	0.444

A dipeptide model system was studied to determine the effects of different solvents: a selection of Fmoc-AA-OH [AA = Val, Leu, Glu(trt), Asp(OtBu), Trp(Boc), Arg (Pbf), Ala] were coupled to H-Phe-OMe; the crude products of coupling were then subjected to Fmoc removal. Fmoc-Val-OH (339.4 g mol^{-1}), Fmoc-Leu-OH (353.4 g mol^{-1}) and Fmoc-Ala-OH (311.3 g mol^{-1}) are non-polar amino acids with aliphatic side-chains and have relatively small molecular weights. Fmoc-Trp(Boc)-OH (526.6 gmol^{-1}) is also non-polar but with an aromatic side-chain. Fmoc-Asp(OtBu) (411.5 g mol^{-1}), Fmoc-Arg(Pbf)-OH (648.8 g mol^{-1}), and Fmoc-Gln(Trt)-OH (610.7 g mol^{-1}) are polar with functionalized side-chains. Fmoc-Arg(Pbf)-OH (648.8 g mol^{-1}) is the largest protected AA, whereas Fmoc-Ala-OH (311.3 g mol^{-1}) is the smallest. Reactions were carried out on Radleys Carousel Reaction Station^{TM} with 12 reaction tubes of 20mL volume each. During each round, 2 to 4 reactions were carried out simultaneously with stirring and heating. A temperature probe was used to maintain the reaction station at 30 °C.

Determination of the coupling kinetics. To each reaction tube was added Fmoc-amino acid (0.422 mmol), followed by HOBt (135 mg, 0.422 mmol), and the mixture was dissolved in candidate solvent (5 mL), stirring at 30°C. Upon full dissolution, DIC (65.9 μL, 0.422 mmol) was added and stirring continued. After 10 minutes, H-Phe-OMe (272 mg, 1.268 mmol) dissolved in candidate solvent (5 mL) was added to each reaction tube. Samples of reaction solution (10 μL) were withdrawn at 0.5, 5, 10, 20, 30, 60 and 120 minutes and immediately diluted 100-fold into MeCN (1 mL) to quench the reaction. The sample solutions were analyzed by UHPLC-MS (*Gradient 2*), dentifying each peak by MS. The UV peak areas of amino acids and dipeptide product in the chromatogram were integrated and used for calculating the rate constant. The above procedure was repeated with all candidate solvents, one by one.

The coupling reaction obeys second-order kinetics. [1] The rate constant, $k_{coupling}$ (L.mol^{-1}.min^{-1}), was determined by fitting the experimental data to the second-order rate Eqn. S1, with amino acid concentration, [AA] (mol.L^{-1}) and time (t, min). Excel Solver was used to minimize the sum of the square of the error to determine the best fit.

$$Equation\ S1:\ \frac{1}{[AA]} = \frac{1}{[AA_0]} + k_{coupling}t$$

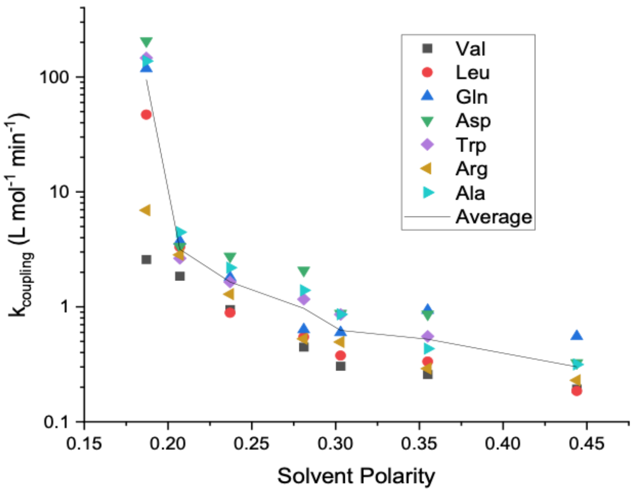

Figure S2: The rate constant, $k_{coupling}$ (L.mol^{-1}.min^{-1}) for the formation of Fmoc-dipeptide (Fmoc-AA-Phe-OMe) tested with 7 types of amino acids in various solvent candidates.

Determination of the kinetics for Fmoc removal kinetics . Upon the completion of coupling reactions, confirmed by the UHPLC-MS where peak areas stopped changing, piperidine (1.1 mL, final 10% v/v) was added to each reaction tube to initiate the Fmoc removal. Reactions were stirred and maintained at 30°C. Samples of reaction solution (10 μL) were withdrawn at 0.5, 5, 10, 20, 30, 60 and 120 minutes after adding piperidine and immediately diluted 100-fold into MeCN (1 mL) to quench the reaction. The samples were analyzed by UHPLC-MS (*Gradient 2*), identifying each peak by MS. The UV peak areas of dipeptide product were integrated and used for calculating the rate constant.

To determine the rate constant of Fmoc removal, first-order reaction kinetics were assumed Eqn. S2, using Excel Solver to minimize the sum of the square of the errors to determine the best fit to experiment by varying the $k_{deprotection}$ (min^{-1}).

$$Equation\ S2:\ [dipeptide] = [dipeptide]_0 e^{-k_{deprotection} t}$$

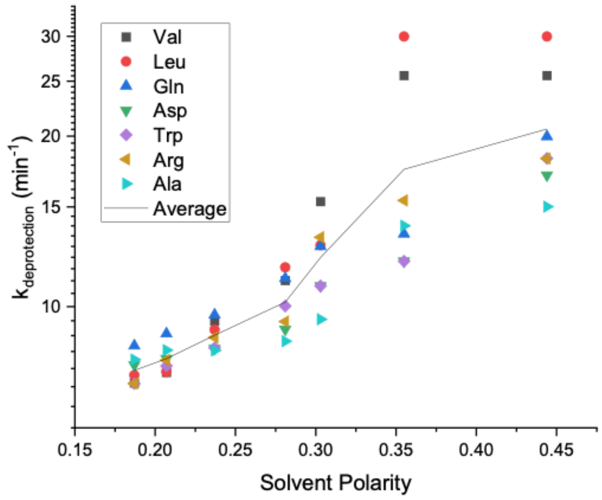

Figure S3: The $k_{deprotection}$ (min^{-1}) for the formation of H-dipeptide-OMe (Fmoc removal of Fmoc-AA-Phe-OMe) tested with 7 types of amino acid in various candidate solvents.

From these data it could be concluded that the primary rate determining factor for both coupling and Fmoc removal reactions is solvent polarity.

For NMP-THF mixtures there was a strong correlation between reaction rates and polarity (exponential fit; R2 coupling > 0.95, R2 deprotection > 0.98, Figure S2), allowing tuning of the solvent mixture.

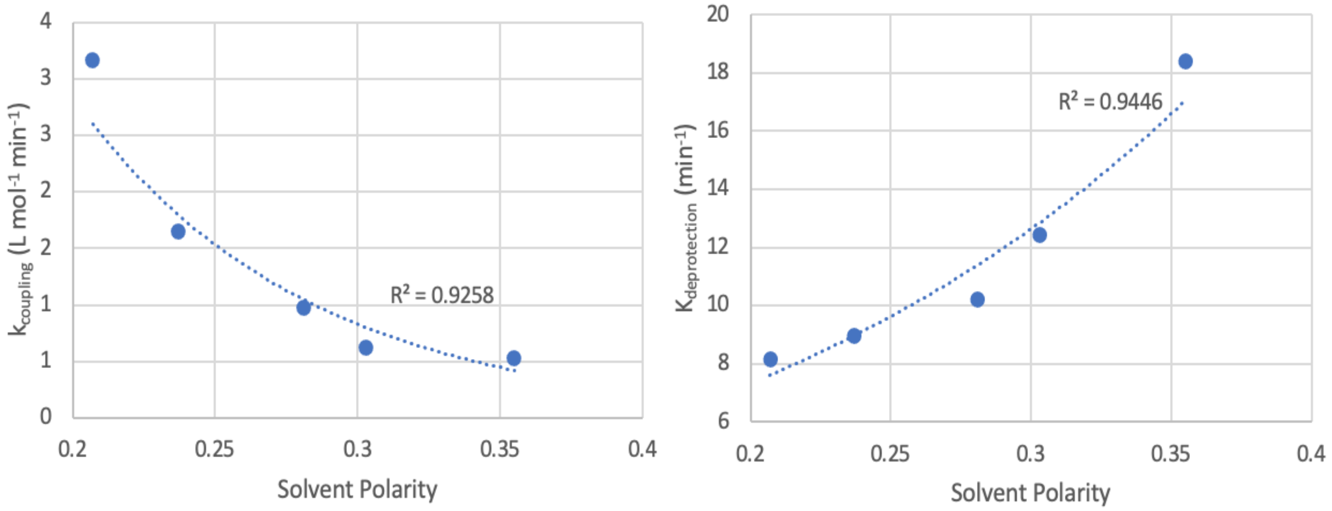

Figure S4: The exponential correlation between $k_{coupling}$ (L.mol^{-1}.min-^{1}) (Left) and polarity of THF-NMP solvent mixture. $k_{deprotection}$ (Right) .

Solubility. The solubilities of amino acids and their corresponding dipeptides formed in candidate solvents were determined qualitatively via visual observations during coupling and Fmoc removal reactions. The solubilizing powers of solvents during coupling and Fmoc removal were reported as: (a) soluble, (b) partially soluble and (c) insoluble, as shown in Figure S5. The results are summarized in Table S2 with green, yellow and red colors denoting soluble, partially soluble and insoluble, respectively. During coupling, an opalescence white color appeared in Me-THF after 10 mins. Although the urea by-product appeared to have good solubility in THF at around 1.0 M (0.42 mmol in 5 mL during coupling), we experienced the same solubility issue at concentration higher than 1.0 M. High polarity NMP and DMSO performed better. Solvent mixtures tend to exhibit solubilizing properties close to the predominant component. Compositions with higher than 50% NMP performed better in solubilizing dipeptides after Fmoc removal.

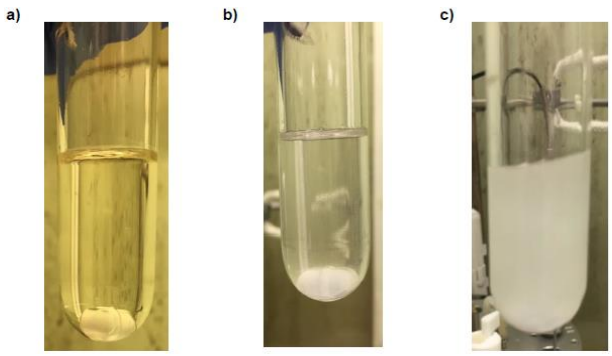

Figure S5: Qualitative solubility test via visual observation: (a) soluble, (b) partially soluble, and (c) insoluble.

Table S2: Solubility test results for corresponding di-peptides during coupling and Fmoc removal with green, yellow and red colors representing soluble, partially soluble, and insoluble, respectively.

Amino Acids / Solvents	Coupling solubilities				Fmoc removal solubilities			
	MeTHF	THF	NMP	DMSO	MeTHF	THF	NMP	DMSO
Val								
Leu								
Gln								
Asp								
Trp								
Arg								
Ala								

5. Nanostar coupling and Fmoc removal kinetics study.

Reactions were carried out in a Radleys Carousel Reaction Station™ with 12 reaction tubes of 20 mL volume each. During each round, 1 to 2 reactions were carried out simultaneously with stirring and heating. A temperature probe was used to maintain the reaction station at 30 °C. [H-Thr(tBu)-O-Wang]$_3$-nanostar **8** (60 mg, 0.03 mmol) was added to a reaction tube containing a solvent mixture of THF-NMP 35:65 (3 mL). In a separate vial, Fmoc-Asn(Trt)-OH (89 mg, 0.15 mmol) and HOBt (25 mg, 0.19 mmol) were dissolved in THF-NMP 35:65 (3 mL). DIC (23 μL , 0.15 mmol) was then added to activate the Asn. After 10 mins, the solvent mixture containing activated Asn was transferred to the reaction tube containing the nanostar, making a total of 6 mL reaction mixture at 1 wt% [H-Thr(tBu)-O-Wang]$_3$-nanostar **8**. Samples of reaction solution (10 μL) were withdrawn at 0.5, 5, 10, 20, 30, 60 and 120 minutes after adding activated Asn and immediately diluted 100-fold into MeCN (1 mL) to quench the reaction. The samples were analyzed by UHPLC-MS (*Gradient 1*), and each peak was identified by MS. The UV peak areas corresponding to [Fmoc-Asn(Trt)Thr-O-Wang]-(H-Thr-O-Wang)$_2$-nanostar (1-arm, **13**), [Fmoc-Asn(Trt)Thr-O-Wang]$_2$-(H-Thr-O-Wang)-nanostar (2-arm, **14**) and Fmoc-dipeptide-nanostar **9** over reaction time were integrated (Fig. S6a). After reaction completion, when all peptide-nanostar turned into Fmoc-dipeptide-nanostar **9**, piperidine (660 μL, 10 v/v%) was added to initiate the Fmoc removal reaction. Again, samples (10 μL) were withdrawn at 0.5, 5, 10, 20, and 30 minutes after adding piperidine, and immediately diluted by 100-fold into MeCN (1 mL) to quench the reaction. The samples were analyzed by UHPLC-MS (*Gradient 1*) and peaks identities confirmed by MS and the UV peak areas over reaction time were integrated (Fig. S6b). The above procedure was repeated with the following conditions: 2 wt% [H-Thr(tBu)-O-Wang]$_3$-nanostar **8** & 10 v/v% piperidine; and 5 wt% [H-Thr(tBu)-O-Wang]$_3$-nanostar **8** & 10 v/v% piperidine with the same molar equivalent of |Fmoc-Asn(Trt)-OH, DIC and HOBt (1.7 eq per arm).

Table S3: Time-course of coupling (to form Fmoc-dipeptide-nanostar **9**) and Fmoc removal (to form H-dipeptide-nanostar **10**) reactions at 1, 2 and 5 wt% of [H-Thr(tBu)-O-Wang]$_3$-nanostar **8**.

[H-Thr(tBu)-O-Wang]$_3$-nanostar 8	1 wt%	2 wt%	5 wt%
**Coupling Completion (%)**			
50 %	25.0-27.0 min	8.0-9.0 min	0.1-0.2 min
90 %	105.0-107.0 min	18.0-19.0 min	1.1-1.2 min
> 99.8 %	> 240 min	< 60 min	< 2 min
**Fmoc removal Completion (%)**			
50 %		0.3-0.5 min	
90 %		1.7-1.9 min	
> 99.8 %		< 2 min	

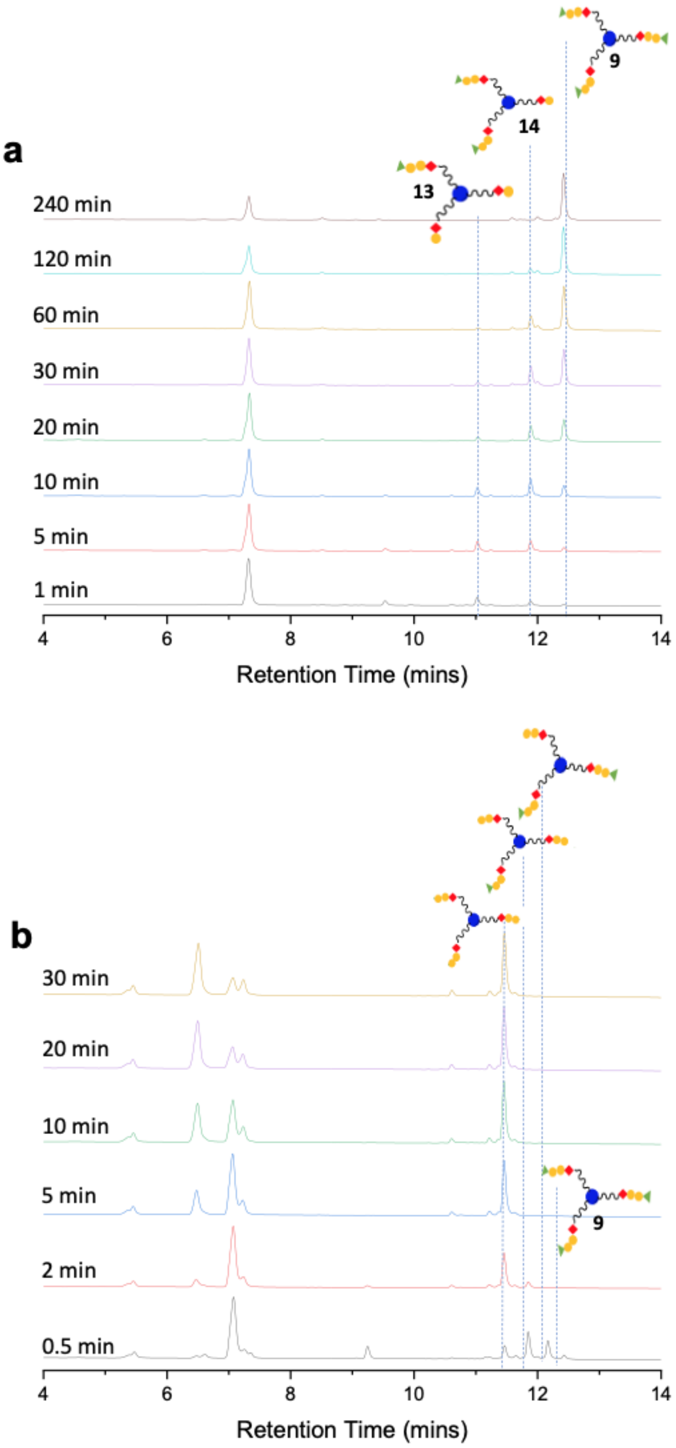

Figure S6: UHPLC-MS results obtained from 1wt% run as example:a) UV peak areas corresponding to [Fmoc-Asn(Trt)Thr-O-Wang]-(H-Thr-O-Wang)$_2$-nanostar (1-arm, **13**), [Fmoc-Asn(Trt)Thr-O-Wang]$_2$-(H-Thr-O-Wang)-nanostar (2-arm, **14**) and Fmoc-dipeptide-nanostar **9**. b) The transition of peptide-nanostar during Fmoc removal to form H-dipeptide-nanostar **10.**

6. Membrane fabrication and performance

The membrane must satisfy two critical criteria: First and foremost, the membrane must withstand aggressive organic solvents, highly reactive intermediates and strong bases for long durations. Second, the membrane needs to separate peptide-nanostar from reaction by-products efficiently. PBI_2005(1), PBI_2005(2), and PEI_2005 were screened for this purpose.

PBI membrane fabrication. Polybenzyimidazole (PBI) membrane modified with polymer brushes was first prepared by fabricating PBI flat sheet membrane via phase inversion. Celazole® PBI S26 solution, contains 26 wt% PBI solids in conjunction with 1.5 wt% LiCl salt stabilizer in DMAc, was diluted to 18 wt% dope solution with dimethyacetamide (DMAc). The dope solution was mixed on a roller overnight to achieve homogeneity, then filtered using a stainless sieve with a pore size of 11 μm to remove particles. It was then left standing still for several hours to remove gas bubbles before casting.

Membrane PBI_2005(1): The dope solution was cast on polypropylene nonwoven support (Novatek 2471) using a continuous casting machine (SepraTek, Korea) and immediately immersed in deionized water bath (21 °C) to induce phase inversion, forming flat sheet membrane (length x width = 1.2 m x 0.3 m). The casting knife was set at 200 μm and the nonwoven support was dragged across the casting knife at a steady speed of 3 cm.s^{-1}. After removing the membrane from the water bath, it was rolled up together with an equal length of spacer to allow solvent ingress to the roll, and then soaked in IPA for one day, before storing in MeCN for 2 weeks. After this, the PBI membrane roll was washed with dry MeCN and then cross-linked using α,α′-dibromo-p-xylene (DBX, 2.5 wt%) in dry MeCN at 70 °C overnight. After cross-linking, the membrane was washed with dry MeCN and then modified with Jeffamine® M-2005 (33% v/v) at 70 °C for 24 h. The membrane was rinsed with MeCN and left overnight in PEG solution (PEG 400-IPA 1:1). Finally, the membrane roll was taken out of the PEG solution and air-dried before storing.

Membrane PBI_2005(2): PBI_2005(2) was prepared similarly to PBI_2005(1), but was stored for 4 weeks in MeCN before crosslinking.

PEI membrane fabrication. Polyetherimide (PEI) ULTEM^{TM} 1000 powder was evacuated at 100 °C for 12 h before use. The PEI powder was dissolved in a mixture of GBL-NMP (70:30 wt/wt) by stirring at 70 °C for one day to form a dope solution of 23 wt%. The solution was filtered using nylon net filter (11 μm pore size, Millipore) and stored for a day at room temp. to remove gas bubbles. The dope was the cast on polypropylene nonwoven support (Novatex 2471) using a continuous casting machine (*SepraTek*, Korea) with the casting knife set at 150 μm, and casting speed of 5 rpm. The freshly cast film was plunged into a water bath set at 19 °C to induce phase inversion. The PEI membrane roll was cross-linked in 5 wt% of diaminopropane solution in MeOH for 1 day at 20 °C. A cross-linked PEI membrane sheet (approx. A5 size) was placed in a tube (200 mL), rinsed with dry MeCN and stored under Ar atmosphere. A solution of terephthaloyl chloride (TPC, 100 mM) and Hunig's base (200 mM) in dry MeCN (150 mL) was prepared in another tube under Ar atmosphere. The mixed solution was poured into the tube containing PEI membrane sheet. After 3 h, the liquid was decantated from the membrane, and the membrane was rinsed with dry MeCN. Next, MeCN (83 mL), Jeffamine® (50 mL) and Hunig's base (17 mL) were added into the membrane tube and stirred for 12 h. The liquid was then decantated, and the membrane rinsed with MeOH. The membrane was dried and stored at room temperature without further treatment.

Membrane Screening (Performance Test). Since OSN is a size-selective separation, the most difficult solutes to separate from the product peptide-nanostar will be the highest MW by-products. Therefore it was anticipated that DBF-Pip **12** (MW = 263 Da), and quenched building block, H-AA-Pip, of which the large H-Asn(Trt)-Pip **11** (MW = 449 Da) would be a difficult species to permeate while selectively retaining the desired peptide-nanostars. This is particularly acute at the beginning of a peptide synthesis run (i=1 and 2) when the growing peptide-nanostars have their lowest MW. Therefore a screening solution containing H-Thr(tBu)-O-Wang-nanostar **8** (MW = 2013 Da), H-Asn(Trt)-Thr(tBu)-O-Wang-nanostar **10** (MW = 3082 Da), H-Asn(Trt)-Pip 11 and DBF-Pip **12** (1 g.L^{-1} each, see Fig. 2 and Scheme 1) in THF was prepared.

Membrane screening was carried out in the rig described in Figure S7. PBI_2005(1) flat sheet was cut into 14 cm^2 disks and housed in membrane cells. Screening solution was prepared by dissolving H-Thr(tBu)-O-Wang-nanostar **8** (MW = 2013 Da), H-Asn(Trt)-Thr(tBu)-O-Wang-nanostar **10** (MW = 3082 Da), H-Asn(Trt)-Pip **11** and DBF-Pip **12** (1 g.L^{-1} each, see Fig. 2) in THF. An HPLC pump (reciprocating pump) was used to pump the screening solution from the feed tank to the cells at a rate of 75 mL.min^{-1}, while a circulation pump was used to provide crossflow at 90 L.h^{-1} at the retentate side. The pressure of the system was regulated by a pressure relief valve that returns retentate back to the feed tank. The permeate from the membrane cells was returned to the feed tank to form a closed circulation system. During the screening process, the system was pressurized to 20 bar and maintained undisturbed for at least an hour to allow it to reach a steady state. After that samples were taken from each cell's permeate line and from the feed tank. Another sampling was done after 5 hours. At the end of the screening, the screening solution was recovered via the recovery wash line, evaporated and reused in the next screening. PBI_2005(2) and PEI_2005 were tested in the same manner.

The performance of a membrane is defined by two main parameters: solute rejection and solvent permeance. The rejection of a given solute, i.e. one of the four molecules in the screening solution, was determined from its relative concentrations in the permeate and retentate (sample from feed tank) sides, according to Eqn. S3. The difference in rejection between two molecules is related to how efficiently a membrane can separates them, according to the separation factor defined by Eqn. S4. The higher the separation factor, the more efficient the membrane is at separating the product from reagents and reaction by-products. The speed of solvent flow through a membrane is also a major factor in membrane selection, because high permeance ensures rapid diafiltration..

Permeance, Eqn. S5, is measured in $L.m^{-2}h^{-1}bar^{-1}$, where A is membrane area and TMP is transmembrane pressure. PEI_2005 showed the highest permeance in THF (12.4 $L.m^{-2}h^{-1}bar^{-1}$), compared to both PBI_2005(1) (7.2 $L.m^{-2}h^{-1}bar^{-1}$) and PBI_ 2005(2) (4.0 $L.m^{-2}h^{-1}bar^{-1}$). Even so, PBI_2005(1) was selected for further study because of its balance between a good separation factor and higher permeance. Membrane characteristics are summarized in Table S4.

$$Equation\ S3:\ Rej\ (\%) = 1 - \frac{Permeate\ Concentration}{Retentate\ Concentrate}$$

$$Equation\ S4:\ Separation\ factor\ (\beta_{Product/Byproduct}) = \frac{1 - Rej(Byproduct)}{1 - Rej(Product)}$$

$$Equation\ S5: Permeance\ (Lm^{-2}h^{-1}bar^{-1}) = \mathrm{Flux}(\mathrm{L\ h^{-1}}) * \frac{1}{A\ (m^2) * TMP\ (bar)}$$

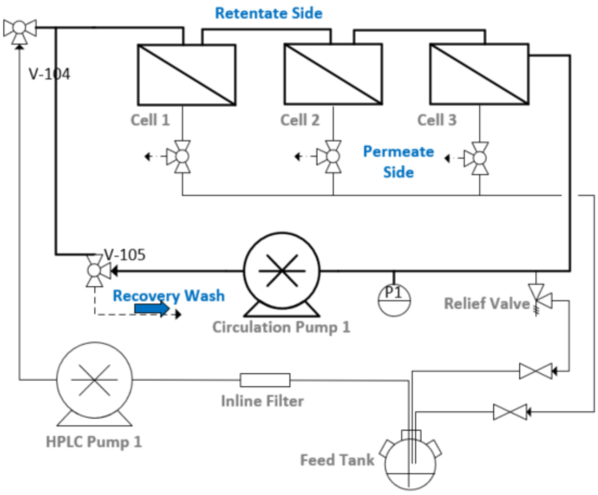

Figure S7: Membrane screening rig.

Table S4. Membrane performances

	**PBI_J2005(1)**	**PBI_J2005(2)**	**PEI_2005**
**Rejection (%)**			
H-Asn(Trt)-Thr(tBu)-O-Wang-nanostar, **10**	93.3±0.5	89.4±1.0	84.0±1.4
H-Thr(tBu)-O-Wang-nanostar, **8**	89.8±1.8	91.0±1.4	77.5±0.7
H-Asn(Trt)-Pip, **11**	37.8±3.9	53.0±2.8	27.3±2.2
DBF-Pip, **12**	16.5±1.3	29.1±2.7	11.1±1.2
**Selectivity ($\beta_{10/11}$)**	9.2	4.4	4.7
**Selectivity ($\beta_{8/11}$)**	6.1	5.2	3.2
**Permeance ($L.m^{-2}\ h^{-1}\ bar^{-1}$)**	7.2±0.6	4.0±0.5	12.4±0.6

7. Synthesizer operation

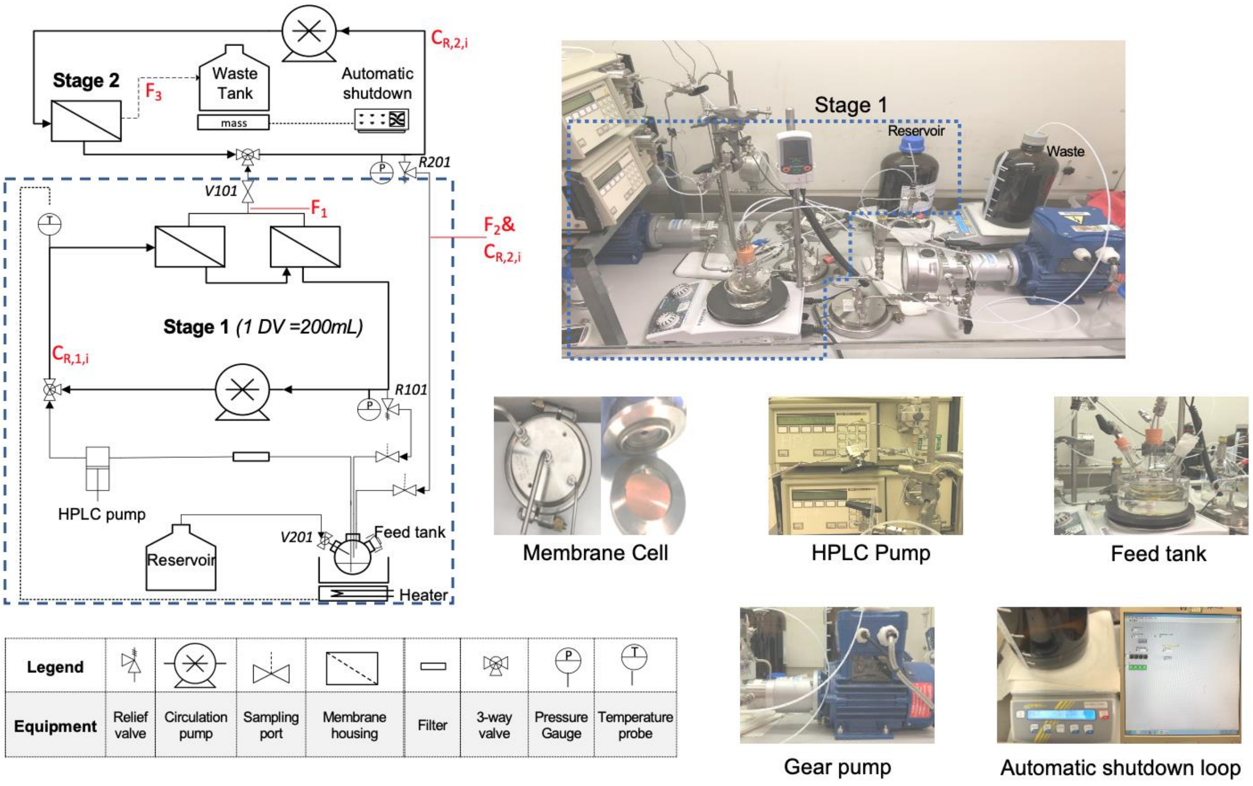

Figure S8: The layout of a synthesizer with two stages (left), and photographs of the synthesizer and equipment (right).

The protocols for running the equipment are more complex and all the interior of the combined reactor-separator unit, including membranes and compressible pressure seals, must withstand not just aggressive solvents, but also chemically highly reactive species.

Synthesizer operating protocol. During reactions, the HPLC pump and stage 1 circulation pump were maintained at 75 mL.min^{-1} and 90 L.h^{-1} respectively, with the pressure relief valve fully open to ensure complete mixing. The valve to stage 2 (V101) was closed, and the recycling line from stage 2 relief valve (R201) to stage 1 disengaged. The heating was maintained at 35°C in stage 1 via a feedback control.

After Fmoc removal was complete, V101 was opened and the recycling line was connected to the feed tank. Stage 2 gear pump was turned on at 90 L.h^{-1}, while the pumps in stage 1 maintained the same flow. Reservoir inlet valve (V201) was opened to enable the addition of fresh solvent (THF-NMP, 35:65) from the solvent reservoir into the feed tank during diafiltration; while permeate line from stage 2 was routed to the waste tank. The heating was turned off because turbulent flow in stage 1 loop maintained the temperature around 30°C. Next, the mass balance was reset to 0 and the National Instrument (NI) system automatic shutdown setpoint adjusted to 2000 g (d = 0.98 g.mL^{-1}). Subsequently, stages 1 and 2 were pressurized to set points gradually by closing the pressure relief valves to restrict the retentate flows back to the feed tank. It has been shown that a 50% recycle ratio (RR), i.e. returning from stage 2 half the volume that permeated from stage 1, maximizes the yield and purity [2]. The RR is mainly passively controlled by having twice as many membrane cells in stage 1 as stage 2, and setting equal trans-membrane pressure drops in both stages (i.e. twice the flux through stage 1 as stage 2), but can be modulated using the stage 2 pressure relief valve. To achieve RR = 50%, stage 1 was pressurized to 40 bar and stage 2 at 20 bar to give trans-membrane pressures of 20 bars in both stages. During diafiltration, a low pseudo-steady-state concentration of dipeptide-nanostar **10** built up in stage 2, which was largely returned to stage 1 via the stage 2 pressure relief valve. 10 *diavolumes* (DV, 1 DV = 200 mL) were passed through which took about 15 h (in THF-NMP 35:65). The power sources of all pumps were connected to the National Instrument (NI) system, which switched them off when the mass balance reading reached 2000 g. This shutdown stopped diafiltration, and both stages slowly depressurized to 0 bar. After the shutdown, pressure relief valves (R101, R201) were opened, V101 was closed, and the stage 2 recycling loop (R201) was disengaged. This disconnected stage 2 from the reactor part of the synthesizer before starting the reactions. While the reactions were proceeding in stage 1, stage 2 was cleaned with fresh solvent, and then remained idle until the next diafiltration.

To synthesize a full-length peptide, this cycle was repeated until the peptide-nanostar reached target length. The product was then recovered from stage 1 by washing with THF-NMP 35:65. To monitor the diafiltration process, stages 1and 2 were sampled from the sampling ports using syringes, while permeate from stage 2 was collected. The samples were diluted with MeCN in 10:90 ratio before analyzing by UHPLC-MS (*Gradient 2*) and GC.

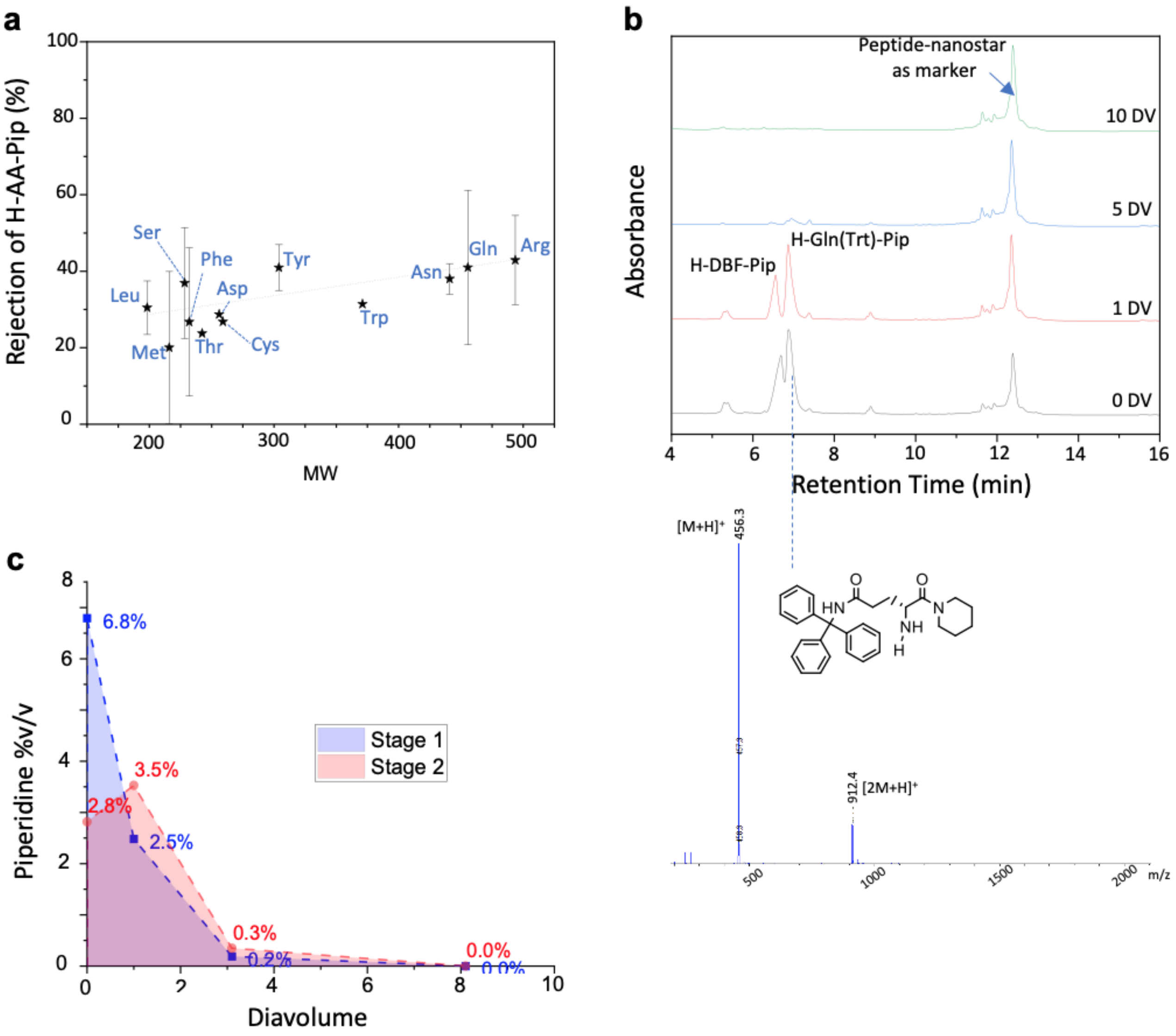

Figure S9: a) The observed rejections of different H-AA-Pip by membrane PBI18_2005(1) during diafiltration. The observed rejection of H-AA-Pip was determined to be in the range of ~20% - 40%, which can be removed efficiently with 10 DV. b) Removal of H-AA-Pip during diafiltration with H-Gln(Trt)-Pip as an example, the large H-Gln(Trt)-Pip (MW=455.6) was completely removed after passing through 10 DV, while the peptide-nanostar marker retained in stage 1. c) The reduction of piperidine v/v% in stages 1 and 2 over the diafiltration.

H-AA-Pip and piperidine removals. Apart from H-Asn-Pip, same synthesizer protocol was later extended to other amino acids to permeate H-AA-Pip covering a wide range of molecular weight. All H-AA-Pip tested could be removed after passing through 10 DV of THF-NMP 35:65. To monitor the diafiltration process, stages 1and 2 were sampled from the sampling ports using syringes, while permeate from stage 2 was collected. The samples were diluted with MeCN in 10:90 ratio before analyzing by UHPLC-MS (*Gradient 2*) and GC (see characterizations and measurements). The observed rejection of H-AA-Pip calculated with Eqn. S3 (see section 6). The removal of piperidine from the synthesizer during defiltration was monitored with GC. A calibration curve for piperidine was constructed by running 0.01%, 0.10%, 1.00%, 5.00% and 10.00% piperdine in THF-NMP 35:65. Piperidine concentrations relative to the THF:NMP 35:65 in the synthesizer after diafiltering for certain diavolumes were estimated based on the calibration curve.

8. Modelling and simulation (single-stage vs two-stage)

A model was built in MATLAB software to simulate the diafiltration process of single-stage and two-stage diafiltration systems. Both systems are illustrated in Figure S8 (left); the single-stage diafiltration system is the region within the blue box. To model the separation of peptide-nanostar from byproducts, two mass balance models were built to simulate their mass flows in single-stage and two-stage closed systems. The model reported by J.F. Kim et al. was used as a reference [2]. The following assumptions were made to simplify the models: the system volume remains constant throughout the diafiltration process; the system is in perfect homogenous condition; and the membrane discs of PBI_2005(1) installed in the system have uniform performances. The system volume was kept constant, i.e. constant volume diafiltration (CVD), by the passive addition of fresh solvent from the reservoir, controlled by the slight vacuum built up in the feed tank as solvent drains from the system into the permeate. Circulation pumps were installed at each stage to maintain high cross-flow rates in the systems, providing turbulence and mixing. Thus, the effect of concentration polarization is omitted here. Loaded PBI_2005(1) cells were screened before commencing diafiltration to ensure no defective membranes were placed in the synthesizer. The mass balance for the single-stage closed system was modelled with differential Eqn. S6 to describe the rate of change of concentration in stage 1:

$$Equation\ S6:\ V_{stage1}\frac{dC_{R,1,i}}{dt} = -F_1\, C_{R,1,i}\,(1 - Rej_i)$$

$$Equation\ S7: F_1 = P\ A\ \Delta P$$

where $V_{stage\ 1}$ (200 mL) is stage 1 volume, $C_{R,1,i}$ is the retentate concentration of solute i, t is the diafiltration time (h), F_1 is the permeate flow rate ($L.h^{-1}$), and Rej_i is the apparent rejection of solute i. Three solutes of interest are H-dipeptide-nanostar **10**, H-Asn(Trt)-Pip **11**, and DBF-Pip **12** with experimentally measured rejections of 93.3%, 37.8% and 16.5%, respectively, obtained from membrane screening results. F_1 is calculated from Eqn. S7, where P is the membrane permeance ($L.m^{-2}.h^{-1}.bar^{-1}$), A is the membrane area (m^2), and ΔP is transmembrane pressure (bar). The membrane permeance measured in THF (7.2 $L.m^{-2}.h^{-1}.bar^{-1}$). Three differential equations, one for each species, were solved simultaneously with MATLAB's in-built 'ode23' function from t = 0 to 10 hours, in steps of 0.05 hour. The input parameters for 'ode23' were the solutes' initial molar concentrations. At t=0, the molar concentrations of H-dipeptide-nanostar **10** was 7.25 $x10^{-3}$ M (1 wt%), with the other two 5 times higher than that value. The membrane area of a membrane housing was measured to be $5.4x10^{-3}$ m^2 with two membrane cells installed in stage 1. Stage 1 was running at 20 bar permeating to the atmosphere. After solving the differential equations numerically over a period of 10 h, the molar concentrations of all species of interest at every 0.05 hour were obtained. The molar purities and yields over the 10 hours diafiltration time, also evaluated every 0.05 hour, were calculated with equations S8 and S9, respectively:

$$Equation\ S8: Molar\ purity = \frac{\text{Peptide nanostar}}{\text{Peptide nanostar} + \text{AA\_pip} + \text{Fmoc\_Pip}}$$

$$Equation\ S9: Molar\ yield = \frac{\text{Peptide nanostar final}}{\text{Peptide nanostar initial}}$$

A similar approach was taken for the two-stage diafiltration system, with a modified ordinary differential question to include the recycle flow from stage 2 with flow rate F_2,

$$Equation\ S10: V_{stage1}\frac{\mathrm{dC_{R,1,i}}}{\mathrm{dt}} = -\mathrm{F_1}\ C_{R,1,i}(1 - Rej_i) + F_2\ C_{R,2,i}$$

$$Equation\ S11:\ V_{stage2}\frac{\mathrm{dC_{R,2,i}}}{\mathrm{dt}} = \mathrm{F_1}\ C_{R,1,i}(1 - Rej_i) - F_2\ C_{R,2,i} - F_3\ C_{R,2,i}(1 - Rej_i)$$

where $V_{stage\ 2}$ (100 mL) is the volume of the second stage, $C_{R,2,i}$ is the retentate concentration of solute i in stage 2, F_3 is the permeate flow rate from stage 2 ($L\ h^{-1}$). F_3 was calculated using the same equation as F_1 but with only one membrane housing in stage 2 and 20 bar transmembrane pressure. Stage 1 is maintained at 40 bar with effective ΔP transmembrane pressure of 20 bar (P_{stage1} – P_{stage2}), and 2 membrane housings. F_2 is determined by the difference F_1-F_3. For a two-stage diafiltration system, the recycle ratio (RR) was calculated from Eqn. S12 to estimate the flow ratio being recycled from stage 2 back to stage 1. Since the transmembrane pressure and membrane permeability of both stages are kept similar, F_3 is half the value of F_1.

$$Equation\ S12:\ Recycling\ ratio\ (RR) = 1 - F_3/F_1$$

9. Enkephalin-like model peptide synthesis

Compound **6** (3.1 g, 1.46 mmol, ~1.7 wt%) was dissolved in THF-NMP 35:65 then added gradually to the synthesizer via the feed tank. The HPLC and gear pumps were turned on to provide adequate mixing and dissolution in stage 1**.** In a separate vial, Fmoc-Leu-OH (2.58 g, 7.29 mmol) and HOBt (1.23 g, 9.11 mmol) were dissolved in the minimum amount of solvent. Once fully dissolved, DIC (0.12 mL, 7.29 mmol) was added to the Fmoc-Leu-OH and HOBt mixture to pre-activate the AA under stirring for 1 minute. After that, the solution was injected into stage 1 of the synthesizer via the feed-tank to initiate coupling. The stage 1 solution was circulated for 2 hours at 35 °C, maintained via a heater feedback control loop. Samples were taken from the sampling port and then diluted by 5:95 with MeCN to lower the concentration for UHPLC-MS analysis. Analysis was carried out using UHPLC-MS (*Gradient 1*). Once UHPLC-MS confirmed coupling reaction completion, piperidine (20 mL, 10 v/v%) was added slowly to the feed tank. Stage 1 was circulated for a further 30 minutes at 35°C. Once UHPLC-MS confirmed complete Fmoc removal, diafiltration was started following the OSN operation protocol described in section 7. The coupling/deprotection/diafiltration chain extension cycle described above was repeated in subsequent reactions with the following order of amino acids: Fmoc-Phe-OH, Fmoc-Ser(tBu)-OH, Fmoc-Ser(tBu)-OH, Fmoc-Tyr(tBu)-OH. H-pentapeptide-nanostar **16** (i=5) was sampled from the feed tank at a small scale (~1 mL) and evaporated to dryness under high vacuum. The dried sample was dissolved in the TFA-Et_3SiH-H_2O 95:2.5:2.5 cleavage cocktail, stirring for 2 hours. Cold diethyl ether was added to precipitate pentapeptide, and the solution was filtered. The resultant solid was air dried on the filter paper. The crude pentapeptide H-Tyr-Ser-Ser-Phe-Leu-NH_2 **17** was dissolved in 1 v/v % acetic acid aqueous buffer for analysis by UHPLC-MS (*Gradient 3*). The remaining H-pentapeptide-nanostar **16** (i=5) in the stage 1 of the synthesizer was subjected to the same coupling/deprotection/diafiltration chain extension cycle, using the same sequence of AAs as above, to obtain H-decapeptide-nanostar **18**. Compound **18** was recovered from stage 1, and the THF was evaporated at reduced pressure using a rotary evaporator. Remaining NMP was then removed with heating under high vacuum to recover gel-like peptide-star **18**. The recovered material was subjected to cleavage and global deprotection with TFA-TIS-H_2O 95:2.5:2.5 cocktail for 2 hours. Cold diethyl ether was added to precipitate the peptide, and the solution was filtered then air-dried. Crude decapeptide H-(Tyr-Ser-Ser-Phe-Leu)$_2$-NH_2 **19** was dissolved in DMF for analysis by UHPLC-MS (*gradient 3*).

The synthesis of (Ser2, Ser3)-Leu-Enkephalin was performed using i) 1.7 wt%; ii) 1wt% and iii) 3wt% of initial nanostar concentration. The 1.7 wt% synthesis was repeated to check reproducibility (see Table S5). Crude peptides (5- and 10-mer) were purchased from a solid phase synthesis supplier that uses a standard SPPS protocol with 3 equiv. amino acids. All products were analyzed with the same UHPLC-MS (*Gradient 3*) method: Purity of all samples was determined as a ratio of the main peptide peak area and the cumulative peak areas within the range of +/- 2 minutes around the main peak.

Table S5. Enkephalin-type model peptide purities.

	Peptide purity	
Conditions	Pentapeptide, **17** (%)	Decapeptide, **19** (%)
SPPS vendor (3.0eq)	97	77
SPPS vendor (1.7eq)	97	35
1.0 wt%	87	60
1.7 wt% (1st Run)	94	84
1.7 wt% (2nd Run)	93	83
3.0 wt%	93	Precipitated out

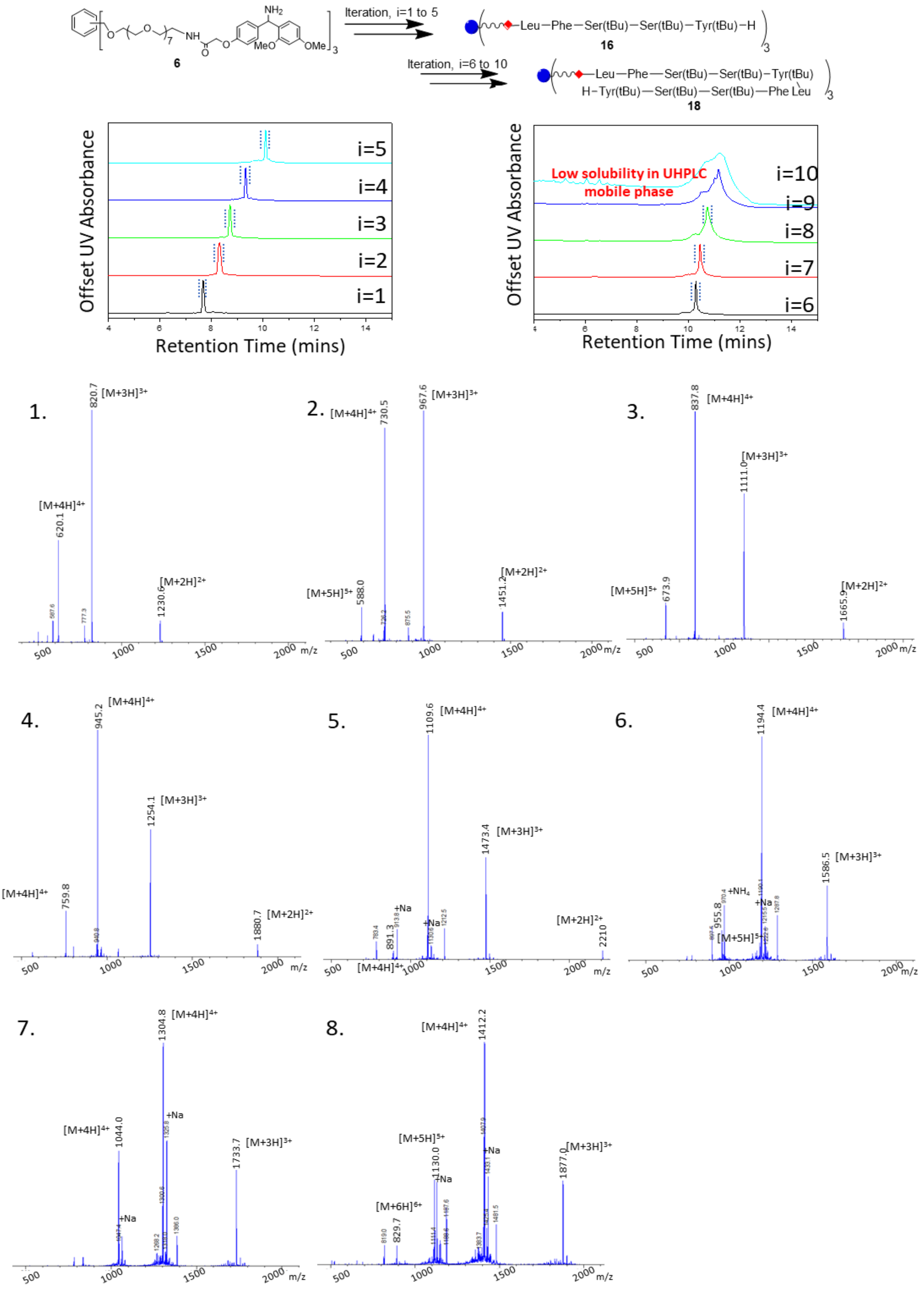

Figure S10: UHPLC-MS chromatograms (UV absorbances and corresponding mass specs) of peptide-nanostar (i=1 to 10) for (Ser2, Ser3)-Leu-Enkephalin obtained from the 1.7 wt% (1st Run).

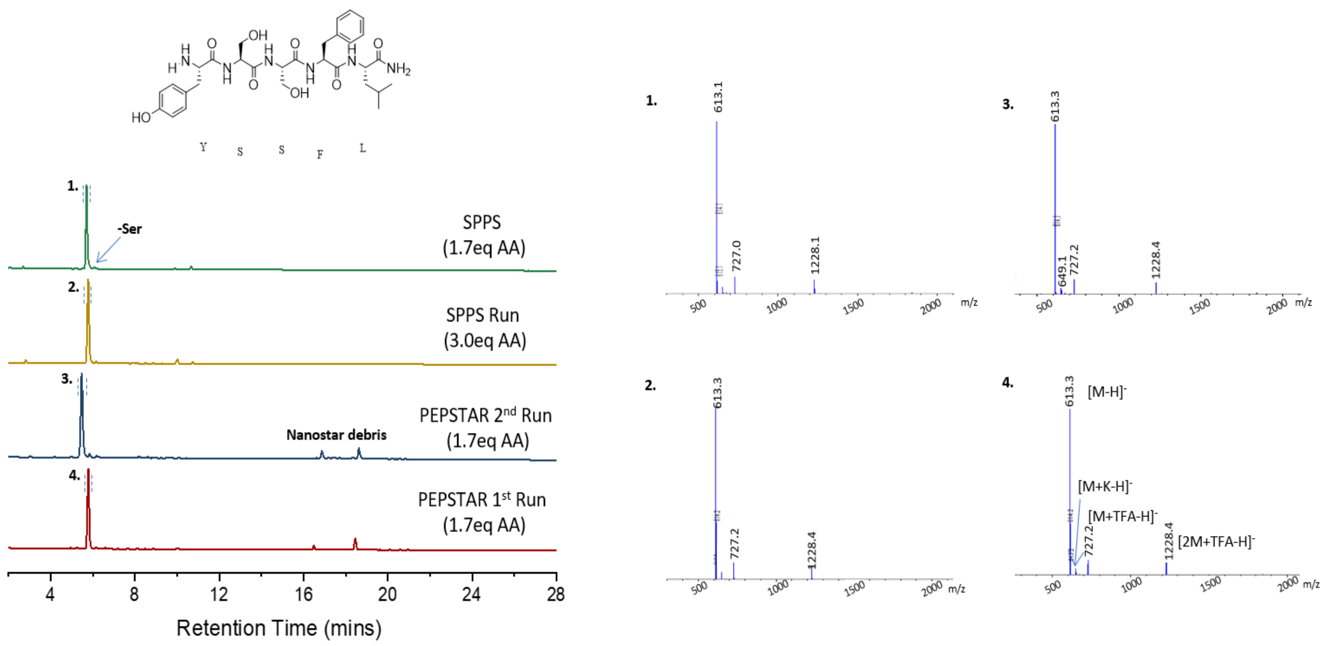

Figure S11: Comparisons of crude purities of pentapeptide H-Tyr-Ser-Ser-Phe-Leu-NH2, **18**, produced by PEPSTAR (1.7wt%), 1st and 2nd run, and SPPS vendor peptides obtained with 1.7 eq and 3.0 eq amino acids.

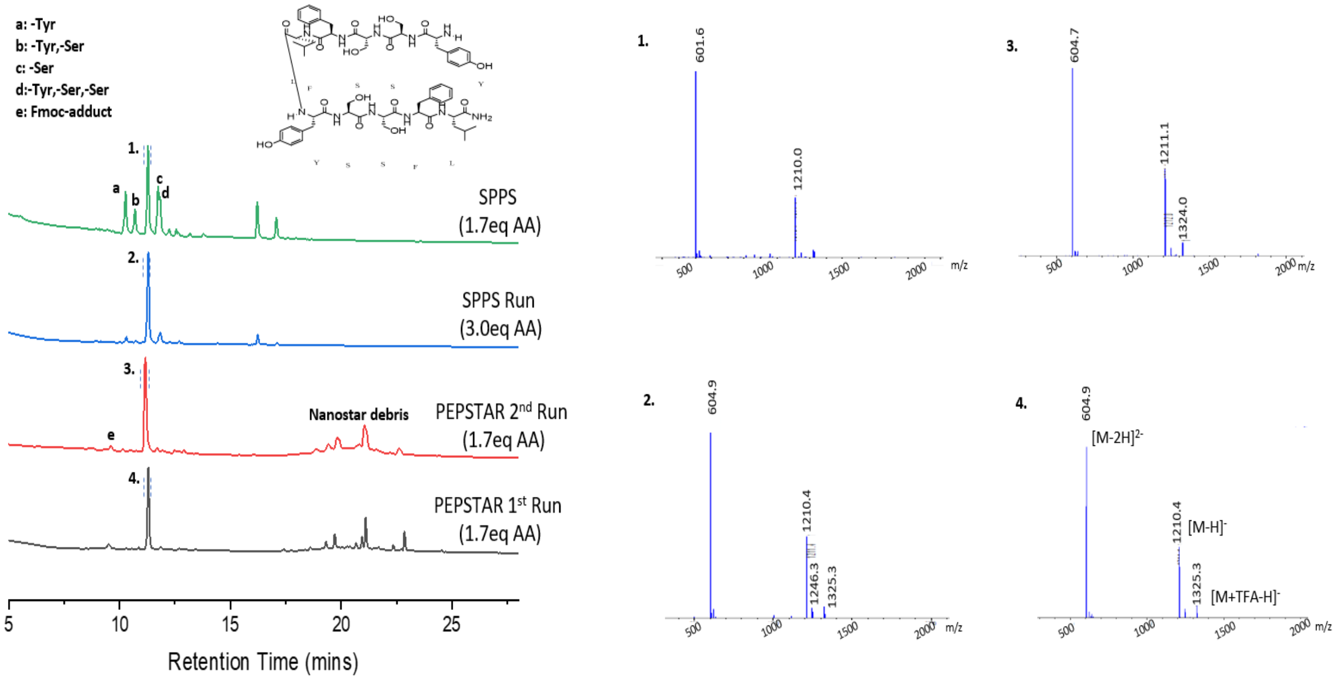

Figure S12: Comparisons of crude purities of decapeptide H-(Tyr-Ser-Ser-Phe-Leu)$_2$-NH$_2$, **19**, produced by PEPSTAR (1.7wt%), 1st and 2nd run, and SPPS vendor peptides obtained with 1.7 eq and 3.0 eq amino acids.

.

10. Octreotate amide and acid synthesis

For the synthesis of octreotate amide, 1,3,5-tris[(p-phenoxyacetamido)octa(ethyleneglycol)oxymethyl]benzene, **6** (3.0 g, 1.46 mmol, ~1.7 wt%) was gradually added to the synthesizer via the feed tank to dissolve in THF-NMP 35:65. The HPLC and gear pumps were turned on to provide effective mixing and dissolution in stage 1**.** In a separate vial, Fmoc-Thr(tBu)-OH (2.81 g, 7.29 mmol) and HOBt (1.23 g, 9.11 mmol) were dissolved in a minimum amount of solvent. Once fully dissolved, DIC (1.20 mL, 7.29 mmol) was added to the Fmoc-Thr(tBu)-OH and HOBt mixture under stirring to pre-activate the AA for 1 minute. After that, the solution was injected into stage 1 of the synthesizer via the feed. Stage 1 was circulated for 2 hours at 35 °C, controlled via a heater feedback control loop. Samples were taken from the sampling port and then diluted by 5:95 with MeCN to lower the concentration for UHPLC-MS analysis. The analysis was carried out within 16 minutes, using UHPLC-MS (*Gradient 1*). Once UHPLC-MS confirmed complete coupling, piperidine (20 mL, 10 v/v%) was added slowly to the feed tank. The stage 1 solution was circulated for a further 30 minutes at 35 °C. Once UHPLC-MS confirmed complete Fmoc deprotection, diafiltration was started, following the OSN operation protocol described in section 7. The coupling/Fmoc removal/diafiltration procedure described above was repeated in all subsequent chain extension cycles to obtain H-octreotide-nanostar **20**, with the following order of amino acids: Fmoc-Cys(Acm)-OH, Fmoc-Thr(tBu)-OH, Fmoc-Lys(Boc)-OH, Fmoc-D-Trp(Boc)-OH, Fmoc-Phe-OH, Fmoc-Cys(Acm)-OH and Fmoc-D-Phe-OH**.** Molecule **20** was recovered from stage 1, and THF was evaporated at reduced pressure using a rotary evaporator. Remaining NMP was removed with heating under high vacuum to recover gel-like octamer **20**. The recovered material was subjected to cleavage and global deprotection with widely used TFA-Et_3SiH-H_2O 95:2.5:2.5 cocktail for 2 hours. Cold diethyl ether was added to precipitate the peptide, the solution was filtered, and the collected solids were air dried. Crude peptide H-D-Phe-Cys(Acm)-Phe-D-Trp-Lys-Thr-Cys(Acm)-Thr-NH_2 was dissolved in 1 v/v % acetic acid aqueous buffer for analysis with UHPLC-MS (*gradient 3*). The purity of all samples was determined as a ratio of the main peptide peak area and the cumulative peak areas within the range of +/- 2 minutes around the main peak.

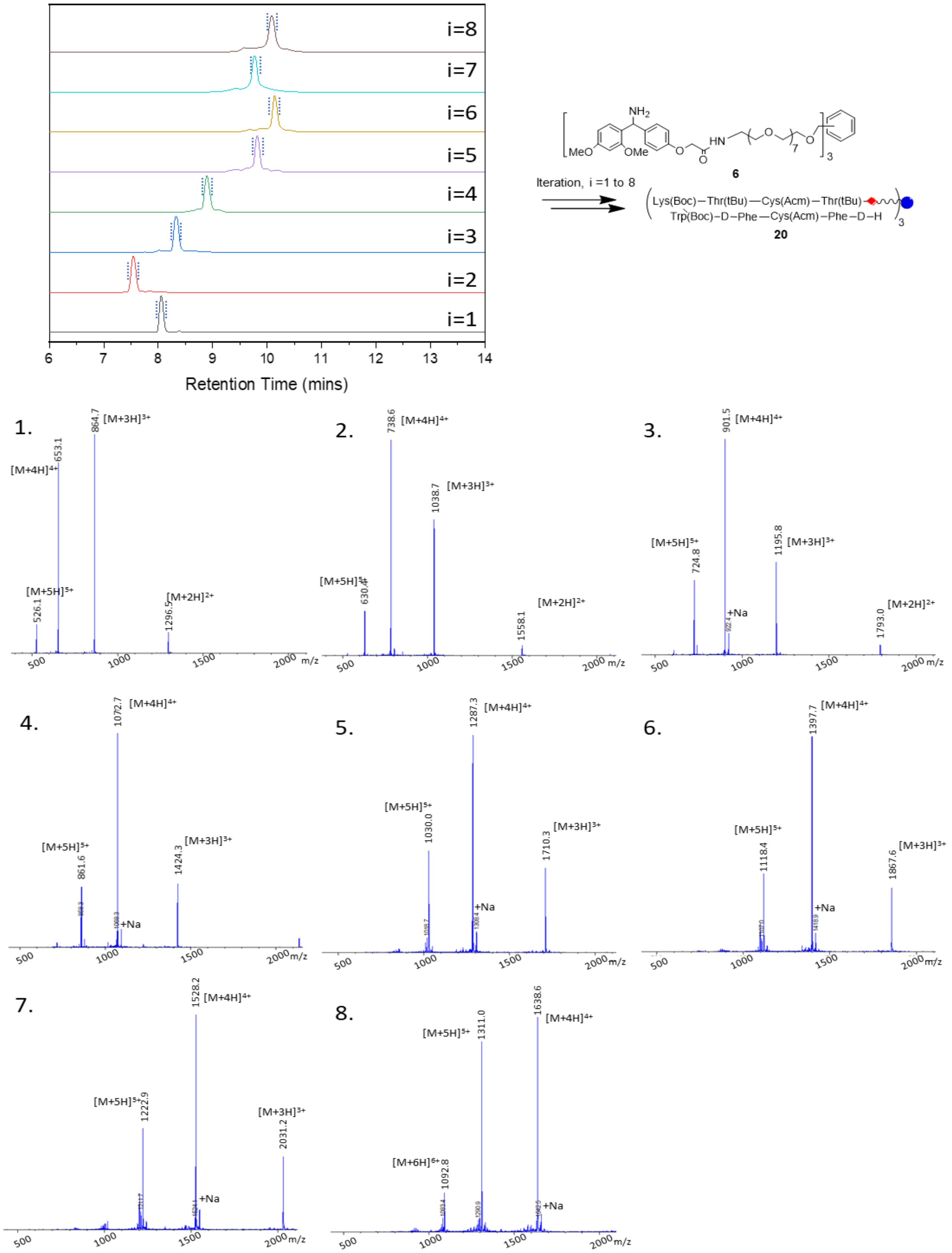

Figure S13: UHPLC-MS chromatogram (UV absorbances and corresponding mass spec) of peptide-nanostar (i=1 to 8) for linear octreotate amide.

During the synthesis of linear octreotate amide, H-tripeptide-nanostar (i=3, H-Thr(tBu)-Cys(Acm)-Thr(tBu)-Rink-nanostar) was sampled from the feed tank at a small scale (~1 mL) and evaporated to dryness under high vacuum. The dried sample was dissolved in the TFA-Et3SiH-H2O 95:2.5:2.5 cleavage cocktail, stirring for 1 hour. Cold diethyl ether was added to precipitate pentapeptide, and the solution was filtered. The resultant solid was air dried on the filter paper. The crude tripeptide H-Tyr-Cys(Acm)-Thr-NH_2 was dissolved in 1 v/v % acetic acid aqueous buffer for analysis by HPLC (see section Characterization and Measurements). The standard H-Thr-D-Cys(Acm)-Thr-NH_2 obtained on solid phase was also dissolved in 1v/v% acetic aqueous buffer for analysis by HPLC using the same gradient. A mixture of H-Tyr-Cys(Acm)-Thr-NH_2 obtained from PEPSTAR and H-Thr-D-Cys(Acm)-Thr-NH_2 standard was also prepared for analysis by HPLC. The result below showed that <0.1% of cycsteine epimerization from L- to D- occurred during the synthesis of octreotate amide (i=3).

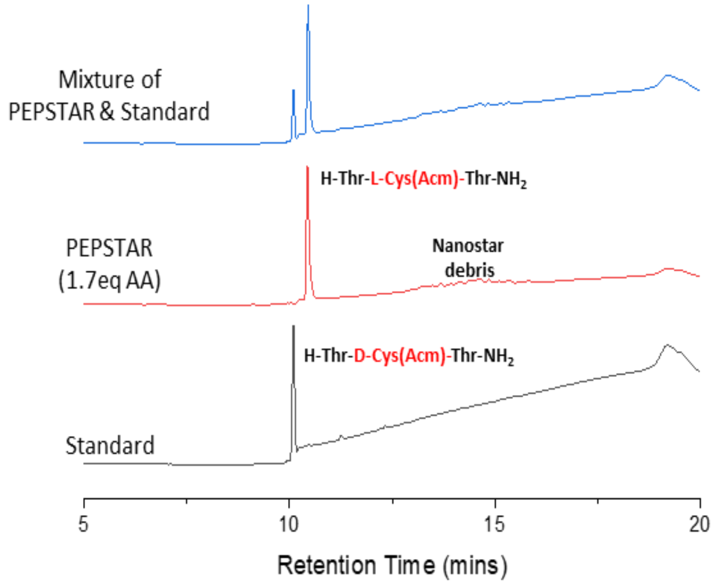

Figure S14: HPLC chromatogram (UV absorbances) of H-Thr-L-Cys(Acm)-Thr-NH_2 obtained from PEPSTAR run for linear octreotate amide (i=3), H-Thr-D-Cys(Acm)-Thr-NH_2 obtained on solid phase, and the mixture of both.

For the synthesis of octreotate acid the procedure for the first chain extension was modified to minimize yield loss due to diketopiperazine cleavage. H-Thr(tBu)-O-Wang-nanostar **8** (2.8 g, 1.39 mmol) peptide-nanostar was first transferred to the feed tank dissolved in THF-NMP 35:65 (30 mL) to give a concentration around 10 wt%. Conducting the coupling entirely within the feed tank, Fmoc-Cys(Acm)-OH (2.88 g, 6.95 mmol), HOBt (1.18 g, 8.69 mmol) and DIC (1.09 mL, 6.95 mmol) were added to the solution of peptide-nanostar **8**. After stirring for 1 hour, Fmoc removal was initiated by the addition of piperidine (3 mL, giving 10 v/v%). Upon completionFmoc removal, the reaction mixture was immediately circulated into stage 1 of the synthesizer, thus lowering the nanostar concentration from 10 wt% to the 1.5 wt%, and piperidine from 10 v/v% to 1.5 v/v%, and diafiltration was initiated. After purification of the H-dipeptide-nanostar (i=2), in a separate vial Fmoc-Thr(tBu)-OH (2.76 g, 6.95 mmol) and HOBt (1.18 g, 8.69 mmol) were dissolved in a minimum amount of solvent. Once fully dissolved, DIC (1.09 mL, 6.95 mmol) was added to the Fmoc-(tBu)-OH and HOBt mixture under stirring to pre-activate the AA for 1 minute. After that, the solution was injected into stage 1 of the synthesizer via the feed tank to initiate the coupling reaction. Stage 1 was circulated for 2 hours at 35°C, controlled via a heater feedback control loop. Samples were taken from the sampling port and then diluted by 5:95 with MeCN to lower the concentration for UHPLC-MS analysis. The analysis was carried out within 16 minutes by UHPLC-MS (*Gradient 1*). Once UHPLC-MS confirmed coupling was complete, piperidine (20 mL, 10 v/v%) was added slowly to the feed tank. Stage 1 was circulated for a further 30 minutes at 35°C. Once UHPLC-MS confirmed complete deprotection, diafiltration was started by following the OSN protocol in section 7. The coupling/Fmoc removal /diafiltration procedure chain extension cycle described above was repeated to obtain H-octreotide-nanostar **21**, with the following order of amino acids: Fmoc-Lys(Boc)-OH, Fmoc-D-Trp(Boc)-OH, Fmoc-Phe-OH, Fmoc-Cys(Acm)-OH and Fmoc-D-Phe-OH**.** Molecule **21** was recovered from stage 1, and THF was evaporated under reduced pressure using a rotary evaporator. Remaining NMP was removed with heating under high vacuum to recover gel-like octapeptide-nanostar **21**. The recovered material was subjected to cleavage and global deprotection with TFA-H_2O 95:5 for 1 hour. Cold diethyl ether was added to precipitate peptide, the solution was filtered, and the solids collected were air dried. Crude peptide H-D-Phe-Cys(Acm)--Phe-D-Trp-Lys-Thr-Cys(Acm)-Thr-OH was dissolved in aqueous HOAc (1%) for analysis by UHPLC-MS (*gradient 4*). The purity of all samples was determined as a ratio of the main peptide peak area and the cumulative peak areas within the range of +/- 2 minutes around the main peak.

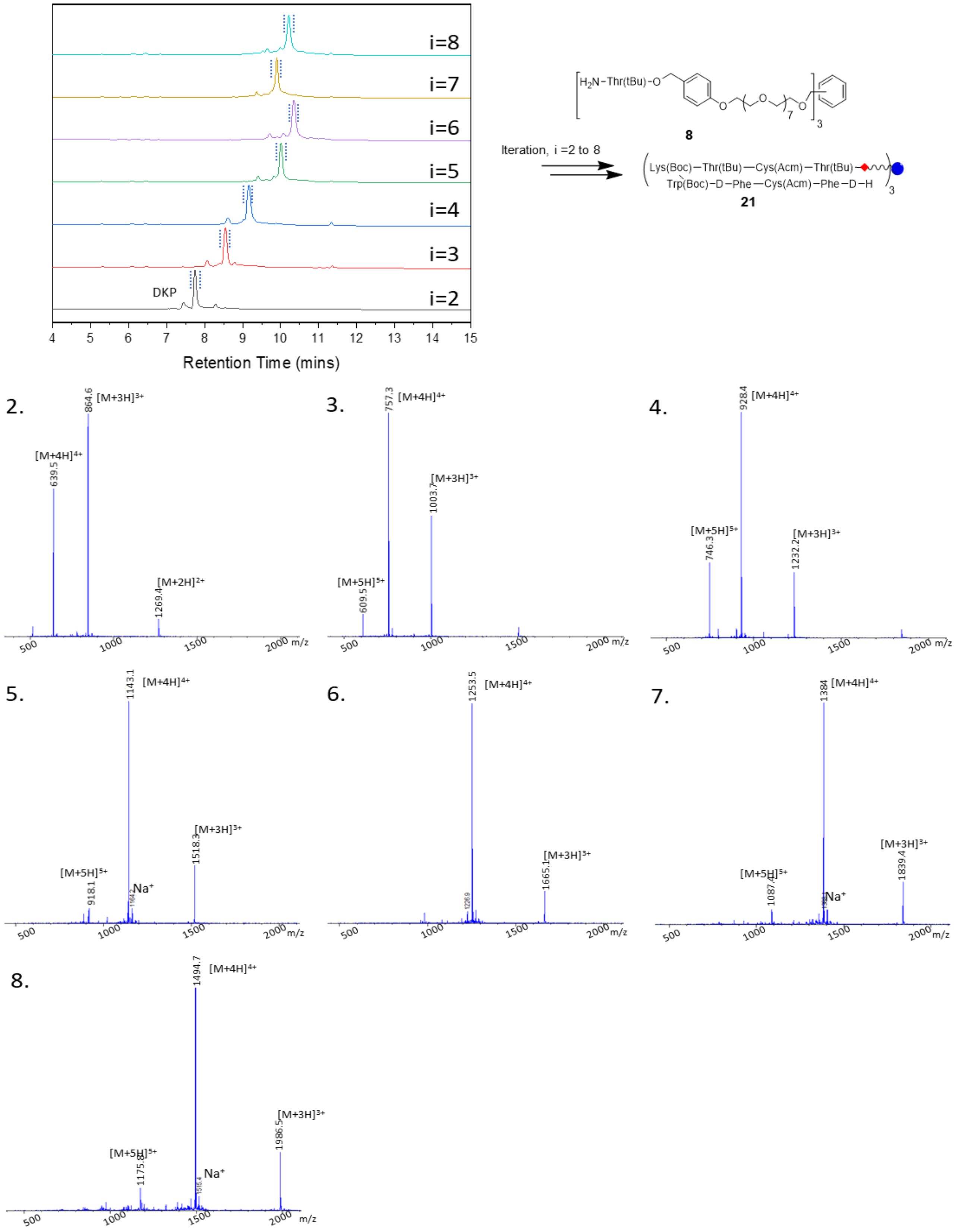

Figure S15: UHPLC-MS chromatogram (UV absorbances and corresponding mass spec) of peptide-nanostar (i=2 to 8) for linear octreotate (acid).

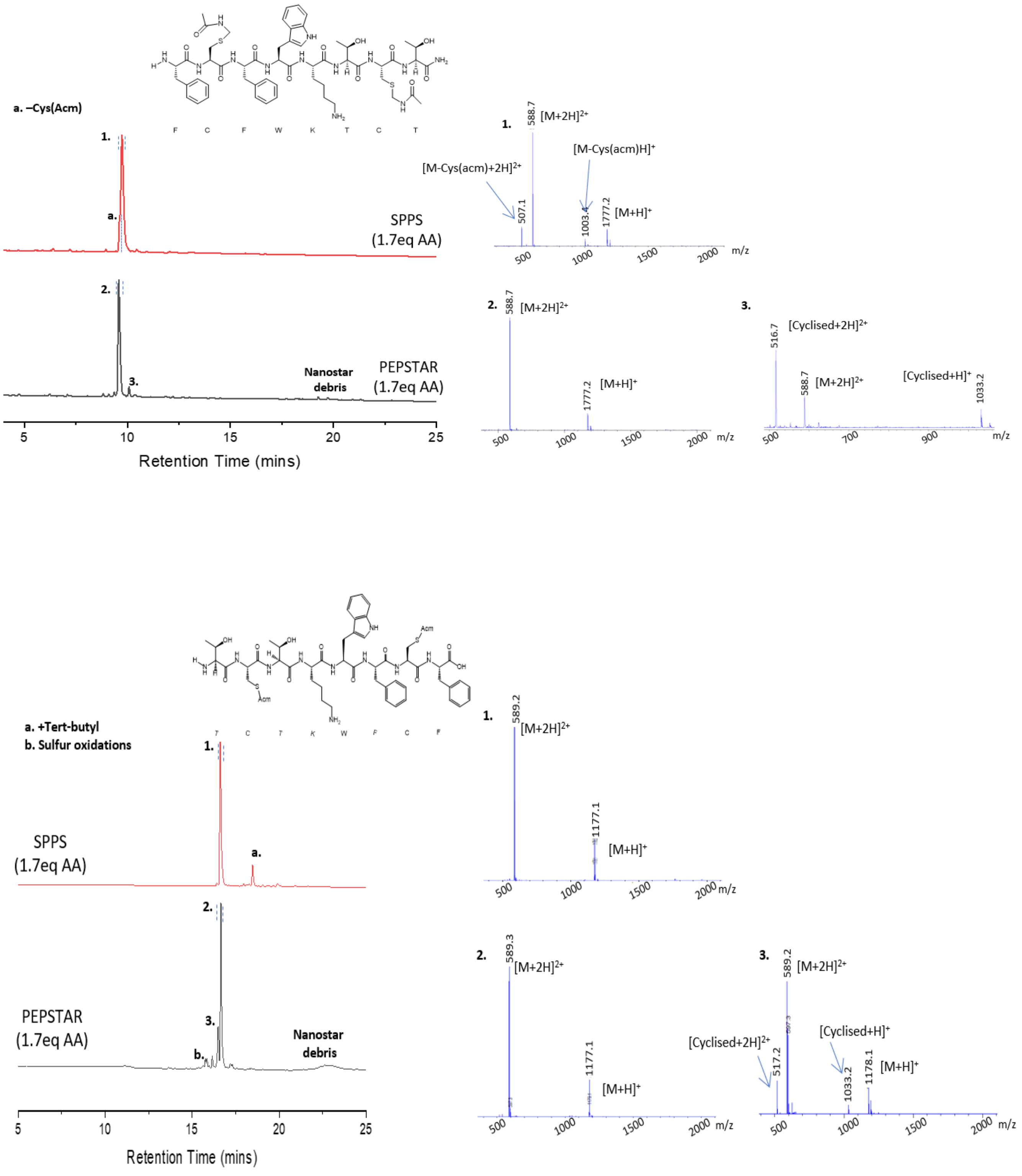

Figure S16: Crude purity of linear octreotate amide (top) and linear octreotate (bottom) produced by PEPSTAR and SPPS vendor with 1.7eq of AA. UHPLC-MS (*Gradient 3)* and *(Gradient 4)* was used for the analysis.

11. Solid phase synthesis protocol

Pour Fmoc-AA-O-Resin into a reaction column. Sufficient DMF is added to immerse and swell the resin for 30 minutes, then the DMF is drawn through the sinter under suction until dry. Piperidine-DMF 1:4 is added to initiate Fmoc removal, bubbling through nitrogen from below to agitate for 30 minutes, then dry by suction. Add DMF, agitating with nitrogen for 2 minutes, then suck dry; repeat this procedure 6 times to wash off the piperidine. Add 3 molar equivalents of protected amino acid to the resin in the reaction column, then 2.85 molar equivalents of HBTU, followed by 6 molar equivalents of N-methyl morpholine (NMM), bubbling nitrogen to agitate for 30 minutes, then suck dry. Wash the resin with DMF, agitating with nitrogen for 2 minutes, then suck dry, repeating this procedure 3 times.

This process is repeated until the target sequence is reached. After coupling the last amino acid and Fmoc-deprotection, suck dry, then add methanol to the reaction column, bubbling nitrogen to agitate for 2 minutes, suck dry. Next, add DCM, bubbling nitrogen to agitate for 2 minutes, then suck dry; repeat this procedure 3 times. Finally add methanol, bubbling nitrogen to agitate for 2 minutes, then suck dry; repeat the procedure 2 times. The resin is next transfered to a vacuum chamber and dried for 12 hours before cleavage. The dried resin is placed in a round-bottom flask to which is added cleavage cocktail (1 g per 10 mL), and the flask is placed on a fixed temperature rocking bed at 25 °C for 2 hours. A glass sinter funnel is used to separate the resin. To the briskly stirred filtrate was is added 6-8 volumes of dry ether. The resultant white suspension of precipitated crude peptide is placed in centrifuge tubes, which are spun at 4000 rpm for 3 minutes. The supernatant is removed, further ether is added while stirring with a glass rod, and the suspension is centrifuged again; repeat 5 times. Finally, the peptide is lyophilized for 24 hours.

Post-synthesis purification was omitted by the vendor to obtain only crude peptides. To examine the crude purities at lower molar equivalents of amino acids, similar to that of PEPSTAR, the procedures were repeated with 1.7 equivalents of protected amino acids with correspondingly scaled molar equivalents of HBTU and NMM.

12. Process Mass Intensity (PMI) and cost of materials

PMI was evaluated for both PEPSTAR and MEPS in the scenario of synthesizing crude linear octreotide amide. MEPS reported using polydisperse 5kDa mPEG [Poly(ethylene glycol) mono-methyl ether] as the anchor, 2 equivalents of amino acid per coupling and a total of 6085 $L.mol^{-1}$ of DMF for a 5 AA peptide [3]. This is equivalent to 1217.1 $L.mol^{-1}$ of DMF per synthesis cycle, including both post-coupling and post-deprotection diafiltration. To simplify the PMI evaluation, MEPS was assumed to produce peptide with similar product quality and yield for linear octreotide amide as PEPSTAR. For PEPSTAR, the initial nanostar concentration was set as 2 wt% and maintained at 2 L of THF-NMP 35:65 for diafiltration per synthesis cycle. To compare the efficiency to solid phase, the following assumption was made: H-Rink amide ChemMatrix® Resin (0.40-0.60 $mmol.g^{-1}$ loading), 3.0 equivalents of amino acids. The solvent consumption of around 200 $L.mol^{-1}$ of DMF per synthesis cycle was based on the reported value of Pawlas et al. [4]. The yield of crude peptide is estimated to be around 75% based on the information given by the SPPS vendor.

PMI is calculated for producing per mol of linear octreotide amide with the following equation[5].

$$Equation\ S13 : PMI = \frac{Total\ mass\ in\ a\ process(kg)}{Mass\ of\ product\ (kg)}$$

Table S6. PMI of MEPS, PEPSTAR and SPPS for producing 1 mol of crude linear octrotate amide (1716 $g.mol^{-1}$).

g/mol	MEPS	PEPSTAR	SPPS
Anchor	5000	707	2000
Amino acid	6788	5770	10182
Solvent Usage	$9.19x10^6$	$2.80x10^6$	$1.51x10^6$
Yield %	80	80	75
Final Product	940.8	940.8	882
**PMI**	**9783**	**2983**	**1726**

To compare their respective economics, the reagent costs were estimated, which includes the costs of anchor, amino acids and solvents to produce 1 mol of linear octreotide amide. The conditions used to evaluate E-factors were kept similar. The cost to synthesize H-Rink Nanostar was estimated by adding up all the reagents used per gram of anchor, without accounting for the capital and operational costs. The reagent costs were obtained from our lab's commercial suppliers. The costs for 1 mol of product could be significantly lower than the estimates if the reagents sourced on an industrial scale.

Table S7. Cost of materials for MEPS, PEPSTAR and SPPS for producing 1 mol of linear octrotide amide (1716 $g.mol^{-1}$).

£/mol	MEPS	PEPSTAR	SPPS
Anchor *	672	35,500	124,600
Amino acid **	76,431	61,458	114,647
Solvent Usage ***	101,115	61,458	18,603
Yield %	80	80	75
**Product**	**222,773**	**202,406**	**343,799**

* Poly(ethylene glycol) mono-methyl ether for £ 0.13/g and H-Rink amide ChemMatrix® Resin (0.40-0.60 $mmol.g^{-1}$ loading) for £ 62.3/g were obtained from *Sigma-aldrich* website (June, 2020). H-Rink-Nanostar for £ 50.2/g was estimated by adding all the reagents costs with stepwise yields mentioned in *Nanostar Design and Synthesis* section. 1 gram of H-Rink-Nanostar produces around $1.414x10^{-3}$ mol of peptide.

** Fmoc-Thr(tBu)-OH (£ 2.06/g), Fmoc-Cys(Acm)-OH (£ 10.12/g), Fmoc-Lys(Boc)-OH (£ 2.2/g), Fmoc-D-Trp-OH (£41.6/g), Fmoc-Phe-OH (£ 0.66/g), Fmoc-D-Phe-OH (£12.9/g) were obtained from from *Sigma-Aldrich* website (June, 2020).

*** NMP (£ 10.4/L), THF (£ 44.2/L), DMF (£10.5/L) were obtained from *FischerSci* (June, 2020) for 25L scale.

13. References

[1] T.-L. Hwang, K. Ranganathan, Y.-Q. Fang, R. D. Crockett, S. Osgood, S. Cui, Organic Process Research & Development 2018, 22, 1007-1014.
[2] J. F. Kim, A. M. Freitas da Silva, I. B. Valtcheva, A. G. Livingston, Separation and Purification Technology 2013, 116, 277-286.
[3] S. So, L. G. Peeva, E. W. Tate, R. J. Leatherbarrow, A. G. Livingston, Organic Process Research & Development 2010, 14, 1313-1325.
[4] J. Pawlas, J. H. Rasmussen, Green Chemistry 2019, 21, 5990-5998.
[5] C. Jimenez-Gonzalez, C. S. Ponder, Q. B. Broxterman, J. B. Manley, Organic Process Research & Development 2011, 15, 912-917.

Author Contributions

Jet Yeo, Ludmila Peeva, Piers Gaffney and Andrew Livingston conceived the project and designed the experiment. Jet Yeo and Piers Gaffney designed, synthesized and characterized the nanostar anchors. Jet Yeo, Ludmila Peeva and Piers Gaffney designed the synthesis cycle. Jet Yeo and Ludmila Peeva carried out the membrane screening, as well as designed and built the synthesizer. Daeok Kim fabricated the PBI and PEI membranes. Seoyeon Chung carried out the experiments on validation of reaction kinetics with various solvents. Jet Yeo and Ludmila Peeva carried out the peptide synthesis and diafiltration for PEPSTAR. Carla Luciani, Sergey Tsukanov, Kevin Seibert, Michael Kopach provided technical advice on peptide synthesis, chemical analysis and economic analysis. Fernando Albericio designed the target peptide sequences and provided technical advice. Jet Yeo, Ludmila Peeva and Piers Gaffney wrote the manuscript. Andrew Livingston and Carla Luciani guided the project.

Appendix 1a

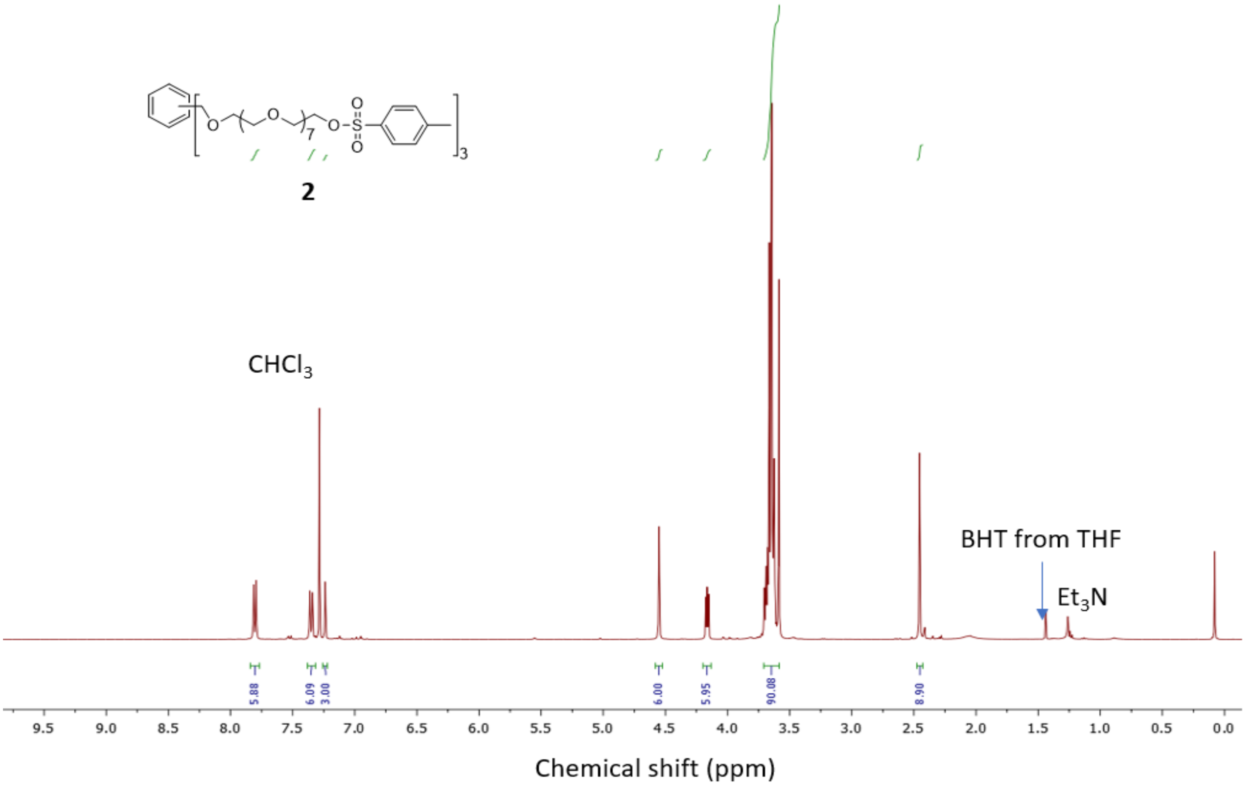

Appendix 1b

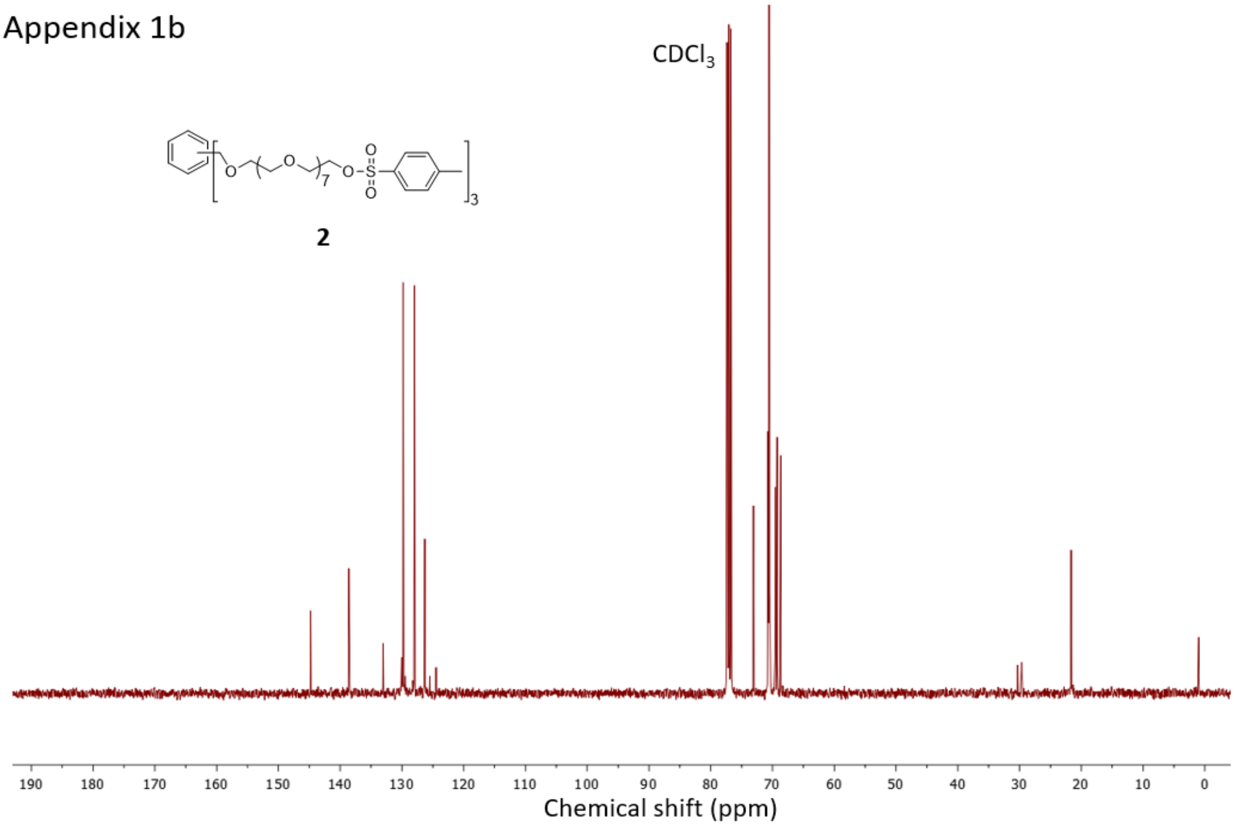

Appendix 2a

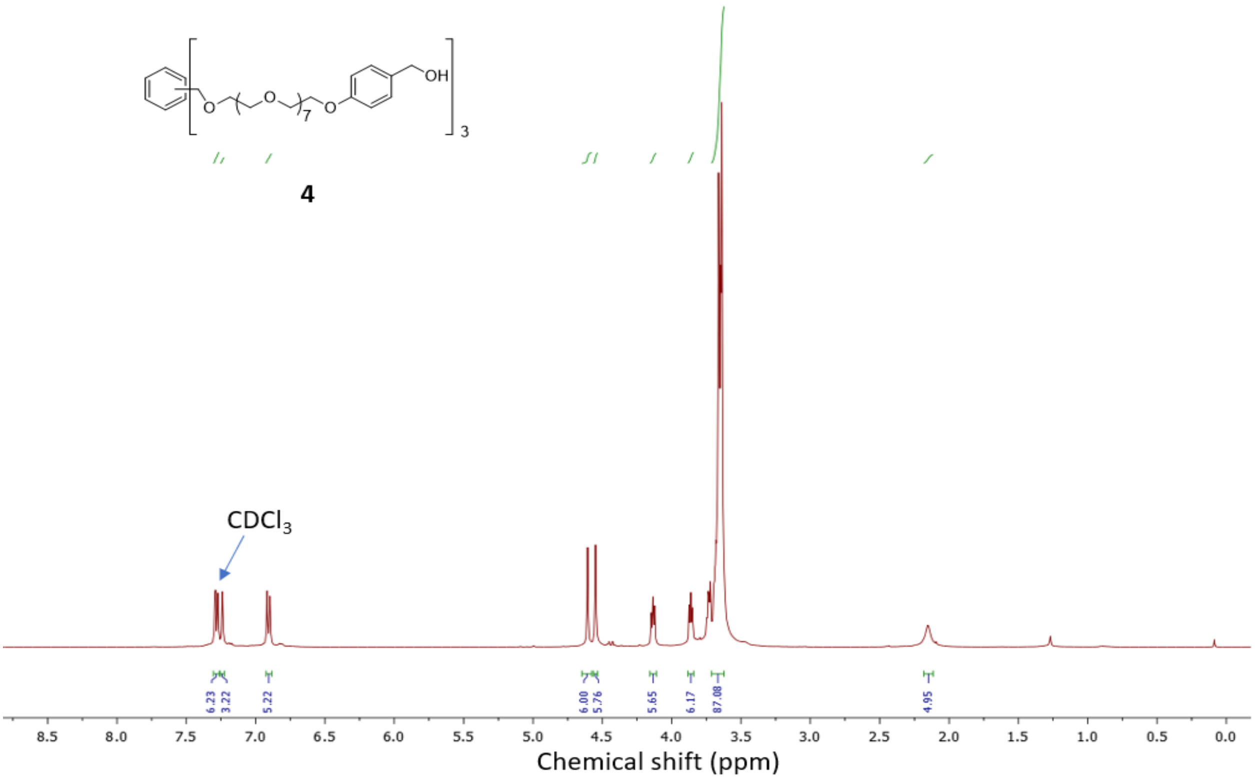

Appendix 2b

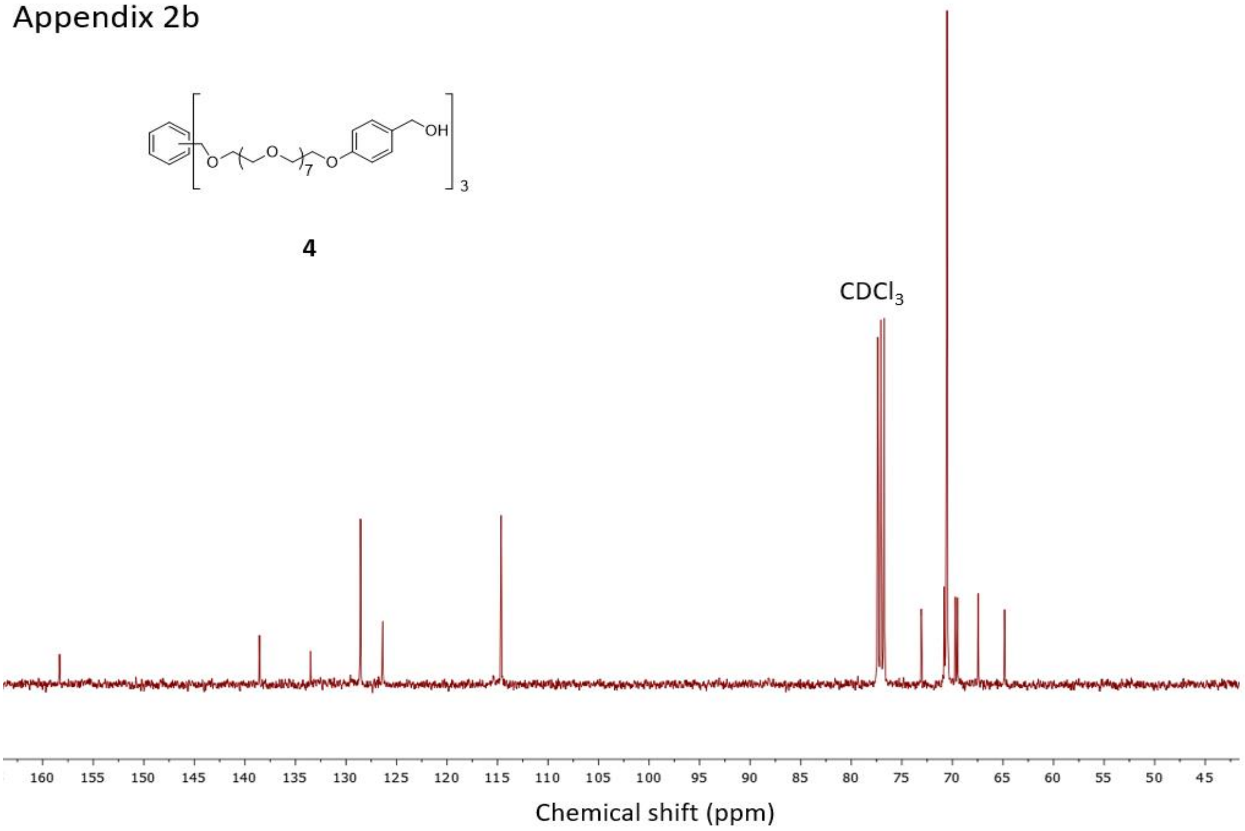

Appendix 3a

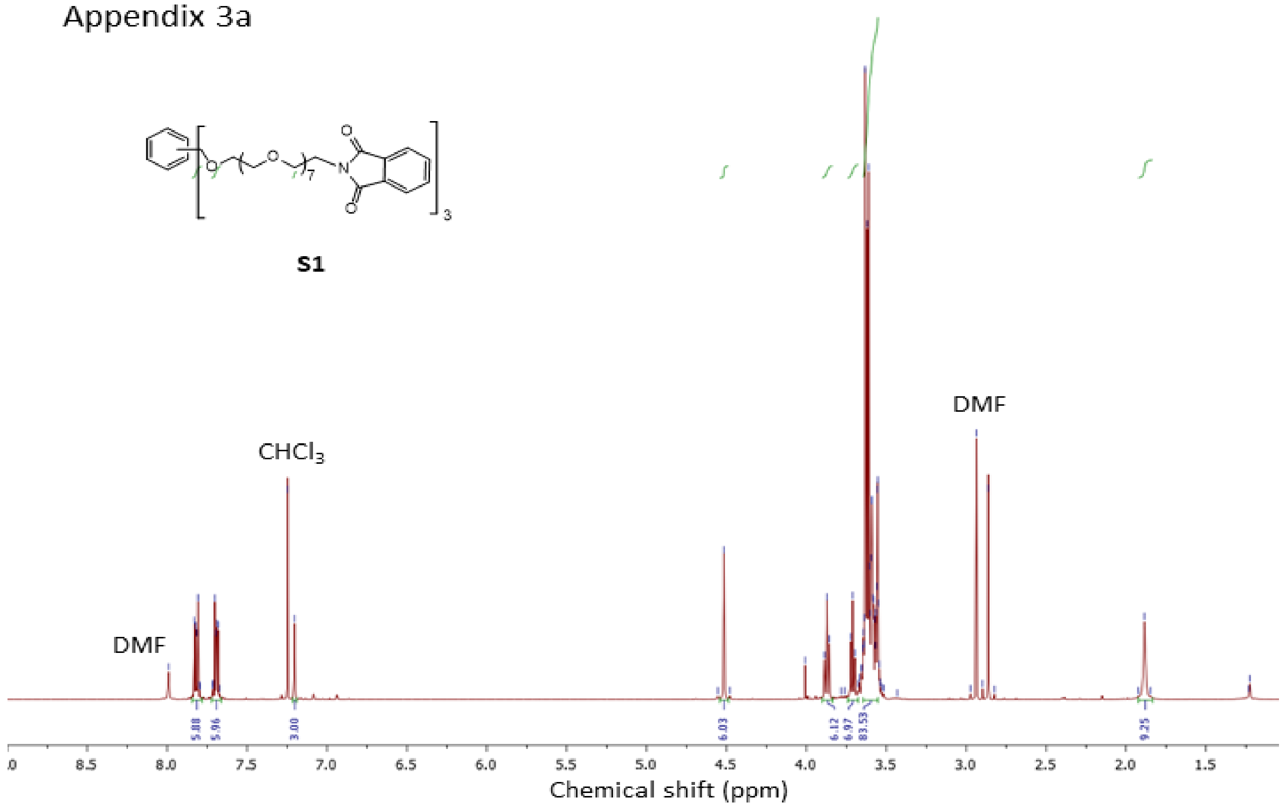

Appendix 3b

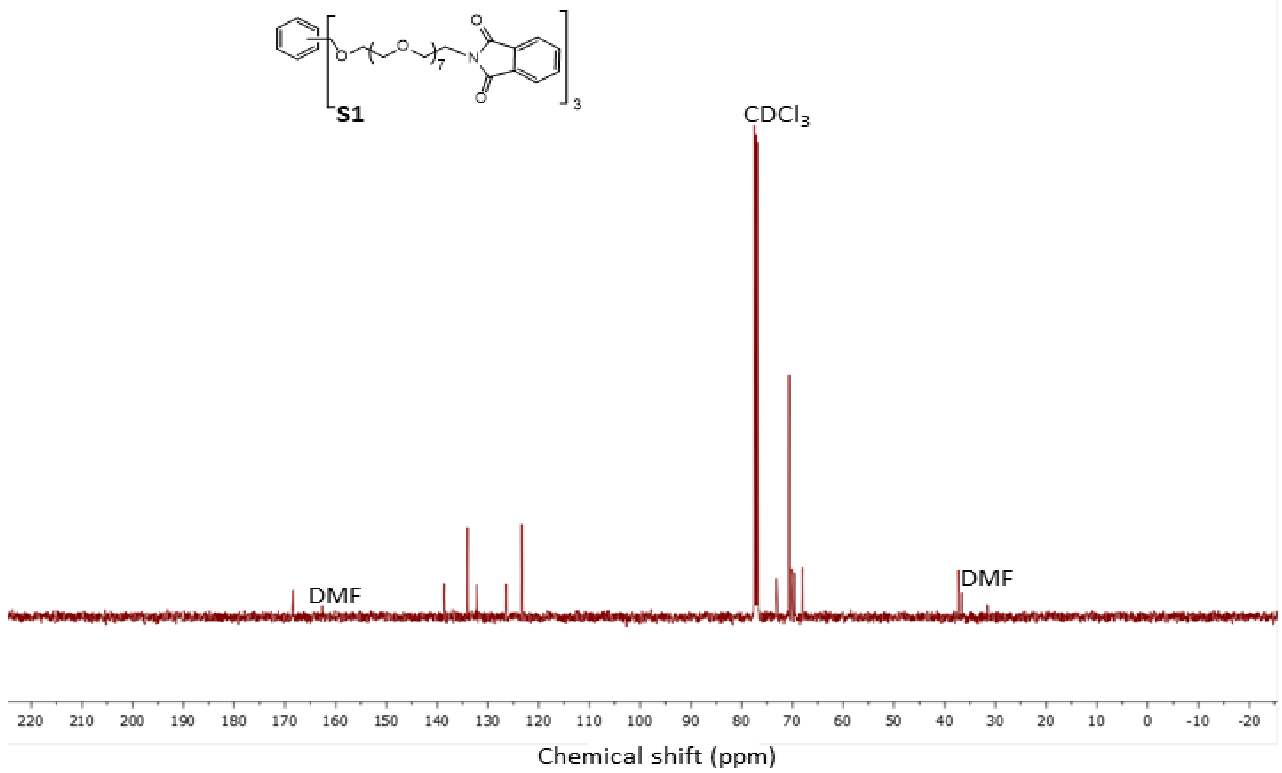

Appendix 4a

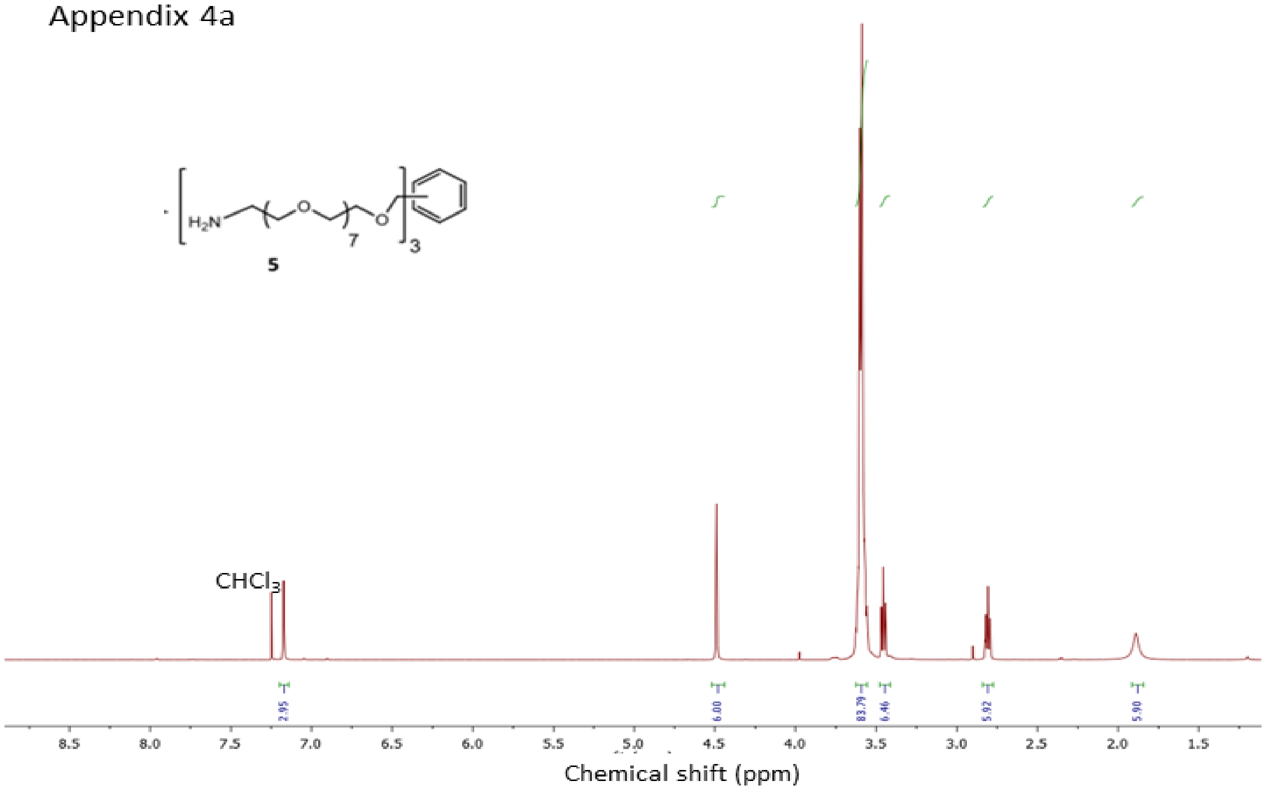

Appendix 4b

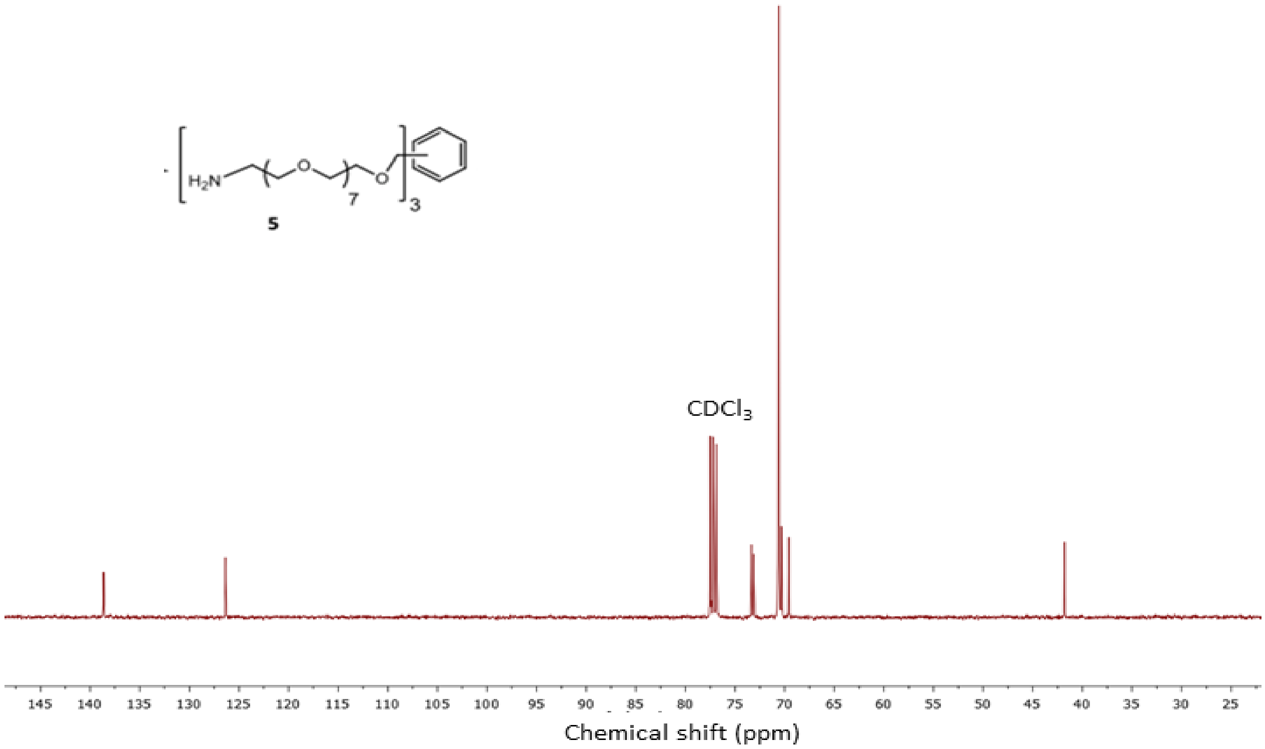

Appendix 5a

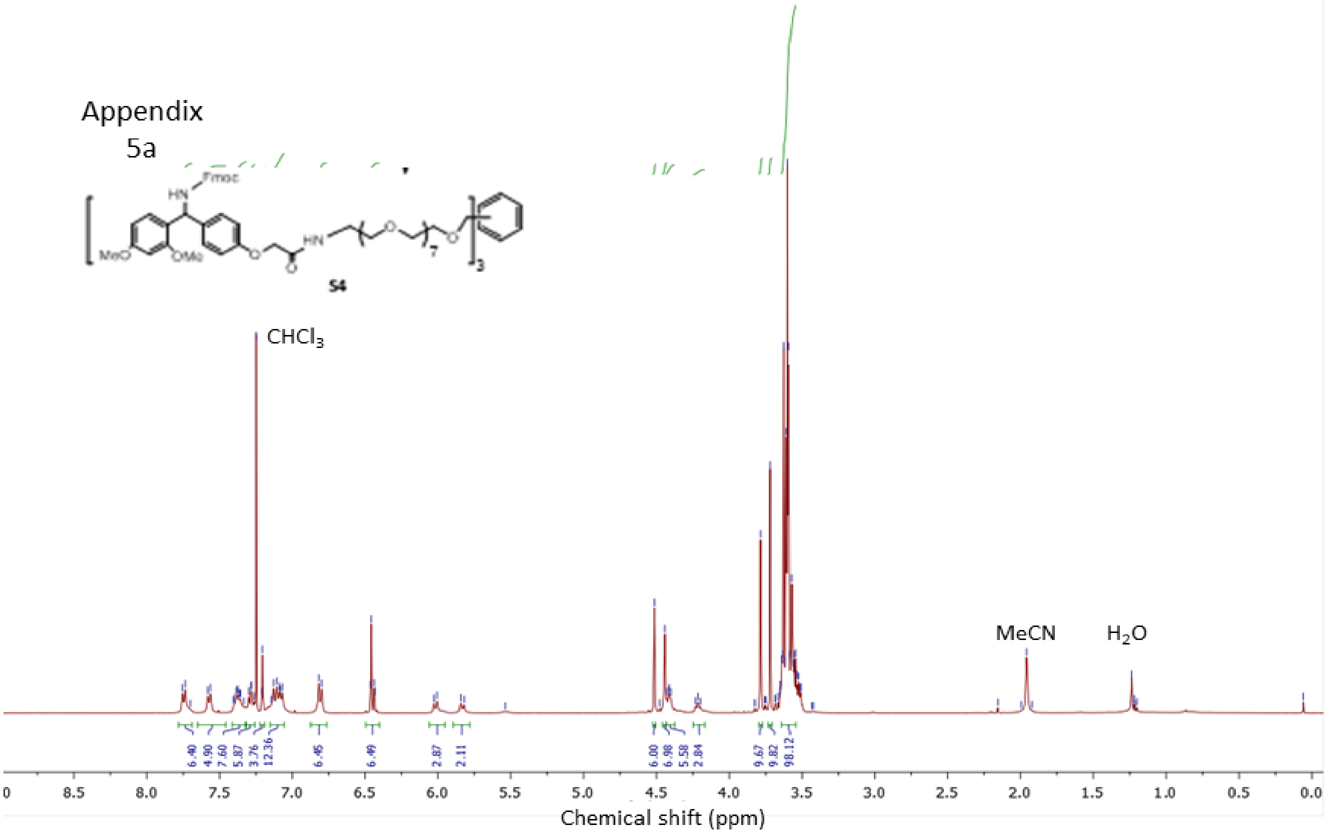

Appendix 5b

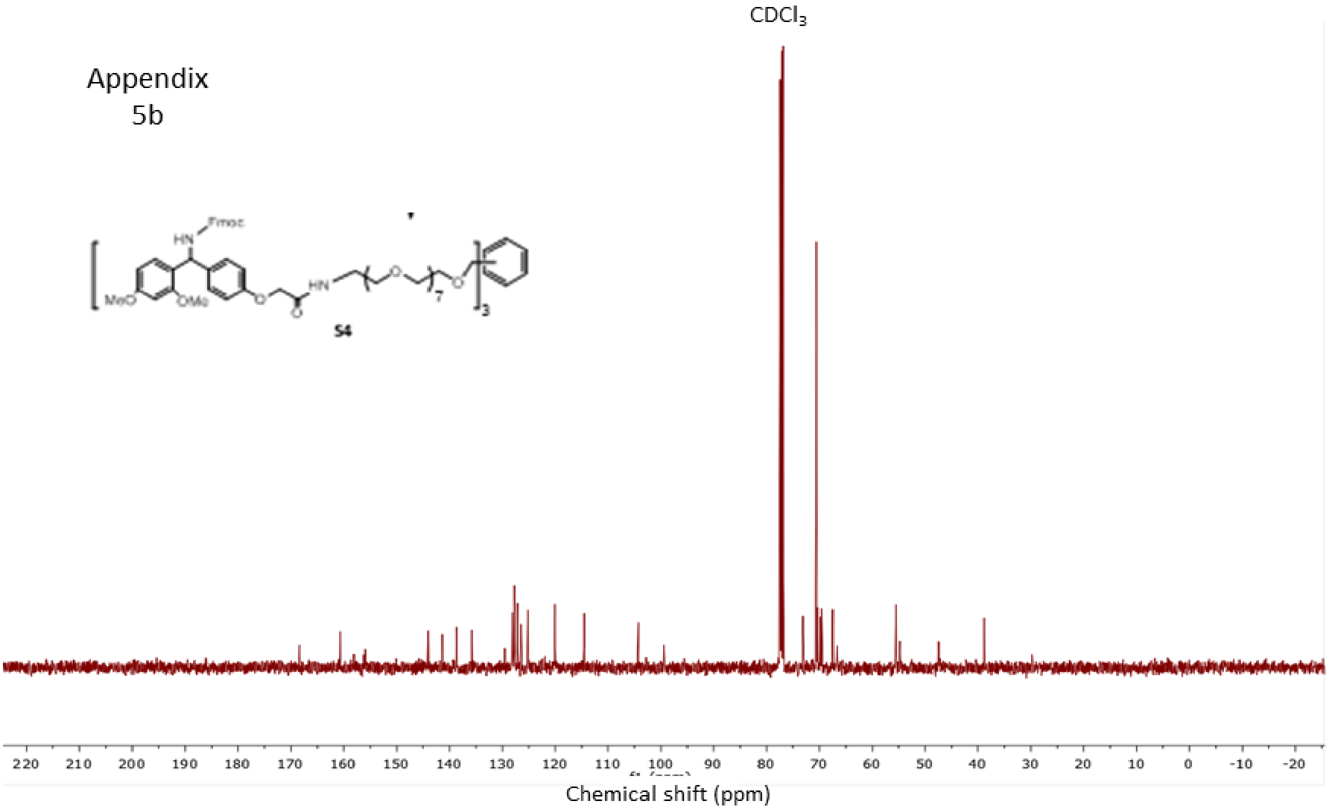

Appendix
6a

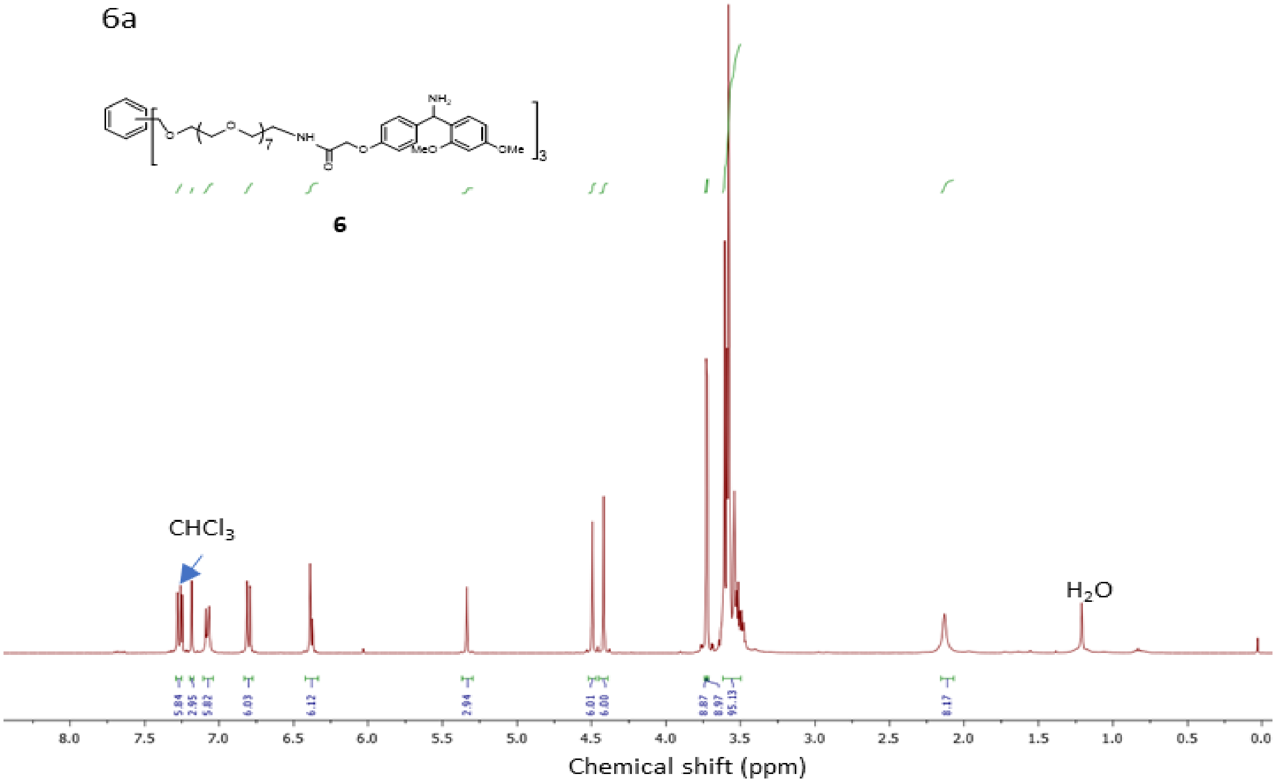

Appendix
6b

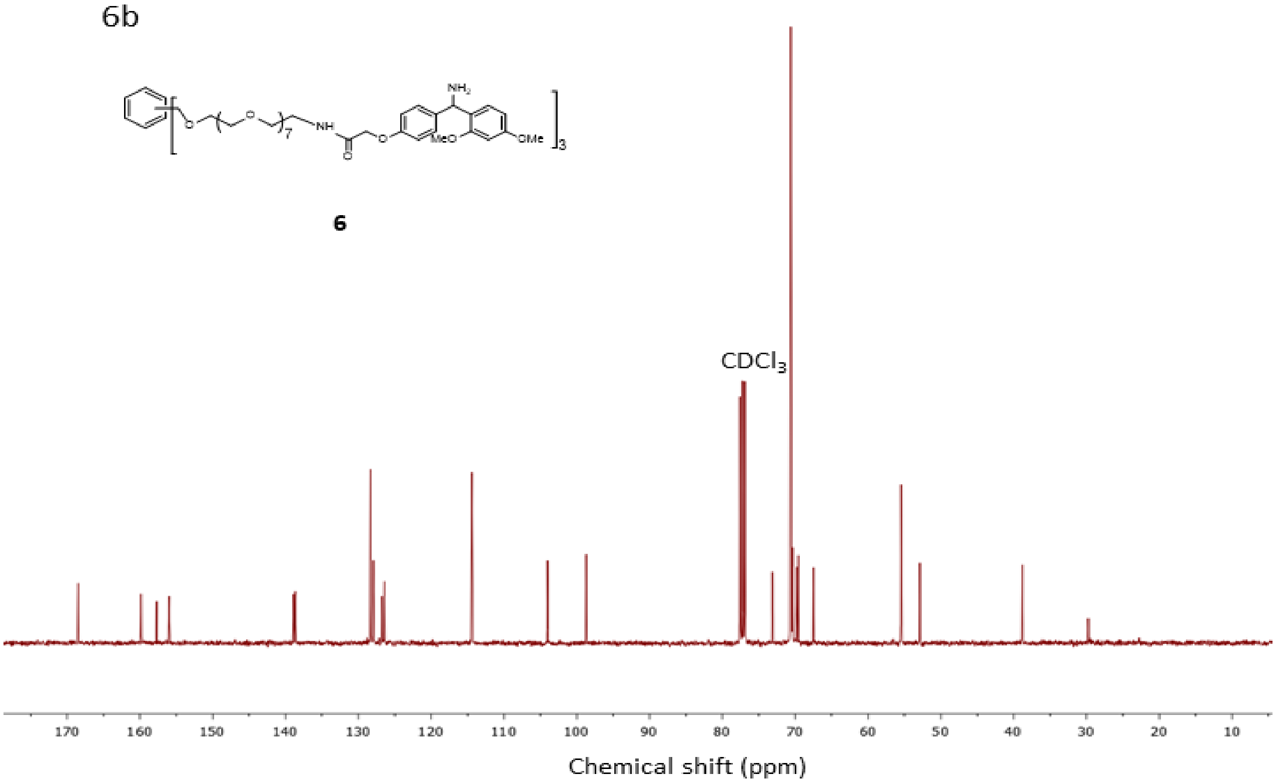

Appendix 7a

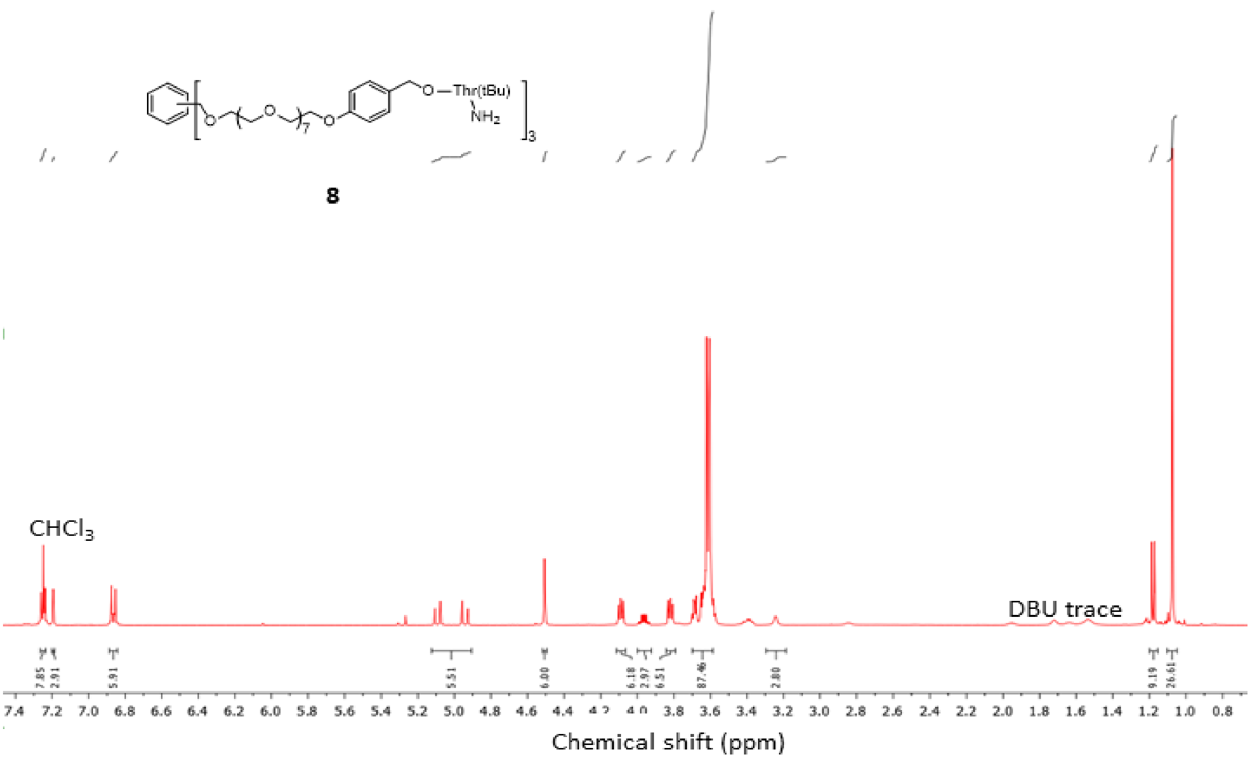

Appendix 7b

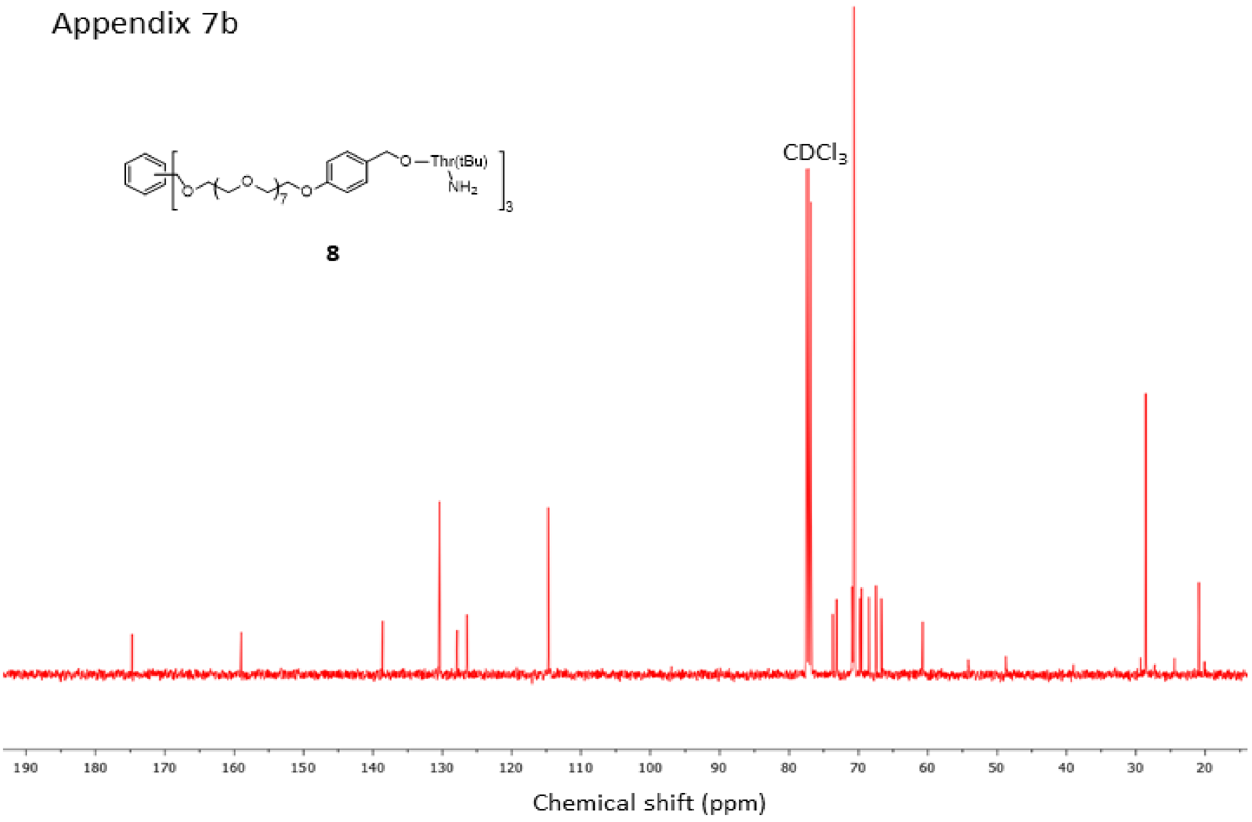

Supplement: Supplementary file 1 — Supplementary [file ANIE-60-7786-s001.pdf]
